# Spatiotemporal regulation of organelle transport by spindle position checkpoint kinase Kin4

**DOI:** 10.1242/jcs.261948

**Published:** 2024-11-13

**Authors:** Lakhan Ekal, Abdulaziz M. S. Alqahtani, Kathryn R. Ayscough, Ewald H. Hettema

**Affiliations:** ^1^ School of Biosciences, University of Sheffield, Sheffield S10 2TN, UK; ^2^ Department of Biology, Faculty of Science, University of Bisha, P.O. Box 551, Bisha 61922, Saudi Arabia

**Keywords:** Organelle inheritance, Spindle position checkpoint, SPOC, p21-activated kinase, PAK, Vacuole, Peroxisome

## Abstract

Asymmetric cell division in *Saccharomyces cerevisiae* involves class V myosin-dependent transport of organelles along the polarised actin cytoskeleton to the emerging bud. Vac17 is the vacuole/lysosome-specific myosin receptor. Its timely breakdown terminates transport and results in the proper positioning of vacuoles in the bud. Vac17 breakdown is controlled by the bud-concentrated p21-activated kinase Cla4, and the E3-ubiquitin ligase Dma1. We found that the spindle position checkpoint kinase Kin4 and, to a lesser extent, its paralog Frk1 contribute to successful vacuole transport by preventing the premature breakdown of Vac17 by Cla4 and Dma1. Furthermore, Kin4 and Cla4 contribute to the regulation of peroxisome transport. We conclude that Kin4 antagonises the Cla4/Dma1 pathway to coordinate spatiotemporal regulation of organelle transport.

## Teaser

**Highlighted Article:** Yeast vacuole transport from mother cell to bud terminates in large buds through degradation of the myosin adaptor Vac17. The SPOC kinase Kin4 contributes to vacuole inheritance by preventing premature Vac17 degradation.

## INTRODUCTION

**Fig. 1.**
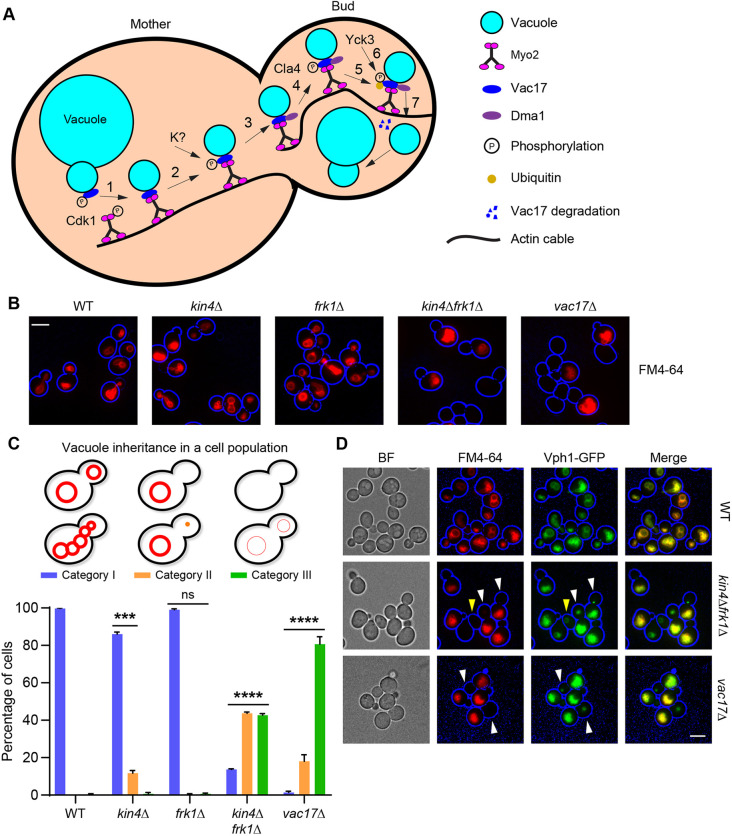
**Kin4 and Frk1 are redundant in vacuole transport to the bud.** (A) Schematic showing a cascade of events involved in vacuole transport. Vacuole transport is triggered by Cdk1-dependent phosphorylation of Myo2 and Vac17, which leads to formation of the Myo2−Vac17 transport complex (1). The Vacuole-loaded Myo2 motor moves along actin cables towards the growing bud to deliver vacuoles. Dma1 (an E3-ubiquitin ligase) recruitment in the bud neck (3) is the first step in the transport termination process and this requires Vac17-Thr240 phosphorylation in the mother cell by an unknown kinase (K?) (2). Cla4 phosphorylates Vac17 at Ser222 (4) to activate the Dma1-dependent Vac17 ubiquitylation (5). Prior to its degradation by the proteasome (7), ubiquitylated Vac17 dissociates from the Myo2−Vac17 complex upon Yck3- and Vps41-dependent phosphorylation (6). (B) Cells were grown to log phase and incubated with FM4-64 dye for 1 h (pulse) to stain the vacuoles, and then transferred to medium without FM4-64. Cells were further grown to 4–5 h (chase) and imaged. The representative epifluorescence microscopy images show cells containing vacuoles stained with FM4-64 (red). To highlight the cell circumference (blue), brightfield images were collected in one plane and processed where necessary in a blue channel using Adobe Photoshop. (C) Quantification of vacuole inheritance in a cell population. Analysed were 100 budding cells (three independent experiments per strain) as shown in B. Category I, bright FM4-64 staining of vacuoles in mother cells and buds; category II, bright FM4-64 staining in mother cells but not in buds; category III, cells lack bright FM4-64 staining altogether. Statistical analysis was performed using two-way ANOVA test (Turkey's multiple comparisons). Significance is shown in comparison to WT cells. ***P=0.0005; ****P<0.0001; ns, not significant. Error bars indicate the standard error of the mean (+s.e.m.). (D) Representative epifluorescence microscopy images showing cells constitutively expressing the GFP-tagged vacuole membrane protein marker Vph1 (Vph1-GFP) that were stained with FM4-64 as described for B. In WT cells, vacuoles are labelled with both Vph1-GFP (green) and FM4-64 (red) indicating they have been successfully inherited. In *kin4Δfrk1Δ* cells, very little to no FM4-64 labelled vacuoles are observed in the bud; however, vacuoles in large buds are labelled with Vph1-GFP (green). These vacuoles had been formed *de novo* (white arrowheads). Yellow arrowheads indicate unbudded cells with Vph1-GFP but without FM4-64 signal. Cell circumferences are highlighted in blue. BF, brightfield images. Images are representative of three independent experiments. All scale bars: 5 μm.

In the budding yeast *Saccharomyces cerevisiae* (*S. cerevisiae*), organelle segregation during cell division is ensured by class V myosin motor-based transport to the emerging bud. The majority of organelles, including vacuoles (the yeast equivalent of mammalian lysosomes) and peroxisomes, are transported by Myo2 (Hill et al., 1996; Hoepfner et al., 2001). Organelle-specific receptors recruit Myo2 to initiate transport along the polarised actin cytoskeleton (Knoblach and Rachubinski, 2015). This directs the delivery of Myo2 cargoes to zones of cell growth, i.e. the growing bud or late in the cell cycle to the bud neck which is the site of cytokinesis (Bi and Park, 2012; Pruyne and Bretscher, 2000a,b; Wong and Weisman, 2021). However, vacuoles and peroxisomes are positioned away from the bud neck area as their transport is terminated prior to cytokinesis through detachment of Myo2 from the organelles (Yau et al., 2014). Organelle transport needs to be coordinated with the cell cycle; hence, transport is under both temporal and spatial control. The mechanisms controlling the transport of vacuoles have been studied intensively and many factors controlling the initiation and termination of vacuole transport have been identified (Fig. 1A) (Wong and Weisman, 2021).

Vac17 is the vacuole-specific Myo2 receptor and is essential for the transport of vacuoles to the emerging bud (Ishikawa et al., 2003). Cdk1-dependent phosphorylation of Myo2 and Vac17 is crucial for formation of the Myo2−Vac17 transport complex early in the cell cycle (Legesse-Miller et al., 2006; Peng and Weisman, 2008) (Fig. 1A). Vacuoles form segregation structures which are pulled from the tip by Myo2 motors into the growing bud whereas the rest of the vacuole is retained in the mother cell (Eves et al., 2012; Li et al., 2021). Vacuoles detach from Myo2 and are positioned near the centre of medium- to large-sized buds (Tang et al., 2003). Two parallel converging pathways control the detachment of vacuoles from Myo2. One pathway extracts Myo2 from vacuoles, and is dependent upon yeast casein kinase 3 (Yck3) and the vacuolar membrane protein Vps41 via an unknown mechanism (Wong et al., 2020). The second pathway is more characterised, and is regulated by an amino acid (aa) sequence rich in proline (P), glutamate (E), serine (S) and threonine (T), a so-called PEST motif, within Vac17 (residues 204−250 aa) (Tang et al., 2003). PEST motifs target proteins for rapid degradation (Rechsteiner and Rogers, 1996). The detachment process is initiated by phosphorylation of the Vac17 PEST motif at T240 in the mother cell by an unidentified kinase. In the bud neck, the E3-ubiquitin ligase Dma1 binds directly to Vac17 phosphorylated at T240 (Yau et al., 2014) (Fig. 1A). Subsequently, the p21-activated kinase (PAK) Cla4 phosphorylates Vac17 at S222 in the PEST motif, which activates Dma1 and results in ubiquitylation of Vac17 (Yau et al., 2017). This later phosphorylation event is spatially controlled as Cla4 localisation is restricted to the cortex of buds (Holly and Blumer, 1999; Peter et al., 1996). Mutation of S222 or T240 into alanine or deletion of the PEST motif (*PESTΔ*) interferes with spatially regulated Vac17 breakdown. In cells deficient in detachment of Myo2 from vacuoles, the vacuole remains attached to Myo2 till late in the cell cycle and follows Myo2 to the bud neck (Yau et al., 2014, 2017). Overexpression of Cla4 causes excessive degradation of Vac17 and a defect in vacuole transport to the bud. As this transport defect can be rescued by co-expression of the *vac17-PESTΔ* mutant (Bartholomew and Hardy, 2009), ectopic Cla4 activity may lead to aberrant Vac17 degradation, too, early in the cell cycle or at the wrong place, e.g. in the mother cell, thereby interfering with spatiotemporal control of Vac17 detachment and degradation. Dma2 and the PAK Ste20 provide minor contributions to vacuole-transport termination, and are partially redundant with Dma1 and Cla4, respectively (Yau et al., 2014, 2017). The current model of spatial and temporal regulation of vacuole transport is depicted in Fig. 1A. However, this model does not explain how degradation of Vac17 is prevented in small-budded cells.

Cla4 also plays important roles during the final stages of nuclear segregation and mitotic exit (Caydasi et al., 2017). Mitotic exit involves a cascade of events, also called the mitotic exit network (MEN) (Weiss, 2012). GTPase Tem1 is the master regulator of MEN and triggers mitotic exit only from the spindle pole body (SPB) on the elongated nucleus (spindle) that has entered the bud (Weiss, 2012) (Fig. S1A,B). Proper spindle alignment during anaphase is a prerequisite for successful SPB entry and subsequent Tem1 activation in the bud. If the spindle is misaligned during anaphase, Tem1 is kept inactive to prevent premature mitotic exit through the action of the spindle position checkpoint (SPOC). The SPOC kinase Kin4 activates the bipartite GTPase-activating protein (GAP) complex Bfa1−Bub2 that inhibits Tem1 GTPase activity in the mother cell (Caydasi and Pereira, 2012) (Fig. S1A,B). Regulation of MEN is explained by the zone model (Caydasi and Pereira, 2012; Falk et al., 2016) (Fig. S1C). This model proposes that, late in the cell cycle, large-budded cells are divided into two zones, i.e. a MEN inhibitory zone in the mother cell and a MEN-activating zone in the daughter cell. The inhibitory zone is controlled by activated Kin4 and mainly localised to the mother cell cortex at this phase of the cell cycle, whereas the activating zone is controlled by the Kin4 inhibitor Lte1, with the latter concentrated in the daughter cell. Lte1 also directly activates the MEN, and Cla4 is required for Lte1 activity in the daughter (Bertazzi et al., 2011; Caydasi et al., 2017) (Fig. S1B,C). In contrast to activation of MEN, Cla4-dependent breakdown of Vac17 in large buds occurs independently of Lte1 (Yau et al., 2017).

Although Kin4 is mainly localised to the mother cell cortex in large-budded cells, its localisation to other sites within the cell, including the cortex of small buds during S phase and early in G2 phase, suggest additional functions for Kin4 (Chan and Amon, 2009; Pereira and Schiebel, 2005). This is further supported by the observation that Elm1 kinase activates Kin4 by phosphorylation of Thr209 throughout the cell cycle and not just during anaphase when SPOC is active (Caydasi et al., 2010). Recently, we have shown that Kin4 and, to a lesser extent, its functional paralog Frk1 are required for peroxisome transport to the bud and, thus, for peroxisome inheritance (Ekal et al., 2023b). Kin4 and Frk1 maintain protein levels of the peroxisome-specific Myo2 receptor Inp2, but no mechanistic details have been reported to explain their contribution to peroxisome transport (Ekal et al., 2023b). In addition to peroxisome inheritance, vacuole inheritance is also affected in mutant cells lacking Kin4 and Frk1 (hereafter referred to as *kin4Δfrk1Δ* cells) (Ekal et al., 2023b).

In this current study, we demonstrate that the vacuole inheritance defect in *kin4Δfrk1Δ* cells is mainly due to increased Cla4-/Dma1-dependent Vac17 turnover. Further experiments investigating the interplay between Kin4/ Frk1 and Cla4/Dma1 support the idea that the zone model proposed for the regulation of mitotic exit could be extended to other spatiotemporal events, including polarised vacuole and peroxisome transport.

## RESULTS

### Kin4 and Frk1 are redundant in vacuole inheritance

*S. cerevisiae* Kin4 and its paralogue Frk1 are redundant in peroxisome transport to the bud (Ekal et al., 2023b). As *kin4Δfrk1Δ* cells are defective in vacuole inheritance (Ekal et al., 2023b), we tested whether Kin4 and Frk1 are also redundant in vacuole inheritance. Cells were pulse-chase labelled with the fluorescent lipophilic dye FM4-64 that accumulates in the vacuolar membrane (Vida and Emr, 1995) (for details, see Materials and Methods ‘Vacuolar staining with FM4-64’). This pulse-chase experiment allowed the tracking of pre-existing vacuolar membranes over time and, therefore, their inheritance. In virtually all wild-type (WT) and *frk1Δ* cells, including buds, vacuoles were stained with FM4-64, indicating efficient transfer of vacuoles from mother to daughter cells (Fig. 1B,C). In *vac17Δ* cells, transport of vacuoles to the bud is blocked. Consequently, ∼20% of mother cells contain brightly stained vacuoles, but their buds are empty and ∼80% of the cells lack bright FM4-64 staining altogether. In *kin4Δ* cells, a significant number of buds (11.7% ±1.4%) showed strongly decreased or undetectable levels of FM4-64 fluorescence. This was mainly observed in small-budded cells, implying a partial defect in vacuole transport. However, cells lacking FM4-64 staining were observed rarely (0.7%), with a frequency not significantly different from that of WT cells (Fig. 1B,C). This shows that *kin4Δ* cells still inherit vacuoles but that this process may be delayed. Upon additional deletion of Frk1 (*kin4Δfrk1Δ*) >40% of the cell population either failed to transport vacuoles or only transported a strongly reduced amount of FM4-64 labelled vacuoles from mother cell to bud. Many of the *kin4Δfrk1Δ* cells lacked brightly stained vacuoles all together (42.7%) (Fig. 1B,C). The synergistic effect of *KIN4* and *FRK1* gene deletion indicates that Kin4 function in vacuole transport is partially backed up by that of Frk1.

### *kin4Δfrk1Δ* cells fail to inherit vacuoles but can form them *de novo*

Vacuoles are essential for cell growth and progression through the cell cycle (Weisman et al., 1990). In cells that fail to transport vacuoles to the bud, such as in *vac17Δ* cells, new vacuoles are formed in the bud (Jin and Weisman, 2015). Since vacuole inheritance is affected in *kin4Δfrk1Δ* cells, *de novo* formation of vacuoles was analysed in *kin4Δfrk1Δ* and compared to WT and *vac17Δ* cells. Cells constitutively expressing the GFP-tagged version of the vacuole membrane marker protein Vph1 (Vph1-GFP) were pulse-chase labelled with FM4-64 before being imaged. Vacuoles in the mother cell and bud of WT cells were labelled with both FM4-64 and Vph1-GFP (Fig. 1D) implying proper vacuole segregation during cell growth and division in line with previous observations (Jin and Weisman, 2015). In contrast, in some *kin4Δfrk1Δ* cells, vacuoles only showed Vph1-GFP but not FM4-64 fluorescence. Moreover, in many *kin4Δfrk1Δ* budding cells, buds contained vacuoles labelled with Vph1-GFP only (Fig. 1D). Similar observations were made in *vac17Δ* cells that were used as a control for *de novo* vacuole formation. We, therefore, conclude that many of the *kin4Δfrk1Δ* mother cells fail to pass on their vacuoles to their daughters and that, consequently, vacuoles were formed *de novo* in their buds (Fig. 1C,D).

### Kin4 and Frk1 affect Vac17 ubiquitylation and turnover

**Fig. 2.**
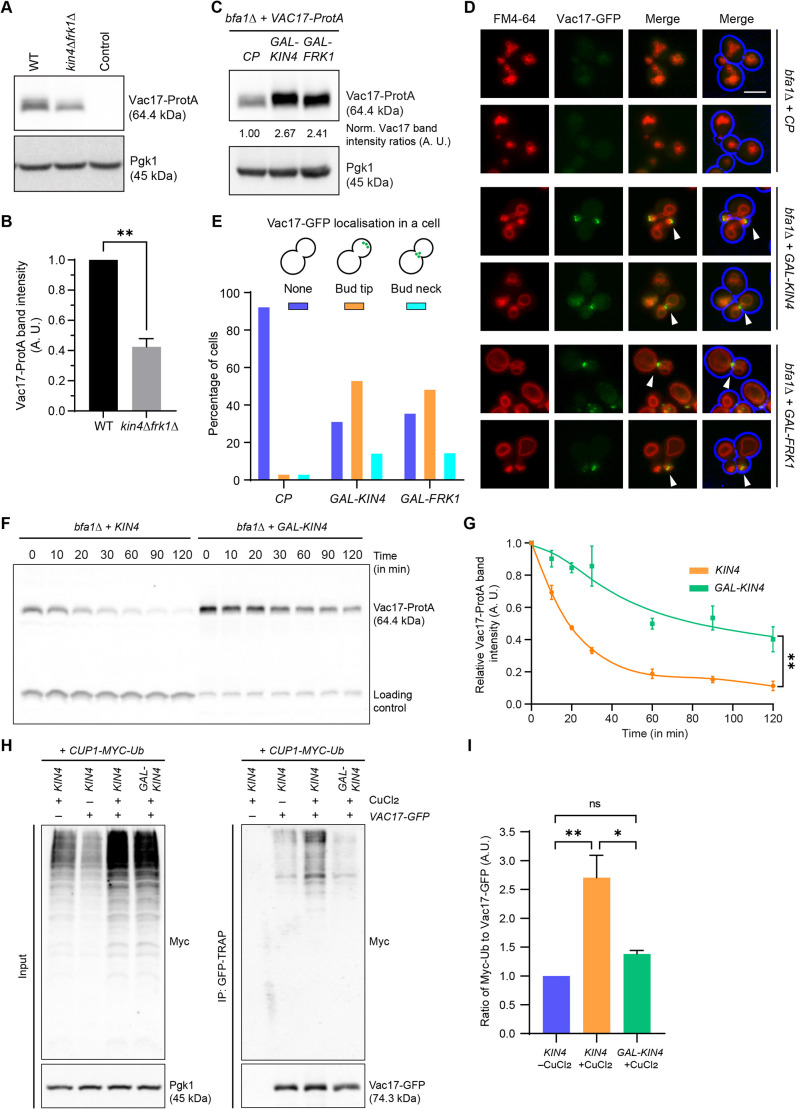
**Kin4 and Frk1 are required to maintain the steady-state levels of Vac17 protein.** (A) Cell extracts were analysed by western blotting. Compared to WT cells, steady-state protein levels of Vac17-ProtA are strongly reduced in *kin4Δfrk1Δ* cells. Control indicates cells without Vac17-ProtA expression. Pgk1 was used as a loading control. (B) Quantification of Vac17-ProtA protein levels in WT and *kin4Δfrk1Δ* cells from three independent experiments. Vac17-ProtA band intensity was normalised against Pgk1 band intensity. Normalised Vac17-ProtA signals in WT cells were set to 1 arbitrary unit (A.U.). Statistical analysis was performed using a two-tailed paired *t*-test. **P=0.0044. Error bars indicate the standard error of the mean (+s.e.m.). (C) Western blots, showing that Vac17-ProtA levels are elevated upon Kin4 and Frk1 overexpression in *bfa1Δ* cells. *bfa1Δ* cells expressing Vac17-ProtA were transformed with either *GAL-KIN4* or *GAL-FRK1* or control plasmid (*CP*) and grown in a minimal medium containing galactose as a sole carbon source (YM+Gal) to induce Kin4 and Frk1 overexpression. Blots shown are representative of three independent experiments. (D) Location of Vac17-GFP and vacuole positioning upon Kin4/Frk1 overexpression was analysed by epifluorescence microscopy. Vac17-GFP expression (green) was from a centromeric plasmid under its endogenous promoter. Arrowheads indicate mispositioning of Vac17-GFP together with vacuoles at the bud neck and tip. Cell circumferences are highlighted in blue. Scale bar: 5 μm. (E) Quantification of Vac17-GFP localisation for strains in D. A minimum of 133 cells were analysed for each strain. Colours indicate categories with specific Vac17-GFP localisation. Blue, no clear localisation; orange, bud tip localisation; cyan, bud neck localisation. (F) Representative western blot. Kin4 overexpression stabilises Vac17 by reducing its turnover. Kin4 and Vac17-ProtA protein expression was as described for C. Cells were grown in YM+Gal medium and this was followed by cycloheximide (CHX) treatment. Cells were harvested at indicated time points and cell extracts analysed. (G) Quantification of Vac17-ProtA levels (as shown for F) from three independent experiments. Vac17-ProtA band intensities were normalised to that of the loading control band and the value at time point 0 min was set to 1 A.U. Statistical significance was performed using two-tailed paired *t*-test. **P=0.0029. Error bars indicate ±s.e.m. (H,I) Kin4 overexpression reduces Vac17 ubiquitylation. Cells were co-transformed with plasmids encoding Myc-tagged ubiquitin (Myc-Ub) and Vac17-GFP. Myc-Ub and Vac17-GFP were expressed under the control of a copper-inducible promoter (*CUP1*) and a constitutive *ADH1* promoter, respectively. (H) Vac17-GFP was immunoprecipitated using GFP-TRAP nanobody resins and analysed by western blotting. To detect ubiquitylation, Vac17-GFP IP samples were used for immunoblotting with anti-Myc antibody. Pgk1 was used as a loading control. (I) Quantification data for ubiquitylation from three independent experiments as shown in H. Statistical analysis was carried out using a one-way ANOVA test (Holm–Šidák's multiple comparison test). *P=0.0122, **P=0054. ns, not significant. Error bars indicate +s.e.m.

The inheritance defect for vacuoles in *kin4Δfrk1Δ* cells resembles the defect observed in mutants that lack Vac17. Western blot analysis revealed that protein levels of Vac17 tagged with the *Staphylococcus aureus* protein A (ProtA) were significantly reduced in *kin4Δfrk1Δ* cells compared to in WT cells (Fig. 2A,B; Fig. S8). Next, we tested whether Kin4 and Frk1 overexpression would lead to increased levels of Vac17. As *KIN4* and *FRK1* overexpression is lethal for WT cells by blocking mitotic exit via Bfa1 (Ekal et al., 2023b; Maekawa et al., 2007), *BFA1*-knockout (*bfa1Δ*) cells expressing Vac17-ProtA were transformed with plasmids containing either *KIN4* or *FRK1* under control of a strong galactose-inducible promoter (*pGAL-KIN4, pGAL-FRK1*). Protein extracts from cells grown on galactose were analysed by western blotting. Vac17-ProtA levels were elevated in cells overexpressing either *KIN4* or *FRK1* compared to control cells (Fig. 2C). We conclude that Kin4 and Frk1 affect Vac17 steady-state levels.

To study the effect of *KIN4* or *FRK1* overexpression on the positioning of vacuoles and Vac17-GFP, *bfa1Δ* cells were transformed with a Vac17-GFP expression plasmid under the control of its endogenous promoter and vacuoles were visualised by FM4-64 staining. Overexpression of either *KIN4* or *FRK1* increased the Vac17-GFP signal (Fig. 2D,E). Furthermore, Vac17-GFP was not visible in the majority (92.8%) of *bfa1Δ* cells transformed with a control plasmid (*bfa1Δ+CP* cells) but was visible in those overexpressing *KIN4* or *FRK1*, where it was located either at the bud tip in small-budded cells or at the bud neck in large-budded cells (Fig. 2D,E). In large-budded control cells, vacuoles were positioned away from the bud neck, whereas in cells overexpressing *KIN4* or *FRK1*, vacuoles frequently localised to the bud neck where they colocalise with Vac17-GFP (Fig. 2D,E). This phenotype is reminiscent of mutants in which vacuoles – owing to the continued association of vacuoles with Myo2 − are not released in the bud but are transported back to the bud neck, (Wong et al., 2020; Yau et al., 2014, 2017) (Fig. S2).

To test whether the increase in the level of Vac17 observed upon overexpression of *KIN4* is a consequence of an increase in Vac17 stability, we performed a cycloheximide (CHX) chase assay. CHX blocks protein synthesis and, thereby, permits analysis of the degradation kinetics of steady-state protein levels. A control experiment in WT cells showed a clear and strong reduction of Vac17-ProtA levels upon treatment with CHX; however, no reduction in Vac17-ProtA levels was observed in cells not treated with CHX (Fig. S3). To study Vac17 turnover upon *KIN4* overexpression, *bfa1Δ* cells transformed with the plasmid *pGAL-KIN4* or a negative control plasmid were grown in medium containing galactose as a carbon source (YM+Gal) to induce *GAL-KIN4* expression for 6 h and then treated with CHX. Vac17-ProtA turnover was significantly reduced in cells that overexpressed *KIN4* compared to those that did not (Fig. 2F,G). Next, we used a well-established *in vivo* ubiquitylation assay for Vac17 (Yau et al., 2014, 2017). Briefly, in cells expressing Vac17-GFP, expression of Myc epitope-tagged ubiquitin (Myc-Ub) was induced by addition of CuCl_2_. Subsequently, Vac17-GFP was precipitated by using GFP-TRAP and precipitates were analysed by immunoblotting for the presence of Myc-Ub to reveal relative levels of Vac17 ubiquitylation *in vivo*. We observed a clear ubiquitylation pattern in precipitates from cells expressing Vac17-GFP, which was absent in cells without Vac17-GFP expression (Fig. 2H). Upon *KIN4* overexpression there was a significant decrease in Myc-ubiquitylated Vac17-GFP compared to those cells not overexpressing *KIN4* (Fig. 2H,I). The above results show that changes in Kin4 protein levels affect Vac17 ubiquitylation and, thus, its turnover.

### Vacuole inheritance requires Kin4 kinase activity

**Fig. 3.**
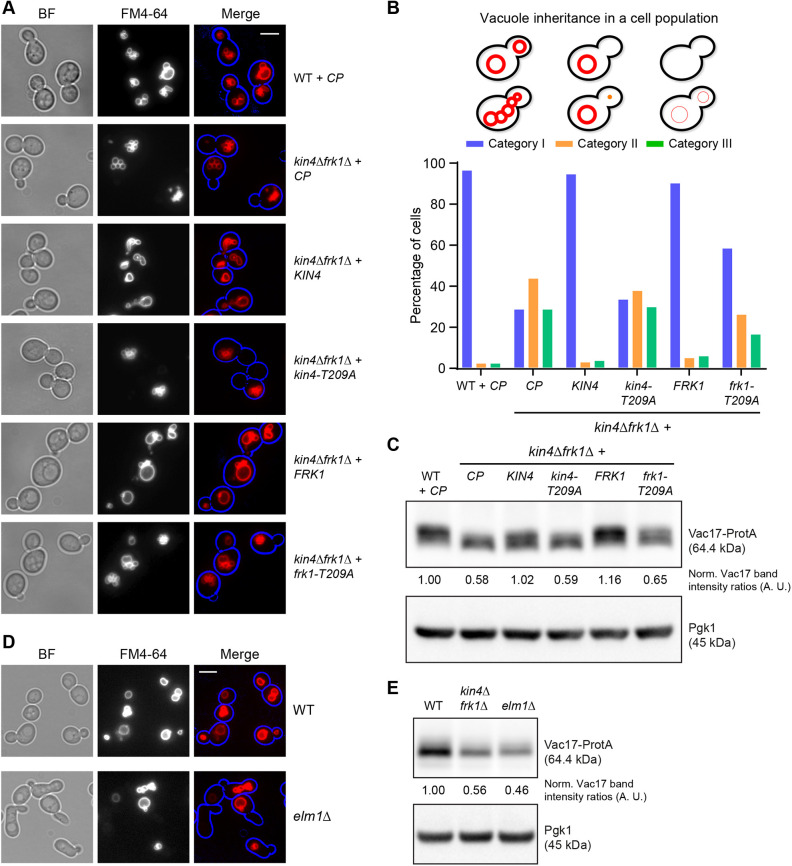
**Kin4 kinase activity is required for vacuole inheritance.** (A) Expression of Kin4 or Frk1 but not *kin4-T209A* or *frk1-T209A*, respectively, in *kin4Δfrk1Δ* cells restored vacuole inheritance comparable to WT cells. Cells were stained with FM4-64 (red) and imaged. *CP*, control plasmid. To highlight the cell circumference (blue), brightfield (BF) images were collected in one plane and processed where necessary in a blue channel using Adobe Photoshop. Scale bar: 5 µm. (B) Quantification of vacuole inheritance in a cell population of strains as shown in A. A minimum of 102 cells per strain were visually inspected. Category I, bright FM4-64 staining of vacuoles in mother cells and buds; category II, bright FM4-64 staining in mother cells but not in buds; category III, cells lack bright FM4-64 staining altogether. (C) Steady-state protein levels of Vac17-ProtA were analysed for the strains as shown in A. Cell extracts were analysed by western blotting. Blots shown are representative of two independent experiments. (D) Cells lacking *ELM1* are defective in vacuole inheritance. WT and *elm1Δ* cells were tested for vacuole inheritance by FM4-64 (red) staining and followed by imaging. Cell circumference is highlighted in blue. Scale bar: 5 μm. Images are representative of two independent experiments. (E) Cell extracts from WT, *kin4Δfrk1Δ* and *elm1Δ* cells expressing Vac17-ProtA analysed by western blotting. Blots shown are representative of two independent experiments. Vac17-ProtA expression was under the control of the *VAC17* promoter in C,E. Pgk1 was used as a loading control. Vac17-ProtA band intensity was normalised against Pgk1 band intensity. Normalised Vac17-ProtA signals in WT cells were set to 1 arbitrary unit (A.U.).

Phosphorylation of aa residue T209 within the Kin4 kinase activation loop by Elm1 is crucial for Kin4 function in SPOC as well as for peroxisome transport (Caydasi et al., 2010; Ekal et al., 2023b; Moore et al., 2010). To test whether Kin4 kinase activity is also required for vacuole inheritance, *kin4Δfrk1Δ* cells expressing either wild-type *KIN4* or *kin4-T209A* were pulse-chased with FM4-64 and imaged. Vacuole inheritance was restored by wild-type Kin4 but not by *kin4-T209A*. The activation loop of Kin4 and Frk1 are identical in amino acid sequence including the T209 residue (Ekal et al., 2023b). Moreover, as shown previously, toxicity caused by *FRK1* overexpression can be rescued by the deletion of the *ELM1* gene (Ekal et al., 2023b). This tempted us to postulate that Frk1 is also a potential substrate for Elm1. Therefore, Frk1 was also tested in this assay. Indeed, expression of wild-type Frk1 restored vacuole inheritance whereas *frk1-T209A* only partially restored inheritance, illustrating the importance of T209 for Frk1 function (Fig. 3A,B). Restoration of inheritance is accompanied by an increase of Vac17-ProtA levels to almost WT levels (Fig. 3C). Moreover, Vac17-ProtA is phosphorylated on many sites (Peng and Weisman, 2008; Yau et al., 2014; Zhou et al., 2021). During SDS-PAGE, Vac17-ProtA from WT samples migrates differently compared to that derived from *kin4Δfrk1Δ* cells. Reintroduction of *KIN4* or *FRK1* in *kin4Δfrk1Δ* cells, changes the Vac17-ProtA migration pattern whereas the introduction of *kin4-T209A* resembled that of *kin4Δfrk1Δ* cells. The introduction of *frk1-T209A* resulted in an intermediate migration pattern (Fig. 3C). These observations suggest that Vac17-ProtA is differentially modified depending on Kin4 and Frk1 activity. However, whether the change in migration pattern is a result of direct phosphorylation by Kin4 or Frk1 is unknown. So far, experiments addressing this, have failed to identify specific Kin4 phosphorylation sites in Vac17. Furthermore, many buds of *elm1Δ* cells lacked FM4-64 pulse-labelled vacuoles, implying a vacuole inheritance defect. Not many budding mother cells were found without any vacuoles (category III), but this could be a consequence of the slow progression of the cell cycle through the G2/M phase in *elm1Δ* cells (Moriya and Isono, 1999) (Fig. 3D; Fig. S4A,B). Moreover, western blot analysis showed that Vac17-ProtA levels were strongly reduced in *elm1Δ* cells and were comparable to those in *kin4Δfrk1Δ* cells (Fig. 3E). Combined, all these observations strongly suggest that Elm1 activates Kin4 as well as, albeit to a lesser extent, Frk1 by phosphorylation of T209 in its activation loops and that this is required for vacuole inheritance.

### Kin4 function in vacuole inheritance is independent of its role in SPOC

During SPOC, the bipartite GTPase complex Bfa1−Bub2 is activated by phosphorylation of the Bfa1 subunit through Kin4 (Maekawa et al., 2007). In addition, the PP2A phosphatase subunit Rts1 regulates Kin4 localisation to spindle pole bodies; this is required for Kin4 function at the SPOC (Chan and Amon, 2009). We analysed vacuole inheritance in cells lacking *BFA1*, *BUB2* or *RTS1* (*bfa1Δ*, *bub2Δ* or *rts1Δ* cells, respectively). None of the mutants showed a strong defect in vacuole inheritance (Fig. S4A,B). Kar9 is a Myo2-receptor for astral microtubule transport and plays an important role in maintaining the spindle alignment along the cell polarity axis (Beach et al., 2000; Miller and Rose, 1998). Thus deletion of *KAR9* (*kar9Δ*) leads to spindle misalignment in many cells, which results in activation of SPOC (Pereira et al., 2000). WT, *kar9Δ* and *kin4Δfrk1Δ* cultures that express GFP-tagged tubulin1 (GFP-Tub1), a marker for the mitotic spindle, were grown to exponential growth phase and stained with FM4-64, before being analysed by epifluorescence microscopy. The large-budded *kin4Δfrk1Δ* cells defective in vacuole transport do not show defects in spindle alignment as those observed in *kar9Δ* cells (Fig. S4C). Furthermore, in contrast to Vac17 and Inp2, there is no reduction in Kar9 protein levels in *kin4Δfrk1Δ* cells compared to in those in WT cells (Fig. 2A,B; Fig. S4D) (Ekal et al., 2023b). Taken together, we conclude that SPOC is not required for vacuole inheritance and, therefore, the role of Kin4 in vacuole transport is independent of its role in SPOC. These results are in line with previous observations, where the function of Kin4 in peroxisome transport has been shown to be independent of its function in SPOC (Ekal et al., 2023b).

### Vac17 interacts with Myo2 in *kin4Δfrk1Δ* cells

**Fig. 4.**
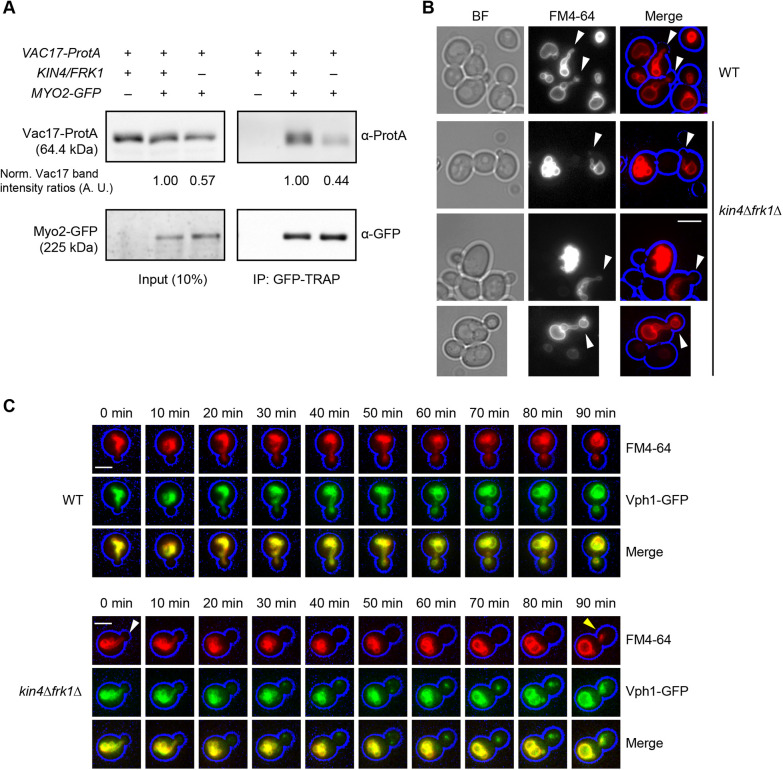
**Vac17 interacts with Myo2 in *kin4Δfrk1Δ* cells.** (A) Vac17 and Myo2 interaction was analysed by co-immunoprecipitation (IP) assay for which Myo2 was tagged with GFP in the genome and Vac17-ProtA was expressed from a plasmid under the control of its promoter. Myo2-GFP was affinity purified using GFP-TRAP nanobody beads. Inputs and IP samples were analysed using anti-GFP and anti-ProtA antibodies. Immunoprecipitated Vac17-ProtA was quantified from two independent experiments. Vac17-ProtA signals in WT cells were set to 1 arbitrary unit (A.U.) and then normalised with Myo2-GFP in input and GFP-TRAP blots, separately. (B) Representative epifluorescence microscopy images showing the vacuole segregation structures (indicated by arrowheads) observed in some *kin4Δfrk1Δ* cells, mainly in small-budded cells. WT and *kin4Δfrk1Δ* cells were pulse chased with FM4-64 (red) and imaged. Cell circumference is highlighted in blue. BF, brightfield images. Images are representative of three independent experiments. (C) Time-lapse microscopy analysis of vacuole movement. Shown are representative fluorescence images of WT and *kin4Δfrk1Δ* cells expressing Vph1-GFP (green) under the control of the *VPH1* promoter. Cells had been transiently stained with FM4-64 (red) and images were captured at 10 min time intervals. Only a small fraction of the vacuole is passed on from mother to daughter cell between 80 and 90 min of this image series (yellow arrowhead). Kin4 and Frk1 are not required for segregation structure (white arrowhead) formation but for the maintenance of these structures. To highlight the cell circumference (blue), brightfield images were collected in one plane and processed where necessary in a blue channel using Adobe Photoshop. Images are representative of three independent experiments. All scale bars: 5 μm.

The defect in vacuole inheritance observed in *kin4Δfrk1Δ* cells is caused by a decrease in organelle transport. Decreased transport could be caused by failure to assemble transport complexes to initiate transport or to maintain transport. Previous studies have shown that, in cells with defective recruitment of Myo2 by Vac17, Vac17 levels increase as vacuoles and Vac17 associated with them remain in the mother cell, and are kept away from the bud-restricted Vac17 degradation mechanism (Eves et al., 2012; Tang et al., 2003). In *kin4Δfrk1Δ* cells, Vac17 levels are reduced even though there is an inheritance defect, and most vacuoles remain in the mother cell. This suggests that it is not the assembly of the Myo2−Vac17 complex that is affected. Indeed, co-immunoprecipitation experiments revealed that Vac17-ProtA binds Myo2 in *kin4Δfrk1Δ* cells (Fig. 4A). However, the complex is present at lower levels and this may lead to inefficient transport to the bud. Typically, vacuoles form a segregation structure during the early stages of transport and the structures were observed in 29.3% (64 out of 218) of cells with small- to medium-sized buds (Fig. 4B). In *kin4Δfrk1Δ* cells, segregation structures were only observed in 5.3% (10 out of 187) of the cells (Fig. 4B)**.** Time-lapse imaging of FM4-64 pulse chased cells confirmed that segregation structures are formed in *kin4Δfrk1Δ* cells but that, in contrast to in WT cells, these structures failed to be maintained over a long period of time (Fig. 4C). Instead, as shown in the example of the budding *kin4Δfrk1Δ* cell, the segregation structure observed at t=0 and t=10 disappeared and vacuoles are being synthesised *de novo* in the bud (Vph1-GFP stained only) between 30 min and 80 min (Fig. 4C). Subsequently, some transport of pre-existing vacuolar membrane (stained with FM4-64) occurred between 80 and 90 min (Fig. 4C), although we did not capture the transient segregation structure. Thus, while in WT cells the segregation structure lasted >1 h, the structure was short-lived in *kin4Δfrk1Δ* cells. We conclude that *kin4Δfrk1Δ* cells can assemble Myo2−Vac17 transport complexes but fail to maintain the continued vacuolar segregation.

### Kin4 and Frk1 affect Vac17 levels in mother cells

Since Kin4 activity is restricted to the mother cell during SPOC in medium- to large-budded cells (Caydasi and Pereira, 2012) (Fig. S1B,C), we tested whether stabilisation of Vac17 by Kin4 also occurs in the mother cell. We made use of the previous observation that − in mutants affecting the Vac17-Myo2 interaction, including *myo2-D1297N* − Vac17 remains in the mother cell at elevated levels (Eves et al., 2012; Tang et al., 2003). In line with previous observations, elevated levels of Vac17-ProtA were detected in *myo2-D1297N* cells compared with those in cells expressing wild-type *MYO2* (Fig. S5A). Interestingly, *myo2-D1297N* cells lacking *KIN4* and *FRK1* failed to maintain these elevated levels of Vac17 (Fig. S5B). A CHX chase assay revealed an increase in Vac17 turnover in *myo2-D1297N* cells lacking *KIN4* and *FRK1*, compared to in *myo2-D1297N* cells with *KIN4* and *FRK1* present (Fig. S5C,D).

Next, we sought to test whether the above observations are also reproducible with Vac17 mutants that fail to interact with Myo2. The structure of Vac17 (112−157 aa) in complex with the Myo2-cargo-binding domain (CBD) had been predicted and the Myo2 interaction site (MIS) in Vac17 had been narrowed down to aa residues 131−145 (Liu et al., 2022). Moreover, point mutations within three Vac17-MIS residues (i.e. R135, K138 and R142) reduce interaction with Myo2-CBD *in vitro* (Liu et al., 2022) (Fig. S6A). To further corroborate these results, we performed yeast two hybrid (Y2H) assays to study the Vac17 and Myo2-CBD interaction *in vivo*, as described previously by Eves et al. (2012). Here, we generated single- and double-point mutations in the above three Vac17 residues, and tested them for interaction with Myo2-CBD using growth in the absence of adenine and histidine as readout (Fig. S6B). A control plasmid without Vac17 (BD) did not show any growth; however, expression of wild-type Vac17 showed proper growth, suggesting strong and specific interaction with Myo2-CBD (*MYO2*). Interestingly, *vac17-R135E* showed interaction with Myo2-CBD similar to wild-type Vac17, whereas *vac17*-*K138D* and *R142E* showed reduced interaction (Fig. S6B). However, the expression of three double mutants *vac17-R135E,K138D*, *vac17-R135E,R142E* or *vac17-K138D,R142E* did not restore cell growth at all and hinted towards a strongly reduced interaction with Myo2 (Fig. S6B). Subsequently, Myo2-CBD mutants (i.e. *myo2-E1293 K*, *myo2-D1297N*, *myo2-N1304D*) known to interfere with binding of Vac17 were tested in a two-hybrid assay. Here, *myo2-D1296N* was used as a control that still interacts with Vac17 (Fig. S6B). Interestingly, *vac17-R142E* and *vac17-R135E,R142E* restored interaction with *myo2-E1293 K* but not *myo2-D1297N* or *myo2-N1304D*. The restoration of an interaction between two charge reversal mutants that, on their own, are each affected in their binding to WT partners is strong evidence that these residues interact *in vivo* and support the structural model in Fig. S6A). *vac17Δ* cells expressing *vac17-R135E* mutant restored vacuole transport comparable to that of wild-type, whereas expression of *vac17-K138D* and *vac17-R142E* mutants only partially restored vacuole inheritance (Fig. S6C,D). Expression of *vac17-R135E,K138D*, *vac17-R135E,R142E* or *vac17-K138D,R142E* double mutants showed a strong defect in vacuole transport (Fig. S6C,D). Western blot analysis revealed that protein levels of all single and double mutants were increased compared to WT Vac17 levels − except for the *vac17-R135E* mutant, which did not affect vacuole inheritance (Fig. S6E). These results corroborated the Y2H analysis and validated the function of the previously identified Vac17-MIS *in vivo*. We selected *vac17-R135E, K138D* MIS mutant for further studies.

**Fig. 5.**
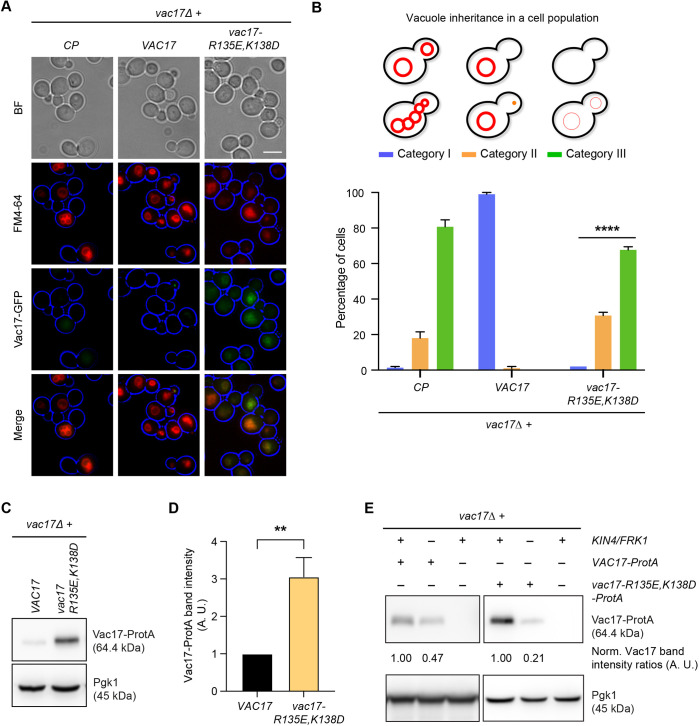
**Kin4 and Frk1 are required to maintain elevated Vac17 levels in mother cells.** (A) Representative epifluorescence microscopy images of exponentially growing *vac17Δ* cells expressing C-terminally GFP-tagged Vac17 WT (*VAC17*) and *R135E,K138D* double (MIS) mutant (*vac17-R135E,K138D*). Cells had been pulse-chased with FM4-64 (red). Brightfield (BF) images from one or two focal planes were processed to highlight the cell circumference (blue). Vac17-GFP is shown in green. Scale bar: 5 µm. *CP*, control plasmid. (B) Quantification of vacuole positioning in 100 cells from three independent experiments for strains as shown in A. Category I, bright FM4-64 staining of vacuoles in mother cells and buds; category II, bright FM4-64 staining in mother cells but not in buds; category III, cells lack bright FM4-64 staining altogether. Statistical significance was determined using a two-way ANOVA test (Tukey's multiple comparisons). Statistical significance is shown in comparison to (*vac17Δ+VAC17*) cells. ****P<0.0001. Error bars indicate the standard error of the mean (+s.e.m.). (C) Cell extracts from *vac17Δ* cells expressing ProtA-tagged Vac17 or Vac17-MIS mutant were analysed by western blotting. (D) Intensity values for Vac17-ProtA bands were normalised against unsaturated Pgk1 bands from three independent experiments and were plotted. Normalised ProtA signals in WT cells were set to 1 arbitrary unit (A.U.). Statistical significance was determined using a two-tailed paired *t*-test. **P<0.0022. Error bar indicates +s.e.m. (E) Cells lacking *KIN4* and *FRK1* fail to maintain elevated levels of Vac17-MIS mutant. ProtA-tagged Vac17 WT and MIS mutant were expressed in *vac17Δ* and *kin4Δfrk1Δvac17Δ* cells. Cell extracts were analysed by immunoblotting using anti-ProtA antibody. Blots shown are representative of three independent experiments. Vac17-GFP/ProtA expression was carried out under the control of the *VAC17* promoter in experiments shown in A, C and E.

To study Vac17 localisation, wild-type Vac17-GFP or *vac17-R135E,K138D-GFP* were expressed and visualised in *vac17Δ* cells. Expression of *vac17-R135E,K138D-GFP* failed to restore vacuole inheritance in *vac17Δ* cells and vacuoles in mother cells were decorated by GFP-signals. In contrast, Vac17-GFP restored inheritance of vacuoles and was hardly detectable by epifluorescence microscopy, although weak Vac17-GFP puncta were present in some small buds (Fig. 5A,B), in agreement with previous reports (Yau et al., 2014, 2017). Western blot analysis revealed elevated levels of *vac17-R135E,K138D-ProtA* compared to Vac17-ProtA (Fig. 5C,D; Fig. S6E). This is in line with earlier observations in cells expressing *myo2-D1297N* (Yau et al., 2014) (Fig. S5A). Next, we analysed whether the elevated protein levels of *vac17-R135E,K138D* are affected by deletion of *KIN4* and *FRK1*. Indeed, *vac17-R135E,K138D* protein levels were strongly reduced in the absence of both Kin4 and Frk1 (Fig. 5E). Therefore, as *vac17-R135E,K138D* is associated with the vacuole in the mother cell (Fig. 5A), we concluded that Kin4 and Frk1 protect Vac17 from degradation in mother cells.

### Blockage of Cla4-/Dma1-dependent Vac17 degradation rescues the vacuole transport defect in *kin4Δfrk1Δ* cells

**Fig. 6.**
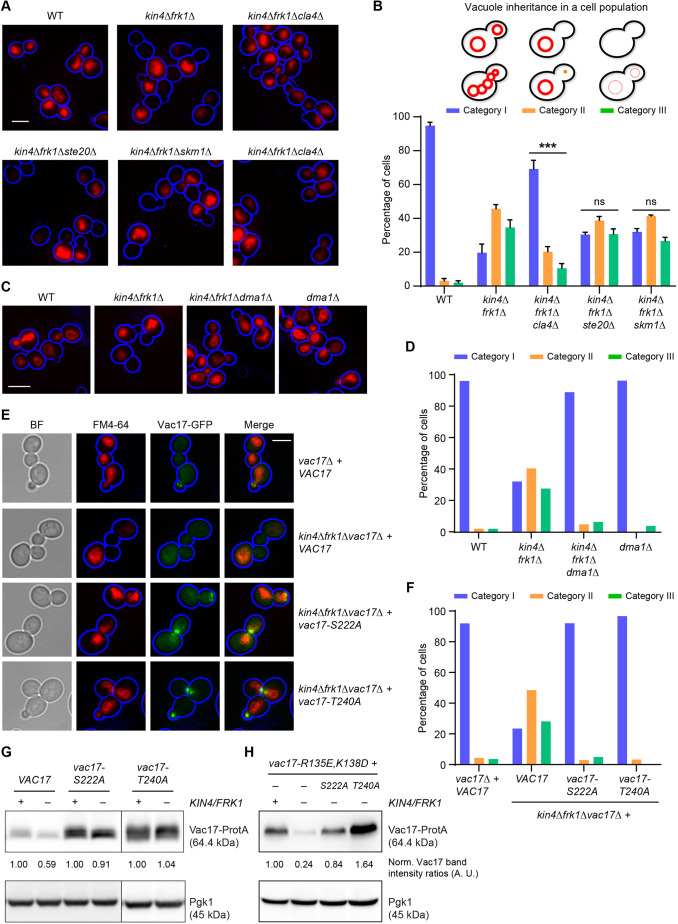
**A block in Cla4-/Dma1-dependent Vac17 degradation rescues vacuole transport defect in *kin4Δfrk1Δ* cells.** (A) Representative epifluorescence microscopy images of cells from the indicated strains pulse-chased with FM4-64 (red). Cell circumference is highlighted in blue. (B) Quantification of vacuole inheritance in a cell population for the strains analysed in A. Per strain, a minimum of 108 cells were analysed from three independent experiments. Category I, bright FM4-64 staining of vacuoles in mother cells and buds; category II, bright FM4-64 staining in mother cells but not in buds; category III, cells lack bright FM4-64 staining altogether. Statistical analysis was performed using two-way ANOVA (Turkey's multiple comparisons) test. Significance is shown in comparison to WT cells. ***P=0.0002, ns: not significant. Error bars indicate the standard error of the mean (+s.e.m.). (C) Additional *DMA1* deletion in *kin4Δfrk1Δ* cells rescues vacuole inheritance defects. Representative epifluorescence microscopy images from the indicated strains stained with FM4-64 (red). Cell circumference is highlighted in blue. (D) Quantification of vacuole inheritance in a cell population where ≥102 cells per strain in C were visually inspected. Categories as in B. (E) Expression of *S222A* or *T240A* Vac17-GFP mutants rescued vacuole inheritance defects in *kin4Δfrk1Δ* cells. Representative epifluorescence microscopy images from the indicated strains stained with FM4-64 (red). Vac17-GFP is shown in green. Cell circumference is highlighted in blue. BF, brightfield images. (F) A minimum of 90 cells per strain (as shown in E) were visually inspected. Categories as in B. (G) Expression of *vac17-S222A* or *vac17-T240A* mutants restored steady-state protein levels in *kin4Δfrk1Δ* cells. (H) Additional S222A or T240A mutations in Vac17 restored protein levels of *vac17-R135E,K138D* (MIS mutant). Cells were transformed with plasmids encoding *VAC17-ProtA* versions (wild-type or *R135E,K138D* or *R135E,K138D,S222A* or *R135E,K138D,T240A*) under the control of its native promoter. Cell extracts were analysed by western blotting. Plasmids containing GFP/ProtA tagged Vac17 versions were expressed under the control of the *VAC17* promoter in experiments shown in E, G and H). Pgk1 was used as a loading control in G and H. Vac17-ProtA band intensity was normalised against that of Pgk1. Normalised Vac17-ProtA signals in WT cells were set to 1 arbitrary unit (A.U.). Blots shown in G and H are representative of three independent experiments. All scale bars: 5 µm.

Cla4/Dma1 and Yck3/ Vps41 have each been implicated in the spatially controlled breakdown of Vac17 (Wong et al., 2020; Yau et al., 2017). We, therefore, hypothesised that the defect in *kin4Δfrk1Δ* cells is a consequence of misregulation of one of these processes. To test this, *kin4Δfrk1Δ* cells lacking either *CLA4* or *STE20* were generated and vacuole inheritance was analysed in these strains. As the *S. cerevisiae* genome encodes the Cla4 paralog Skm1, we also included the *SKM1* gene in our analysis. Deletion of *CLA4* significantly restored transport of vacuoles to the bud in *kin4Δfrk1Δ* cells, whereas deletion of either *STE20* or *SKM1* failed to do so (Fig. 6A,B). Deletion of *DMA1* in *kin4Δfrk1Δ* cells rescued the inheritance defect to almost WT level (Fig. 6C,D) but deletion of either *YCK3* or *VPS41* in *kin4Δfrk1Δ* cells did not. In contrast, *kin4Δfrk1Δyck3Δ* and *kin4Δfrk1Δvps41Δ* cells showed increased (additive) defects in vacuole inheritance compared to that in *kin4Δfrk1Δ* cells (Fig. S7A,B). Moreover, in contrast to those in *yck3Δ* and vp*s41Δ* cells, observed Vac17-GFP levels were less at the bud tip/bud neck in *kin4Δfrk1Δyck3Δ* and *kin4Δfrk1Δvps41Δ* cells (Fig. S7C). Notice that, in line with earlier reports, *yck3Δ* and *vps41Δ* cells show a weak inheritance defect within some strain backgrounds, in addition to increased levels of Vac17 and a transport termination defect (Fig. S7B,D) (LaGrassa and Ungermann, 2005; Wong et al., 2020). These results indicate that Cla4 and Dma1 but not Yck3 and Vps41 function antagonistically to Kin4 and Frk1.

Cla4 and Dma1 act in multiple cellular pathways, including septin dynamics, which are crucial for bud morphogenesis and recruitment of Elm1 at the bud neck during SPOC (Merlini et al., 2012; Versele and Thorner, 2004). Hence, to rule out the possibility of a pleiotropic effect caused by deletion of *CLA4* and *DMA1*, we analysed the Vac17 point mutants *S222A* and *T240A*, which are blocked in Cla4- and Dma1-dependent ubiquitylation, respectively (Yau et al., 2014, 2017). GFP-tagged *VAC17*, *vac17-S222A* and *vac17-T240A* were expressed in *kin4Δfrk1Δvac17Δ* cells, and vacuole inheritance was analysed using FM4-64 pulse-chase labelling. As expected, *kin4Δfrk1Δvac17Δ* cells expressing Vac17-GFP showed a severe defect in vacuole inheritance but expression of either *vac17-S222A-GFP* or *vac17-T240A-GFP* restored vacuole inheritance to WT levels (Fig. 6E,F). In addition, *vac17-T240A-GFP* and *vac17-S222A-GFP* signals were strongly increased and, in many cells, vacuoles were found to position inappropriately at the bud neck of large-budded cells, indicating a failure of timely *vac17-T240A-GFP* and *vac17-S222A-GFP* breakdown, respectively, as well as vacuole detachment from Myo2 (Fig. 6E). Moreover, Vac17-ProtA levels in S222A and T240A mutant cells were not affected by the absence of *FRK1* and *KIN4* (Fig. 6G). These results support a model, in which the decreased levels of Vac17 observed in *kin4Δfrk1Δ* cells are the result of Cla4-/Dma1-dependent Vac17 breakdown.

### Kin4 and Frk1 can prevent Cla4-/Dma1-dependent premature Vac17 breakdown in mother cells

Since vacuole inheritance and Vac17 protein levels in *kin4Δfrk1Δ* cells are restored upon inhibition of Cla4-/Dma1-dependent turnover of Vac17, we hypothesised that Cla4 and Dma1 can act in the mother cell to stimulate Vac17 breakdown, but that this is normally counteracted by Kin4 and Frk1. To test this directly, we expressed the Vac17 MIS mutant *vac17-R135E,K138D-ProtA* in *vac17Δ* or *kin4Δfrk1Δvac17Δ* cells. This Vac17 mutant accumulated in mother cells at an increased level dependent upon the presence of *KIN4* and *FRK1* (Fig. 5A,E). Subsequently, we introduced point mutation *S222A* or *T240A* into *vac17-R135E,K138D* and expressed each triple mutant in *kin4Δfrk1Δvac17Δ* cells. Importantly, western blot analysis revealed that inhibition of Cla4-/Dma1-dependent degradation increased the levels of Vac17 (Fig. 6H). Similarly, as shown above, if Vac17 accumulated in mother cells because of a Myo2 mutation (*myo2-D1297N*) that inhibits binding to Vac17 and, consequently, blocks vacuole inheritance, Vac17 levels increase (Fig. S5A) (Yau et al., 2014). In *myo2-D1297N* cells lacking *KIN4* and *FRK1* (*myo2-D1297N kin4Δfrk1Δ*), this increase was no longer observed as a consequence of increased turnover (Fig. S5B,C,D). However, in contrast to Vac17, *vac17-S222A* levels were increased in *myo2-D1297N kin4Δfrk1Δ* cells (Fig. S5E). We conclude that both Cla4 and Dma1 stimulated the degradation of Vac17 in the mother cell but that Kin4 and Frk1 antagonise this activity, thereby protecting Vac17 from breakdown.

### A general mechanism for vacuole and peroxisome transport involving common factors

**Fig. 7.**
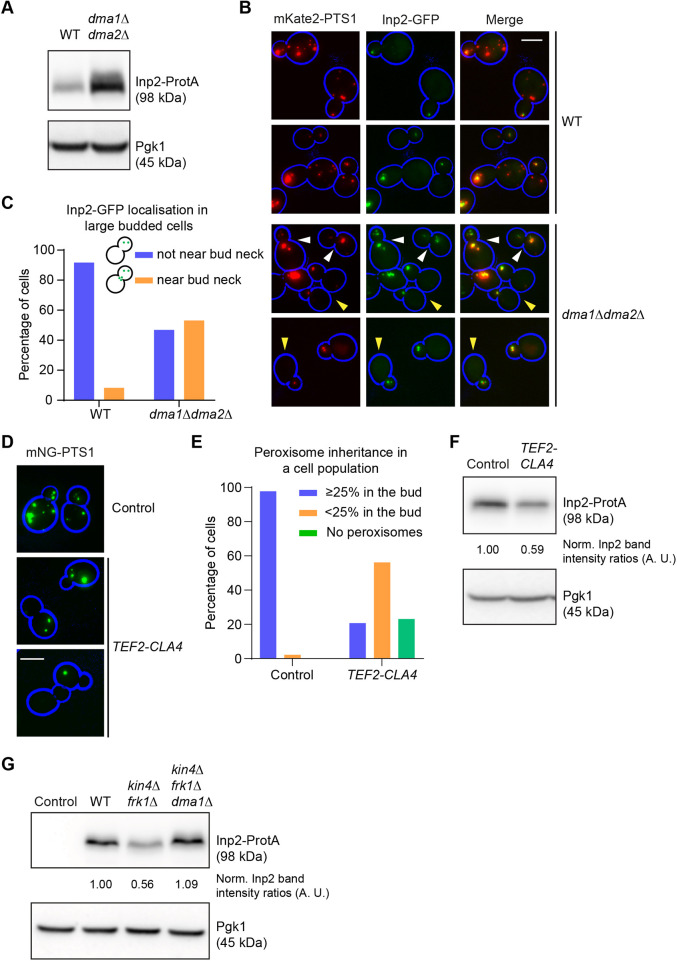
**A general mechanism for vacuole and peroxisome transport involving common factors.** (A) Inp2-ProtA protein levels are elevated in *dma1Δdma2Δ* cells compared to WT cells. Cell extracts were analysed by western blotting. Blots shown are representative of two independent experiments. (B) Inp2-GFP accumulates at the bud-neck in the large-budded *dma1Δdma2Δ* cells. Representative epifluorescence microscopy images of cells expressing Inp2-GFP (green) and the peroxisomal marker mKate2-PTS1 (red). Cell circumferences are highlighted in blue. White arrowheads indicate the Inp2-GFP at the bud neck, yellow arrowheads indicate cells without peroxisomes in the mother cell. (C) Quantification of Inp2-GFP localisation for cells as in B. A minimum of 81 large-budded cells were inspected for each strain. (D) Cla4 overexpression leads to defects in peroxisome transport to the bud and hence inheritance. Cla4 overexpression was achieved by introducing a strong constitutive promoter (*TEF2*) in the genome upstream to the *CLA4* coding sequence. Representative epifluorescence microscopy images of cells expressing the peroxisomal marker mNeonGreen(mNG)-PTS1 (green). Cell circumferences are highlighted in blue. (E) Quantification of peroxisome inheritance in a cell population as described in D. Cells (control, *n*=410 and *TEF2-CLA4* overexpression, *n*=381) were visually inspected. (F) Cla4 overexpression leads to reduced steady-state protein levels of Inp2-ProtA. Protein extracts from strains as shown in D expressing Inp2-ProtA were tested by western blotting. Blots shown are representative of two independent experiments. (G) Additional deletion of *DMA1* restored Inp2-ProtA levels in *kin4Δfrk1Δ* cells to levels comparable to those in WT cells. Cell extracts from indicated strains were analysed by western blotting. Blots shown are representative of two independent experiments. Inp2-ProtA/GFP expression was under the control of the *INP2* promoter in A, B, F and G. Pgk1 was used as a loading control in A, F and G. Inp2-ProtA band intensity was normalised against that of Pgk1. Normalised Inp2-ProtA signals in WT cells were set to 1 arbitrary unit (A.U.). All scale bars: 5 µm.

A defect in the timely degradation of Vac17 resulted in vacuoles mispositioning at the bud neck late during the cell cycle. Interestingly, in *dma1Δdma2Δ* cells, mispositioning of peroxisomes has also been reported (Yau et al., 2014). However, whether this is a result of increased Inp2 levels has not been reported. To study this, we analysed Inp2-ProtA protein levels in WT and *dma1Δdma2Δ* cells in western blots. Inp2-ProtA levels were clearly elevated in *dma1Δdma2Δ* cells compared to those in WT cells (Fig. 7A). Moreover, analysis of *dma1Δdma2Δ* cells expressing Inp2-GFP and the red peroxisomal marker mKate2-PTS1 showed an altered distribution of Inp2-GFP and peroxisomes. In *dma1Δdma2Δ* cells, peroxisomes were frequently lacking in mother cells and, when they were present, localised close to the bud neck. While Inp2-GFP in WT cells was mainly localised to peroxisomes within the bud, in *dma1Δdma2Δ* cells, Inp2-GFP was observed in both mother and daughter cells as long as the mother cells still contained peroxisomes (Fig. 7B,C). These results suggest that Inp2 levels are controlled by Dma1/2, and that this is important for proper peroxisome partitioning and positioning. Next, we analysed the effect of *CLA4* overexpression on peroxisome segregation. Interestingly, high expression of *CLA4* led to a clear defect in peroxisome transport to the bud (Fig. 7D,E) and *CLA4* overexpression led to reduced Inp2 levels compared to those in control (Fig. 7F). Previously, we have reported that *kin4Δfrk1Δ* cells fail to maintain steady-state protein levels of Inp2 (Ekal et al., 2023b), similar to the effect on Vac17 (Fig. 2A,B). Interestingly, Inp2 levels were restored in *kin4Δfrk1Δ* cells upon additional deletion of *DMA1* (Fig. 7G). These results demonstrated that transport of vacuoles and peroxisomes not only shares the requirement of Kin4 and Frk1 as regulators, but also Cla4 and Dma1/2.

## DISCUSSION

Previously, we have found that inheritance of vacuoles is strongly affected in *kin4Δfrk1Δ* cells (Ekal et al., 2023b). In this current study, we presented evidence supporting a model in which vacuole transport is controlled through the antagonistic relationship between Kin4/Frk1 and Cla4/Dma1. We showed that Kin4 and Frk1 are redundant in vacuole inheritance, and are required to maintain Vac17 steady-state levels. Our experiments showed that levels of the Vac17−Myo2 complex are, indeed, decreased in *kin4Δfrk1Δ* cells. However, they do not reveal whether this effect was solely due to a reduction in Vac17 levels or to possibly reduced affinity between Myo2 and Vac17. In line with this, in *kin4Δfrk1Δ* cells, vacuole segregation structures were formed but not − as observed in WT cells − maintained over long periods. Thus, a vacuole inheritance defect was observed in small- and large-budded *kin4Δfrk1Δ* cells. Vacuoles are essential for cell-cycle progression from G1 to S phase (Jin and Weisman, 2015), hence, in many buds of *kin4Δfrk1Δ* cells that fail to inherit vacuoles, they are formed *de novo*. This illustrates that Kin4 in SPOC is not required for *de novo* vacuole formation and maturation. Furthermore, we showed that SPOC is not required for vacuole inheritance and, therefore, that the role of Kin4 in this process is independent of its role in SPOC-dependent regulation of MEN (Fig. S4). Further analysis revealed that Elm1-dependent activation of Kin4 kinase activity is required for vacuole inheritance and to maintain Vac17 steady-state levels. Interestingly, similar to Kin4-T209A, Frk1-T209A failed to restore Vac17 protein levels and, thus, vacuole inheritance in *kin4Δfrk1Δ* cells. In addition, previously we have reported that cell toxicity caused by Frk1 overexpression can be alleviated by additional deletion of either *ELM1* or *BFA1* (Ekal et al., 2023b). These observations strongly hint towards Frk1 being a direct substrate for Elm1. Next, we performed Y2H and FM4-64 pulse-chase assays to validate previously characterised residues in the Vac17-MIS motif, which are crucial for interaction with Myo2 *in vivo*. Expression of *vac17-R135E,K138D* (MIS mutant) in *vac17Δ* cells not only failed to transport vacuoles to the growing bud but also resulted in elevated Vac17 protein levels compared to Vac17 WT levels and those accumulated on the vacuole within the mother cell. We found that *kin4Δfrk1Δ* cells failed to maintain these elevated MIS mutant protein levels in mother cells.

Next, we hypothesised that Cla4/Dma1 causes premature Vac17 breakdown in *kin4Δfrk1Δ* mother cells. In agreement with this, we showed that blockage of Cla4-/Dma1-dependent Vac17 breakdown − by deletion of either *CLA4* or *DMA1* − rescued the inheritance defect in *kin4Δfrk1Δ* cells. Moreover, expression of the *S222A* or *T240A* Vac17 mutants that are resistant to Cla4-/Dma1-dependent breakdown restored both vacuole inheritance and Vac17 protein levels in *kin4Δfrk1Δ* cells. Furthermore, by using the Vac17-MIS mutant, we showed that Vac17 is broken down in mother cells in a Cla4-/Dma1-dependent manner but that this is prevented by the presence of Frk1 and Kin4. In addition, high levels of Kin4 and Frk1 not only lead to decrease in Vac17 ubiquitylation and increased levels of Vac17 but also cause mispositioning of the vacuole in the bud, as reported previously for cells defective in timely Cla4-/Dma1-dependent Vac17 breakdown in the bud (Yau et al., 2014, 2017). We, therefore, conclude that Kin4 and Frk1 negatively regulate Cla4-/Dma1-dependent Vac17 degradation and transport termination. This conclusion was further supported by genetic data showing that disruption of Cla4-/Dma1-dependent Vac17 breakdown restored vacuole inheritance in *kin4Δfrk1Δ* cells. Thus, *kin4Δfrk1Δ* cells may initiate but fail to maintain vacuole transport, as Kin4 is not inhibiting Cla4-/Dma1-dependent Vac17 breakdown in mother cells and, consequently, Myo2 is prematurely released from vacuoles before they reach the bud.

Previously we have found that Kin4 and Frk1 are required to maintain steady-state levels of Inp2, as observed for Vac17 (Ekal et al., 2023b). In this current study, we found that Inp2 is stabilised in *dma1Δdma2Δ* cells. Interestingly, *CLA4* overexpression led to failure in peroxisome transport to the growing bud. Moreover, steady-state protein levels of Inp2 are reduced upon *CLA4* overexpression. The above results support a model in which the machinery that regulates vacuole transport also – and in a very similar manner − regulates peroxisome transport, although further detailed studies are required to corroborate this hypothesis. Furthermore, Cla4 and Dma1/2 also regulate turnover of the mitochondrial Myo2 receptor Mmr1 and, thus, maintain mitochondrial homeostasis (Nayef et al., 2024; Obara et al., 2022), which suggests a generic mode of action for Cla4/Dma1/2 in the regulation of multiple organelles within the cell.

**Fig. 8.**
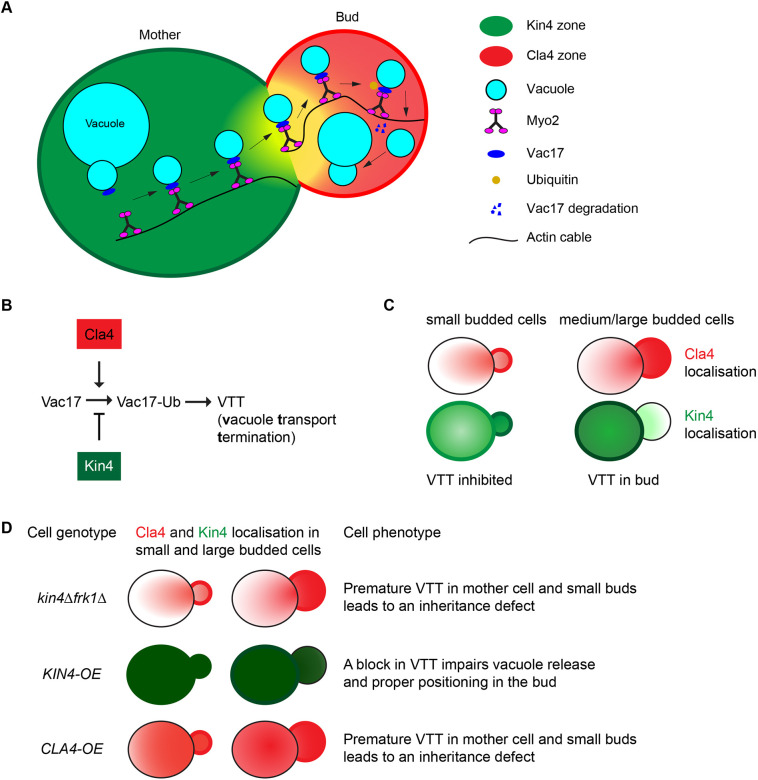
**Proposed schematic showing antagonistic roles of Kin4 and Cla4 during spatial and temporal regulation of organelle transport.** (A) Proposed zone model for vacuole transport in medium- to large-budded cells, in which Kin4 and Cla4 are preferentially concentrated in the mother cell (green zone) and bud (red zone), respectively, and have antagonistic activities to regulate vacuole transport in a spatial and temporal manner. (B) Cla4 promotes Vac17 ubiquitylation and subsequent vacuole transport termination (VTT) (red zone). In contrast, Kin4 prevents premature VTT in the mother cell by antagonising Cla4-/Dma1-dependent Vac17 ubiquitylation and degradation (green zone). (C) Representative Kin4 and Cla4 localisation during different stages of the cell cycle, which explains how vacuole transport is maintained and terminated in cells with small- to medium-sized buds versus cells with large buds. (D) Kin4 and Cla4 act similar to intracellular morphogens. Changes in the amounts of these opposing kinases triggered by cell-cycle progression or genetic manipulation either allows or prevents Cla4-/Dma1-dependent Vac17 ubiquitylation and, thus, determine the vacuole positioning and inheritance during cell growth and division.

Although Cla4 activity has been shown to trigger vacuole transport termination (VTT) in large buds through stimulating ubiquitylation of Vac17, our observations show that Cla4-/Dma1-dependent Vac17 breakdown is not limited to large buds. It can also occur in mother cells but this is counteracted in WT cells by Kin4. Similar to the zone model explaining regulation of MEN (Fig. S1B,C), a model can be proposed to explain VTT in large-budded cells (Fig. 8A,B). In this model Kin4 prevents Vac17 degradation and premature VTT in the mother cell, whereas Cla4 stimulates VTT through Dma1-dependent Vac17 degradation in the bud. Kin4 is concentrated at the cortex of large-budded mother cells and, therefore, unable to inhibit Cla4-/Dma1-dependent degradation in large buds resulting in VTT (Fig. 8A,B).

Our results also provide an explanation for the conundrum as to how the transport of vacuoles is maintained in cells with small buds over extended periods of time (Fig. 8C). During S phase, in small-budded cells, localisation of both Cla4 and Kin4 is highly polarised to the growing bud cortex (Bartholomew and Hardy, 2009; Falk et al., 2011; Holly and Blumer, 1999; Pereira and Schiebel, 2005), and we propose that Kin4 prevents premature Cla4-/Dma1-dependent Vac17 breakdown in the emerging bud and in the mother cell during this period of the cell cycle (Fig. 8C). In support of this model, overexpression experiments show that Kin4 can act in the bud to protect Vac17 from Cla4-dependent degradation (Fig. 8D). However, as the cell cycle progresses, Kin4 becomes more confined to the cortex of the mother cell, and Cla4 − located in the growing bud cortex − will be less inhibited by Kin4, allowing phosphorylation and degradation of Vac17 in medium- to large-sized buds (Fig. 8C). In this model, the two opposing kinases act as a sort of intracellular morphogen, directing VTT. Changes in the distribution of these opposing kinases, either during progression through the cell cycle or as a result of genetic manipulation, would either allow or prevent Cla4-/Dma1-dependent degradation of Vac17 (Fig. 8D). However, our model may be too simple, and additional regulatory factors might transfer spatial information and integrate it with temporal information. Many questions remain. For instance: What is the target for Kin4? Although we have been unable to identify Kin4-specific phosphorylation sites on Vac17, it remains possible that Kin4 phosphorylates Vac17 directly. Alternatively, Kin4 could inhibit Cla4 activity or Dma1 activity on Vac17 and Inp2 by regulating another factor.

The mechanisms unravelled by studying organelle dynamics in yeasts are of general relevance. For instance, in humans, PAKs have multiple important functions that are crucial for smooth mitotic progression. In some cancers, PAKs are hyperactivated, and this causes defects in chromosome segregation leading to multipolar spindle formation (Kumar et al., 2017). Melanosomes are organelles that synthesise and store melanin pigment. The dynamics of melanosomes between melanocytes and keratinocytes are crucial for hair and skin colour. The class V myosin Myo5a and its receptor Slac2 (melanophilin) play an important role in melanosome transport in actin-rich dendrites of melanocytes (Marks and Seabra, 2001). Slac2 harbours a PEST motif and the *slac2-PESTΔ* mutant is defective in degradation and leads to perinuclear aggregation of melanosomes (Fukuda and Itoh, 2004). Thus, control of melanosome transport and positioning resembles that of yeast vacuoles. Moreover, melanosome biogenesis is similar to that of lysosomes (Marks and Seabra, 2001). In conclusion, the regulatory principles of organelle dynamics seem to be conserved from the yeast to higher eukaryotes. Therefore, the study of organelle maintenance in *S*. *cerevisiae* is likely to provide molecular insights that could be extrapolated to higher eukaryotes including humans.

## MATERIALS AND METHODS

### Strains and plasmids

Yeast strains used in this study are derivatives of *S*. *cerevisiae* strains, BY4741 (MATa *his3Δ1 leu2Δ0 met15Δ0 ura3Δ0*) and BY4742 (MATα *his3Δ1 leu2Δ0 lys2Δ0 ura3Δ0*) and they are listed in Table S1. BY4741 and BY4742 were referred to as wild-type (WT) cells throughout the paper. Single to multiple gene deletions were generated by replacing the entire coding sequence of the gene of interest as described in (Ekal and Hettema, 2023). The pFA6a-GFP(S65T)-spHIS5 plasmid was used as a template for PCR to introduce the GFP-tag at the C-terminal of the *MYO2* open reading frame (ORF) in the genome.

Yeast expression plasmids used in this study were generated as described previously (Ekal et al., 2023a) and are listed in Table S2. Expression of Vph1-GFP was carried out under endogenous *VPH1* promoter. Constitutive expression of Kin4 (and its Kin4-T209A mutant) or Frk1 (and its Frk1-T209A mutant) was carried out under endogenous *KIN4* or *FRK1* promoters, respectively, and conditional expression of Kin4 and Frk1 was under the *GAL1/10* promoter. Expression of Vac17-ProtA/GFP or Inp2-ProtA/GFP was achieved using the native promoters of Vac17 or Inp2, respectively. C-terminal tagging of Vac17 and Inp2 does not affect their role in vacuole and peroxisome transport (Ekal et al., 2023b; Peng and Weisman, 2008; Tang et al., 2006). Vac17 point mutants were generated by sit-directed mutagenesis using PCRs and confirmed by sequencing analysis. Expression of the peroxisomal markers mKate2-PTS1 and mNG-PTS1 (Ekal et al., 2023a) was under the *HIS3* promoter, and expression of Inp2-ProtA and Inp2-GFP was performed as described previously (Ekal et al., 2023b).

### Growth conditions

Yeast cells were grown at 30°C in either rich yeast peptone (YP) medium (carbon source, 1% yeast extract, 2% peptone), yeast minimal (YM) medium 1 (carbon source, 0.17% yeast nitrogen base without amino acids and ammonium sulphate, 0.5% ammonium sulphate) for the selection of all prototrophic markers, or YM medium 2 (carbon source, 0.17% yeast nitrogen base without amino acids and ammonium sulphate, 0.5% ammonium sulphate, 1% casamino acids) for the selection of the uracil prototrophic marker. As carbon sources, 2% (w/v) of either glucose or raffinose or galactose were added. The amino acid and nucleic acid were prepared as 100× stocks and added to the minimal medium as required. For immunoblotting or fluorescence microscopy analysis, a preculture from overnight-grown yeast cells was diluted with the appropriate medium to OD_600_=0.1, and further grown to log phase (OD_600_=0.5−0.6). To induce galactose-based protein expression, precultured cells were diluted to a selective YM medium containing galactose at an OD_600_=0.3 and grown for 6−8 h before harvesting. For cycloheximide (CHX) assays, CHX (C-6255, Sigma-Aldrich) was added to yeast cell cultures at a final concentration of 35 µg/ml. After addition of CHX, cells were harvested at indicated time points and used for subsequent analysis. For quantification analysis, budding cells were considered as single cells.

### Image acquisition

Cells were grown to log phase before analysis using epifluorescence microscopy. Image acquisition was performed as described previously (Ekal et al., 2023a). Images were processed further using either Fiji-ImageJ-windows 64-bit software (v1.54f) (Schindelin et al., 2012) or Adobe Photoshop (version 24.7.0). To highlight the cell circumference, brightfield images were collected in one plane and processed where necessary in blue channel using Adobe Photoshop. Representative images from imaging experiments are shown.

### Vacuolar staining with FM4-64

Logarithmically growing cells (1−2 ml) were centrifuged (8000 ***g***, 2 min) and the cell pellet was resuspended in 200 μl yeast extract peptone dextrose (YPD) medium containing FM4-64 (Invitrogen, T3166, 1 ng/μl final concentration). The FM4-64 staining was performed at 30°C for 1 h, following which the cells were centrifuged (8000 ***g***, 2 min) and the supernatant was removed. The remaining cell pellet was washed thrice in YM medium. Subsequently, the cells were resuspended in 3−4 ml of fresh yeast minimal medium and incubated at 30°C for 4−5 h before imaging with an epifluorescence microscope.

### Time-lapse imaging

WT and *kin4Δfrk1Δ* cells expressing Vph1-GFP under an endogenous promoter were grown to log phase. Cells were harvested and stained with FM4-64 as described above. For time-lapse imaging, 20 μl cell suspension in YM medium was immobilised within a (2% w/v) agarose gel pad in 35 mm μ-dish (Ibidi). Cells were spread uniformly by gently pressing on top of the gel pad. The agarose gel pads were prepared as described by Ekal et al., (2023a,b). Fluorescence images were collected as 0.5 μm *z*-stacks for every 10 min time point. Images were processed using Fiji-ImageJ software and Adobe Photoshop.

### Immunoblotting

To analyse steady-state protein levels, protein extracts from logarithmically growing cells were prepared as described by Ekal et al., (2023a). Briefly, cells were lysed in a buffer containing 0.2 M NaOH and 0.2% β-mercaptoethanol, followed by protein precipitation using 5% trichloroacetic acid. Precipitated protein pellets were obtained by centrifugation and the pellets were resuspended in 10 μl 1 M Tris-HCl pH 9.4 and 90 μl 1×SDS–PAGE loading buffer, and denatured by boiling. Protein samples (OD_600_=0.25–1 equivalent) were used for SDS–PAGE and further analysed by immunoblotting as reported previously (Ekal et al., 2023a). Myo2-GFP detection was with a monoclonal anti-GFP antibody (mouse IgG monoclonal antibody clone 7.1 and 13.1; 1:3000; Roche, #11814460001). ProtA-tagged Vac17 and Inp2 were detected by the peroxidase-anti peroxidase (PAP) antibody (rabbit; 1:4000; Sigma-Aldrich, #P1291). Myc-tagged ubiquitin (Myc-Ub) was detected by anti-Myc antibody clone 9E10 (mouse; 1:5000; Sigma-Aldrich, #M4439). Pgk1 was used as loading control and detected by a monoclonal anti-Pgk1 antibody (anti-mouse; 1:7000; Invitrogen, #459250). The secondary antibody was an HRP-linked anti-mouse polyclonal (goat; 1:4000; Bio-Rad, 1706516). Blots were incubated using enhanced chemiluminescence reagents (ECL, GE Healthcare) and protein bands were visualised by chemiluminescence imaging. See Fig. S8 for uncropped blot images.

### Coimmunoprecipitation

For immunoprecipitation experiments, we transformed Myo2–GFP-expressing cells with a centromeric plasmid containing Vac17-ProtA under *VAC17* promoter or an empty plasmid (Ycplac33). Myo2-GFP was affinity purified using GFP Trap agarose resins (GFP-Trap Agarose, GTA, ChromoTek) as described earlier (Ekal et al., 2023a). Affinity-purified samples were denatured by boiling and analysed by western blotting. Myo2-GFP was detected using anti-GFP antibody and Vac17–ProtA was detected using PAP. For further information see also ‘Immunoblotting’ section.

### Yeast-two hybrid assay

For yeast-two hybrid analysis, MATa and MATα of the *S. cerevisiae* strain PJ69-4A were used (Eves et al., 2012). MATa cells were transformed with plasmid encoding an activation domain fused to either wild-type Myo2 or the Myo2 point mutants as well as the *LEU2* gene for auxotrophic selection. MATα cells were transformed with plasmid encoding a binding domain fused to either wild-type Vac17 or the Vac17 point mutants as well as *TRP1* gene for auxotrophic selection. Transformed MATa and MATα cells were mated on a plate containing YPD-rich medium for 1 day, shifted to YM+Glu medium lacking leucine and tryptophan, and grown for another 2 days to select diploids. Selected diploid cells were grown for 3−4 days on YM+Glu (Leu-Trp-Ade-His-) medium supplemented with variable concentrations of 3-aminotriazole (3AT) (3 mM, 6 mM and 10 mM) or not. Finally, plates were imaged using the same setting for all images.

### *In vivo* ubiquitylation assay

To detect ubiquitylated Vac17, *in vivo* ubiquitylation assay was performed as described before (Yau et al., 2014). Briefly, *bfa1Δ* cells expressing Kin4 under the endogenous *KIN4* promoter or the inducible *GAL1/10* promoter were transformed with *VAC17-GFP* and *Myc-Ub* plasmids. Cells were grown overnight in an appropriate YM medium supplemented with 2% (w/v) raffinose as a carbon source. The next day, the cells were diluted into YP rich medium containing 2% (w/v) galactose to induce *GAL-KIN4* expression. Cells were grown for 4 h during the day, Myc-Ub expression was induced by addition of CuCl_2_ (100 µM final concentration) and cells grown for another 4 h. After induction, 100 OD_600_-equivalent cells were harvested by centrifugation. Vac17-GFP was affinity purified from cell extracts using GFP Trap agarose resins, as described previously (Ekal et al., 2023a) with a minor modification in the buffer, i.e. containing 25 mM Tris-Cl pH 7.2, 150 mM NaCl, 100 mM β-glycerol phosphate, 25 mM NaF, 1 mM EGTA, 1 mM MgCl2, 0.15% Tween-20, protease inhibitor cocktail and 20 mM N-ethylmaleimide. The immunoprecipitated proteins were denatured by boiling, followed by western blotting. Vac17-GFP was detected with anti-GFP antibody, Myc-Ub was detected using anti-Myc antibody. For more details see the ‘Immunoblotting’ section above.

### Quantification and statistical analysis

For epifluorescence imaging experiments, budding cells were considered to be individual cells. Images were inspected manually using the Volocity software and (version 7.0.0, Quorum technologies Inc). For quantification of protein levels, western blots showing unsaturated protein bands were analysed in Image Lab software (version 6.1, Bio-Rad). For plotting graphs and statistical analysis GraphPad Prism 10.0.0 (153) (accessed on 12 June 2023) software was used. Statistical analysis was performed using a paired *t*-test or a one-way ANOVA test or a two-way ANOVA test as indicated. ****P<0.0001; ***P<0.005; **P<0.01; *P<0.05; non-significant (ns), *P*>0.05.

## Supplementary Material



10.1242/joces.261948_sup1 Supplementary information

## References

[JCS261948C1] Bartholomew, C. R. and Hardy, C. F. (2009). p21-activated kinases Cla4 and Ste20 regulate vacuole inheritance in Saccharomyces cerevisiae. *Eukaryot. Cell* 8, 560-572. 10.1128/EC.00111-08 19218422 PMC2669209

[JCS261948C2] Beach, D. L., Thibodeaux, J., Maddox, P., Yeh, E. and Bloom, K. (2000). The role of the proteins Kar9 and Myo2 in orienting the mitotic spindle of budding yeast. *Curr. Biol.* 10, 1497-1506. 10.1016/S0960-9822(00)00837-X 11114516

[JCS261948C3] Bertazzi, D. T., Kurtulmus, B. and Pereira, G. (2011). The cortical protein Lte1 promotes mitotic exit by inhibiting the spindle position checkpoint kinase Kin4. *J. Cell Biol.* 193, 1033-1048. 10.1083/jcb.201101056 21670215 PMC3115795

[JCS261948C4] Bi, E. and Park, H. O. (2012). Cell polarization and cytokinesis in budding yeast. *Genetics* 191, 347-387. 10.1534/genetics.111.132886 22701052 PMC3374305

[JCS261948C5] Caydasi, A. K. and Pereira, G. (2012). SPOC alert--when chromosomes get the wrong direction. *Exp. Cell Res.* 318, 1421-1427. 10.1016/j.yexcr.2012.03.031 22510435

[JCS261948C6] Caydasi, A. K., Kurtulmus, B., Orrico, M. I., Hofmann, A., Ibrahim, B. and Pereira, G. (2010). Elm1 kinase activates the spindle position checkpoint kinase Kin4. *J. Cell Biol.* 190, 975-989. 10.1083/jcb.201006151 20855503 PMC3101594

[JCS261948C7] Caydasi, A. K., Khmelinskii, A., Duenas-Sanchez, R., Kurtulmus, B., Knop, M. and Pereira, G. (2017). Temporal and compartment-specific signals coordinate mitotic exit with spindle position. *Nat. Commun.* 8, 14129. 10.1038/ncomms14129 28117323 PMC5286211

[JCS261948C8] Chan, L. Y. and Amon, A. (2009). The protein phosphatase 2A functions in the spindle position checkpoint by regulating the checkpoint kinase Kin4. *Genes Dev.* 23, 1639-1649. 10.1101/gad.1804609 19605686 PMC2714715

[JCS261948C9] Ekal, L. and Hettema, E. (2023). Targeted modifications of the yeast genome to study peroxisomes. *Methods Mol. Biol.* 2643, 217-232. 10.1007/978-1-0716-3048-8_16 36952189

[JCS261948C10] Ekal, L., Alqahtani, A. M. and Hettema, E. H. (2023a). The dynamin-related protein Vps1 and the peroxisomal membrane protein Pex27 function together during peroxisome fission. *J. Cell Sci.* 136, jcs246348. 10.1242/jcs.246348 36825558 PMC10112978

[JCS261948C11] Ekal, L., Alqahtani, A. M. S., Schuldiner, M., Zalckvar, E., Hettema, E. H. and Ayscough, K. R. (2023b). Spindle position checkpoint kinase Kin4 regulates organelle transport in saccharomyces cerevisiae. *Biomolecules* 13, 1098. 10.3390/biom13071098 37509134 PMC10377308

[JCS261948C12] Eves, P. T., Jin, Y., Brunner, M. and Weisman, L. S. (2012). Overlap of cargo binding sites on myosin V coordinates the inheritance of diverse cargoes. *J. Cell Biol.* 198, 69-85. 10.1083/jcb.201201024 22753895 PMC3392941

[JCS261948C13] Falk, J. E., Chan, L. Y. and Amon, A. (2011). Lte1 promotes mitotic exit by controlling the localization of the spindle position checkpoint kinase Kin4. *Proc. Natl. Acad. Sci. USA* 108, 12584-12590. 10.1073/pnas.1107784108 21709215 PMC3150932

[JCS261948C14] Falk, J. E., Tsuchiya, D., Verdaasdonk, J., Lacefield, S., Bloom, K. and Amon, A. (2016). Spatial signals link exit from mitosis to spindle position. *Elife* 5, e14036. 10.7554/eLife.14036 27166637 PMC4887205

[JCS261948C15] Fukuda, M. and Itoh, T. (2004). Slac2-a/melanophilin contains multiple PEST-like sequences that are highly sensitive to proteolysis. *J. Biol. Chem.* 279, 22314-22321. 10.1074/jbc.M401791200 15145961

[JCS261948C16] Hill, K. L., Catlett, N. L. and Weisman, L. S. (1996). Actin and myosin function in directed vacuole movement during cell division in Saccharomyces cerevisiae. *J. Cell Biol.* 135, 1535-1549. 10.1083/jcb.135.6.1535 8978821 PMC2133941

[JCS261948C17] Hoepfner, D., Van Den Berg, M., Philippsen, P., Tabak, H. F. and Hettema, E. H. (2001). A role for Vps1p, actin, and the Myo2p motor in peroxisome abundance and inheritance in Saccharomyces cerevisiae. *J. Cell Biol.* 155, 979-990. 10.1083/jcb.200107028 11733545 PMC2150915

[JCS261948C18] Holly, S. P. and Blumer, K. J. (1999). PAK-family kinases regulate cell and actin polarization throughout the cell cycle of Saccharomyces cerevisiae. *J. Cell Biol.* 147, 845-856. 10.1083/jcb.147.4.845 10562285 PMC2156167

[JCS261948C19] Ishikawa, K., Catlett, N. L., Novak, J. L., Tang, F., Nau, J. J. and Weisman, L. S. (2003). Identification of an organelle-specific myosin V receptor. *J. Cell Biol.* 160, 887-897. 10.1083/jcb.200210139 12642614 PMC2173761

[JCS261948C20] Jin, Y. and Weisman, L. S. (2015). The vacuole/lysosome is required for cell-cycle progression. *Elife* 4, e08160. 10.7554/eLife.08160 26322385 PMC4586482

[JCS261948C21] Knoblach, B. and Rachubinski, R. A. (2015). Sharing the cell's bounty - organelle inheritance in yeast. *J. Cell Sci.* 128, 621-630. 10.1242/jcs.151423 25616900

[JCS261948C22] Kumar, R., Sanawar, R., Li, X. and Li, F. (2017). Structure, biochemistry, and biology of PAK kinases. *Gene* 605, 20-31. 10.1016/j.gene.2016.12.014 28007610 PMC5250584

[JCS261948C23] Lagrassa, T. J. and Ungermann, C. (2005). The vacuolar kinase Yck3 maintains organelle fragmentation by regulating the HOPS tethering complex. *J. Cell Biol.* 168, 401-414. 10.1083/jcb.200407141 15684030 PMC2171739

[JCS261948C24] Legesse-Miller, A., Zhang, S., Santiago-Tirado, F. H., Van Pelt, C. K. and Bretscher, A. (2006). Regulated phosphorylation of budding yeast's essential myosin V heavy chain, Myo2p. *Mol. Biol. Cell* 17, 1812-1821. 10.1091/mbc.e05-09-0872 16467380 PMC1415295

[JCS261948C25] Li, K. W., Lu, M. S., Iwamoto, Y., Drubin, D. G. and Pedersen, R. T. A. (2021). A preferred sequence for organelle inheritance during polarized cell growth. *J. Cell Sci.* 134, jcs258856. 10.1242/jcs.258856 34622919 PMC8627559

[JCS261948C26] Liu, Y., Li, L., Yu, C., Zeng, F., Niu, F. and Wei, Z. (2022). Cargo recognition mechanisms of yeast Myo2 revealed by AlphaFold2-powered protein complex prediction. *Biomolecules* 12, 1032. 10.3390/biom12081032 35892342 PMC9330073

[JCS261948C27] Maekawa, H., Priest, C., Lechner, J., Pereira, G. and Schiebel, E. (2007). The yeast centrosome translates the positional information of the anaphase spindle into a cell cycle signal. *J. Cell Biol.* 179, 423-436. 10.1083/jcb.200705197 17967947 PMC2064790

[JCS261948C28] Marks, M. S. and Seabra, M. C. (2001). The melanosome: membrane dynamics in black and white. *Nat. Rev. Mol. Cell Biol.* 2, 738-748. 10.1038/35096009 11584301

[JCS261948C29] Merlini, L., Fraschini, R., Boettcher, B., Barral, Y., Lucchini, G. and Piatti, S. (2012). Budding yeast dma proteins control septin dynamics and the spindle position checkpoint by promoting the recruitment of the Elm1 kinase to the bud neck. *PLoS Genet.* 8, e1002670. 10.1371/journal.pgen.1002670 22570619 PMC3343086

[JCS261948C30] Miller, R. K. and Rose, M. D. (1998). Kar9p is a novel cortical protein required for cytoplasmic microtubule orientation in yeast. *J. Cell Biol.* 140, 377-390. 10.1083/jcb.140.2.377 9442113 PMC2132572

[JCS261948C31] Moore, J. K., Chudalayandi, P., Heil-Chapdelaine, R. A. and Cooper, J. A. (2010). The spindle position checkpoint is coordinated by the Elm1 kinase. *J. Cell Biol.* 191, 493-503. 10.1083/jcb.201006092 21041444 PMC3003319

[JCS261948C32] Moriya, H. and Isono, K. (1999). Analysis of genetic interactions between DHH1, SSD1 and ELM1 indicates their involvement in cellular morphology determination in Saccharomyces cerevisiae. *Yeast* 15, 481-496. 10.1002/(SICI)1097-0061(199904)15:6<481::AID-YEA391>3.0.CO;2-M 10234786

[JCS261948C33] Nayef, N., Ekal, L., Hettema, E. H. and Ayscough, K. R. (2024). Insights into the regulation of the mitochondrial inheritance and trafficking adaptor protein Mmr1 in Saccharomyces cerevisiae. *Kinases Phosphatases.* 2, 190-208. 10.3390/kinasesphosphatases2020012

[JCS261948C34] Obara, K., Yoshikawa, T., Yamaguchi, R., Kuwata, K., Nakatsukasa, K., Nishimura, K. and Kamura, T. (2022). Proteolysis of adaptor protein Mmr1 during budding is necessary for mitochondrial homeostasis in Saccharomyces cerevisiae. *Nat. Commun.* 13, 2005. 10.1038/s41467-022-29704-8 35422486 PMC9010424

[JCS261948C35] Peng, Y. and Weisman, L. S. (2008). The cyclin-dependent kinase Cdk1 directly regulates vacuole inheritance. *Dev. Cell* 15, 478-485. 10.1016/j.devcel.2008.07.007 18804442 PMC2727752

[JCS261948C36] Pereira, G. and Schiebel, E. (2005). Kin4 kinase delays mitotic exit in response to spindle alignment defects. *Mol. Cell* 19, 209-221. 10.1016/j.molcel.2005.05.030 16039590

[JCS261948C37] Pereira, G., Hofken, T., Grindlay, J., Manson, C. and Schiebel, E. (2000). The Bub2p spindle checkpoint links nuclear migration with mitotic exit. *Mol. Cell* 6, 1-10. 10.1016/S1097-2765(05)00017-1 10949022

[JCS261948C38] Peter, M., Neiman, A. M., Park, H. O., Van Lohuizen, M. and Herskowitz, I. (1996). Functional analysis of the interaction between the small GTP binding protein Cdc42 and the Ste20 protein kinase in yeast. *EMBO J.* 15, 7046-7059. 10.1002/j.1460-2075.1996.tb01096.x 9003780 PMC452530

[JCS261948C39] Pruyne, D. and Bretscher, A. (2000a). Polarization of cell growth in yeast. *J. Cell Sci.* 113, 571-585. 10.1242/jcs.113.4.571 10652251

[JCS261948C40] Pruyne, D. and Bretscher, A. (2000b). Polarization of cell growth in yeast. I. Establishment and maintenance of polarity states. *J. Cell Sci.* 113, 365-375. 10.1242/jcs.113.3.365 10639324

[JCS261948C41] Rechsteiner, M. and Rogers, S. W. (1996). PEST sequences and regulation by proteolysis. *Trends Biochem. Sci.* 21, 267-271. 10.1016/S0968-0004(96)10031-1 8755249

[JCS261948C42] Schindelin, J., Arganda-Carreras, I., Frise, E., Kaynig, V., Longair, M., Pietzsch, T., Preibisch, S., Rueden, C., Saalfeld, S., Schmid, B. et al. (2012). Fiji: an open-source platform for biological-image analysis. *Nat. Methods* 9, 676-682. 10.1038/nmeth.2019 22743772 PMC3855844

[JCS261948C43] Tang, F., Kauffman, E. J., Novak, J. L., Nau, J. J., Catlett, N. L. and Weisman, L. S. (2003). Regulated degradation of a class V myosin receptor directs movement of the yeast vacuole. *Nature* 422, 87-92. 10.1038/nature01453 12594460

[JCS261948C44] Tang, F., Peng, Y., Nau, J. J., Kauffman, E. J. and Weisman, L. S. (2006). Vac8p, an armadillo repeat protein, coordinates vacuole inheritance with multiple vacuolar processes. *Traffic* 7, 1368-1377. 10.1111/j.1600-0854.2006.00458.x 16824055

[JCS261948C45] Versele, M. and Thorner, J. (2004). Septin collar formation in budding yeast requires GTP binding and direct phosphorylation by the PAK, Cla4. *J. Cell Biol.* 164, 701-715. 10.1083/jcb.200312070 14993234 PMC2172161

[JCS261948C46] Vida, T. A. and Emr, S. D. (1995). A new vital stain for visualizing vacuolar membrane dynamics and endocytosis in yeast. *J. Cell Biol.* 128, 779-792. 10.1083/jcb.128.5.779 7533169 PMC2120394

[JCS261948C47] Weisman, L. S., Emr, S. D. and Wickner, W. T. (1990). Mutants of Saccharomyces cerevisiae that block intervacuole vesicular traffic and vacuole division and segregation. *Proc. Natl. Acad. Sci. USA* 87, 1076-1080. 10.1073/pnas.87.3.1076 1689059 PMC53413

[JCS261948C48] Weiss, E. L. (2012). Mitotic exit and separation of mother and daughter cells. *Genetics* 192, 1165-1202. 10.1534/genetics.112.145516 23212898 PMC3512134

[JCS261948C49] Wong, S. and Weisman, L. S. (2021). Roles and regulation of myosin V interaction with cargo. *Adv. Biol. Regul.* 79, 100787. 10.1016/j.jbior.2021.100787 33541831 PMC7920922

[JCS261948C50] Wong, S., Hepowit, N. L., Port, S. A., Yau, R. G., Peng, Y., Azad, N., Habib, A., Harpaz, N., Schuldiner, M., Hughson, F. M. et al. (2020). Cargo release from myosin V requires the convergence of parallel pathways that phosphorylate and ubiquitylate the cargo adaptor. *Curr. Biol.* 30, 4399-4412.e7. 10.1016/j.cub.2020.08.062 32916113 PMC8025699

[JCS261948C51] Yau, R. G., Peng, Y., Valiathan, R. R., Birkeland, S. R., Wilson, T. E. and Weisman, L. S. (2014). Release from myosin V via regulated recruitment of an E3 ubiquitin ligase controls organelle localization. *Dev. Cell* 28, 520-533. 10.1016/j.devcel.2014.02.001 24636257 PMC3994899

[JCS261948C52] Yau, R. G., Wong, S. and Weisman, L. S. (2017). Spatial regulation of organelle release from myosin V transport by p21-activated kinases. *J. Cell Biol.* 216, 1557-1566. 10.1083/jcb.201607020 28495836 PMC5461012

[JCS261948C53] Zhou, X., Li, W., Liu, Y. and Amon, A. (2021). Cross-compartment signal propagation in the mitotic exit network. *Elife* 10, e63645. 10.7554/eLife.63645 33481703 PMC7822594

