# Supplementary material for: Spatiotemporal regulation of organelle transport by spindle position checkpoint kinase Kin4
Source: J Cell Sci. 2024 Nov 13;137(21):jcs261948. doi: 10.1242/jcs.261948 (PMC11586526; doi:10.1242/jcs.261948)
Supplement: Supplementary information [file joces-137-261948-s1.pdf]

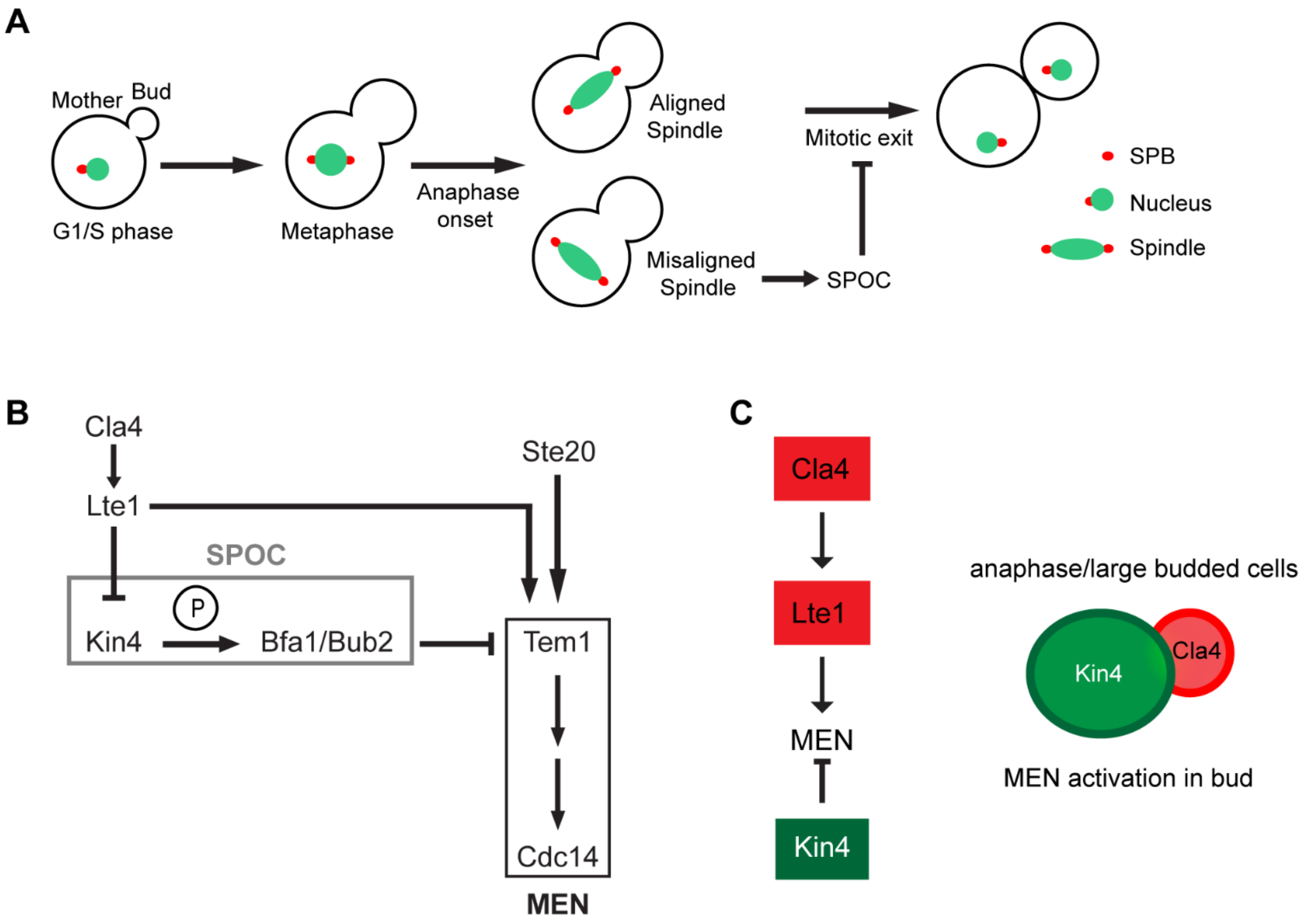

Fig. S1. A schematic model showing a cascade of events involved in the spindle position checkpoint (SPOC). (A) Nuclear inheritance in *S. cerevisiae* involves spindle pole body (SPB) duplication followed by spindle elongation and alignment along the cell-polarity axis before the onset of mitotic exit. At the onset of anaphase, a misaligned spindle leads to the activation of the spindle position checkpoint (SPOC) to delay the mitotic exit. (B) Schematic diagram showing the interplay between molecular players involved in (SPOC) and mitotic exit network (MEN). (C) Cla4 facilitates the MEN activation via promoting Lte1 activity in the bud whereas Kin4 negatively regulates MEN activation in the mother. The representative zone model displays Kin4 and Cla4 active zones in the mother and in the bud, respectively.

Journal of Cell Science • Supplementary information

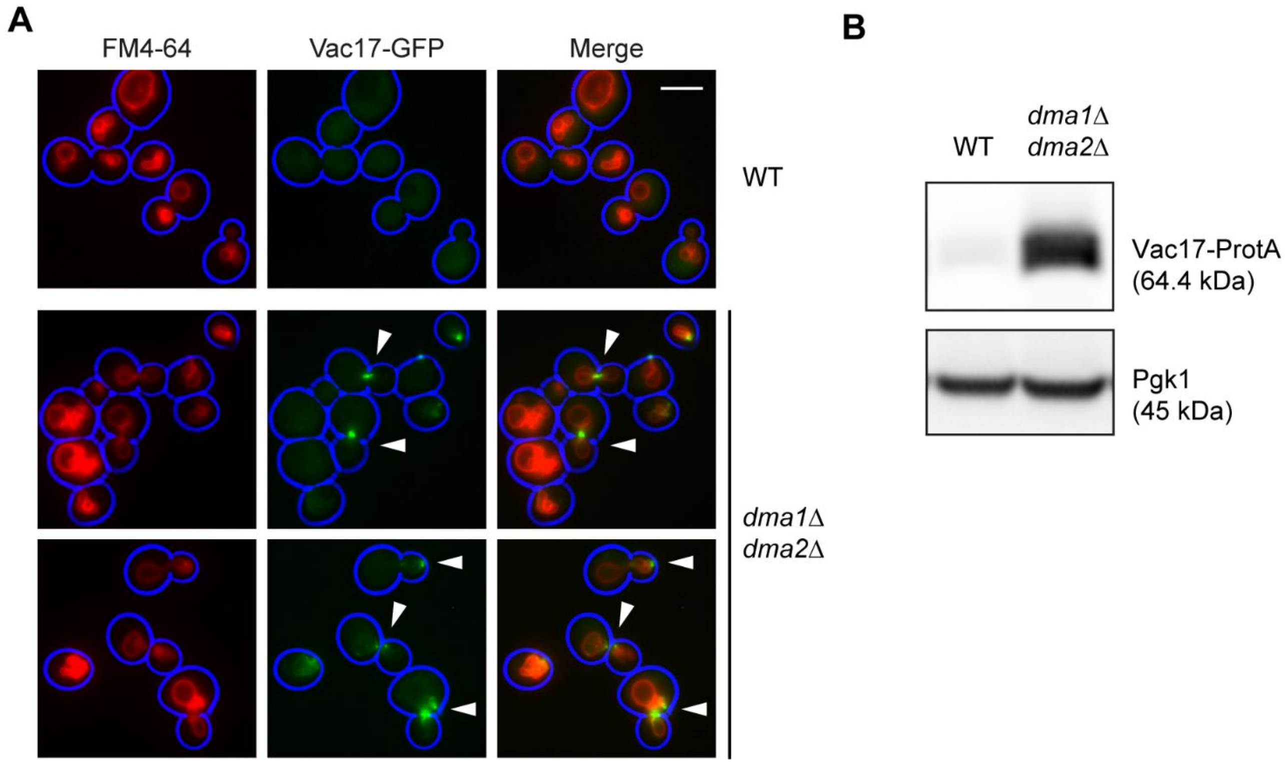

Fig. S2. In *dma1Δdma2Δ* cells Vac17 protein levels are elevated, further leading to inappropriate vacuole positioning in the bud. (A) Representative epifluorescence microscopy images of WT and *dma1Δdma2Δ* cells expressing Vac17-GFP (green) and pulse-chased with FM4-64 (red) are shown.
To highlight the cell circumference (blue), brightfield images were collected in one plane and processed where necessary in a blue channel using Adobe Photoshop. Arrowheads indicate Vac17-GFP accumulation at the bud tip and bud neck. Scale bar: 5 µm. (B) Cell extracts from WT and *dma1Δdma2Δ* cells expressing Vac17-ProtA were analysed by western blotting. Pgk1 was used as a loading control. Vac17-GFP and Vac17-ProtA were expressed from a centromeric plasmid under the control of the *VAC17* promoter.

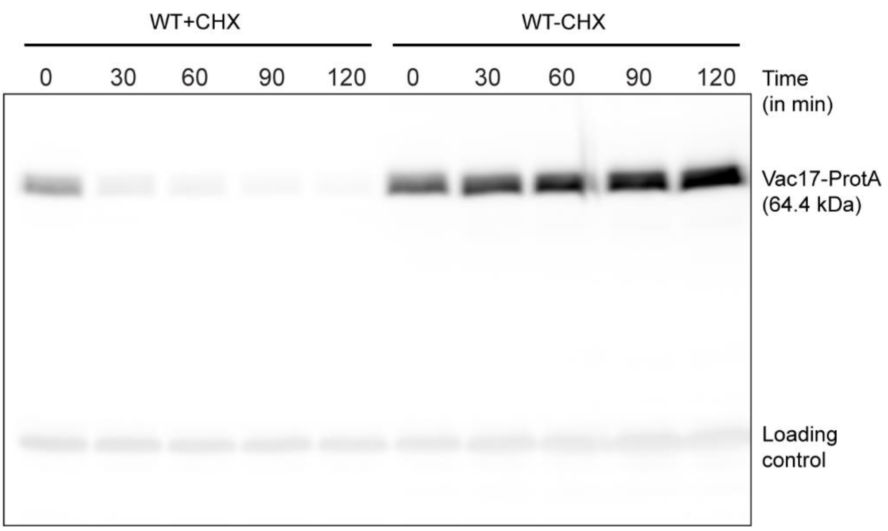

Fig. S3. Control experiment for cycloheximide (CHX) treatment for Vac17 steady-state analysis. WT cells expressing Vac17-ProtA from a plasmid under the control of its native promoter were treated with cycloheximide (CHX) and were harvested at different time points as described. Cell extracts were analysed by western blotting.

Journal of Cell Science • Supplementary information

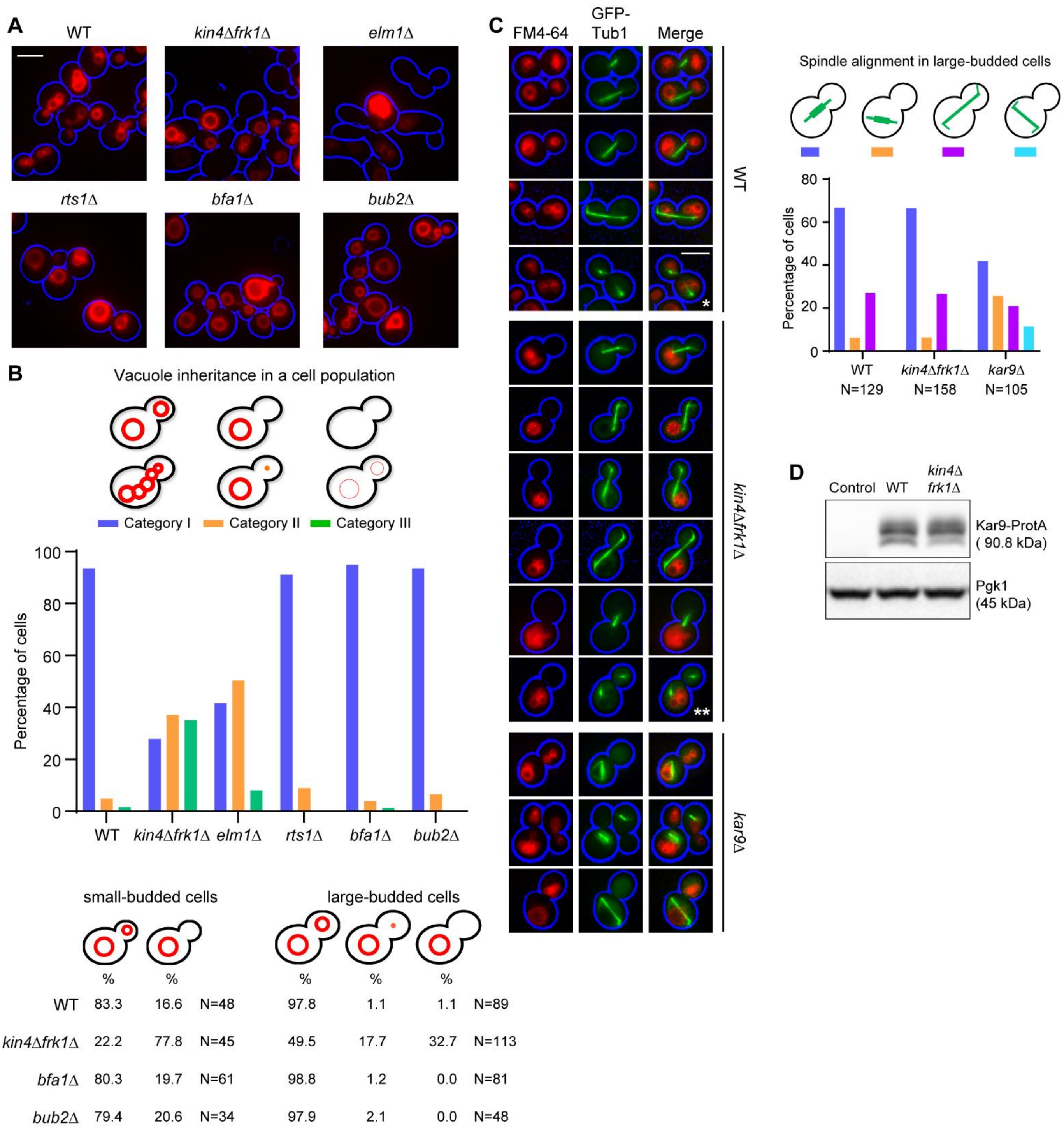

Fig. S4. Kin4 function in vacuole inheritance is independent of its role in SPOC. (A) Vacuole inheritance in *bfa1Δ, bub2Δ*, and *rts1Δ* cells is unaffected in contrast to in *kin4Δfrk1Δ* and *elm1Δ* cells. Epifluorescence microscopy analysis of vacuole inheritance in exponentially grown cells of the strains indicated, pulse-chase labelled with FM4-64 (red). Cell circumference is highlighted in blue. (B) Quantification of vacuole inheritance in a cell population for the strains in (A). A

Journal of Cell Science • Supplementary information

minimum of 113 budding cells were analysed per strain. Category I, bright FM4-64 staining of vacuoles in mother cells and buds; category II, bright FM4-64 staining in mother cells but not in buds; category III, cells lack bright FM4-64 staining altogether. Quantification of the presence of vacuoles in small- and large-budded cells was analysed separately for the indicated strains. (C) Proper spindle alignment occurs in large-budded *kin4Δfrk1Δ* cells with defects in vacuole inheritance. In contrast, *kar9Δ* cells with misaligned spindles show proper vacuole inheritance. WT, *kin4Δfrk1Δ*, and *kar9Δ* cells were transformed with a plasmid encoding mitotic spindle marker (GFP-Tub1, green). Transformed cells were pulse chased with FM4-64 (red) and analysed by fluorescence microscopy. Cell circumference is highlighted in blue. Scale bars: 5 µm. Quantification of the large-budded cells was performed to analyse the severity of spindle misorientation. The schematic diagram indicates the different categories of cells with aligned (blue and purple) or misaligned (orange and cyan) spindles. Cells with disassembled spindles that were either successful or unsuccessful in inheriting vacuoles are indicated by * and **, respectively. (D) Steady-state protein levels of Kar9 in *kin4Δfrk1Δ* cells are comparable to those in WT cells. Cell extracts from WT and *kin4Δfrk1Δ* cells expressing Kar9-ProtA from a plasmid under the control of the endogenous *KAR9* promoter were analysed by western blotting.

Journal of Cell Science • Supplementary information

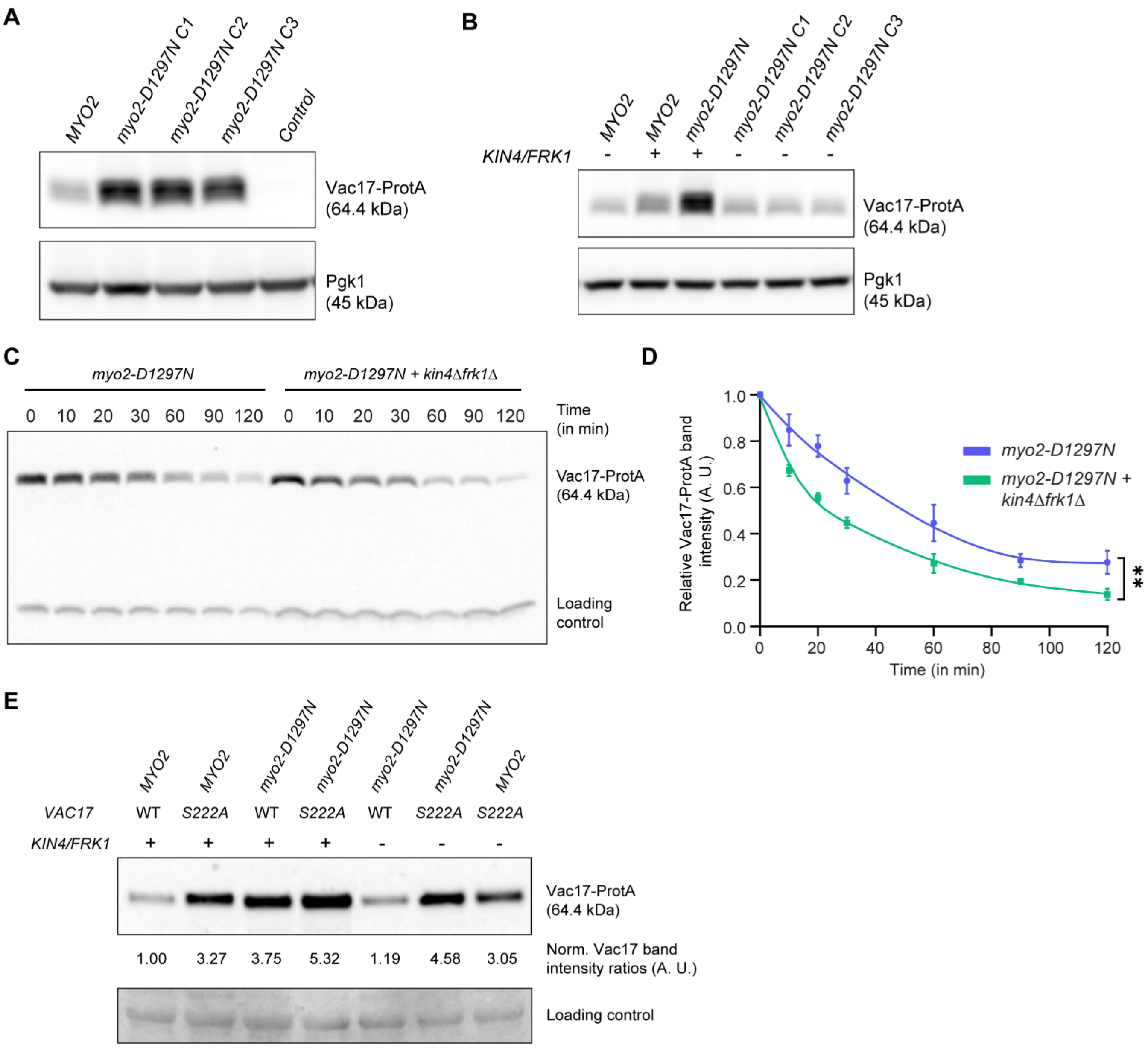

Fig. S5. Kin4 and Frk1 prevent premature Vac17 breakdown in the mother of *myo2-D1297N* cells. (A) *myo2-D1297N* cells express higher levels of Vac17 protein levels compared to cells expressing wild-type *MYO2*. (B) *myo2-D1297N* cells lacking *KIN4* and *FRK1* failed to maintain elevated Vac17 protein levels. Protein extracts from cells expressing Vac17-ProtA from a plasmid under the control of its endogenous promoter were analysed by western blot analysis. C1, C2, and C3 indicate three independent clones. (C) Vac17 turnover is increased in *myo2-D1297N* cells lacking *KIN4* and *FRK1*. Logarithmically grown cells were treated with Cycloheximide (CHX) and subsequently were harvested at indicated time points. Cell extracts from these cells were analysed by western blotting. (D) Quantification of

Journal of Cell Science • Supplementary information

Vac17-ProtA levels in (C) from 3 independent experiments. Error bars indicate standard error mean (±s.e.m.). Statistical significance was determined using a two-tailed paired t-test. **p=0.0025. (E) A block in Cla4-/Dma1-dependent Vac17 breakdown by expressing *vac17-S222A* in *myo2-D1297N-kin4Δfrk1Δ* cells restored elevated Vac17 protein levels. Protein extracts from cells expressing ProtA-tagged-Vac17 versions from plasmids under the control of the *VAC17* promoter were analysed by western blotting. Loading control was obtained by incubating the blot with Ponceau stain. For quantification of Vac17-ProtA protein levels, Vac17-ProtA band intensity was normalised against the loading control band intensity. Normalised Vac17-ProtA signals in WT cells were set to 1 arbitrary unit (A. U.).

Journal of Cell Science • Supplementary information

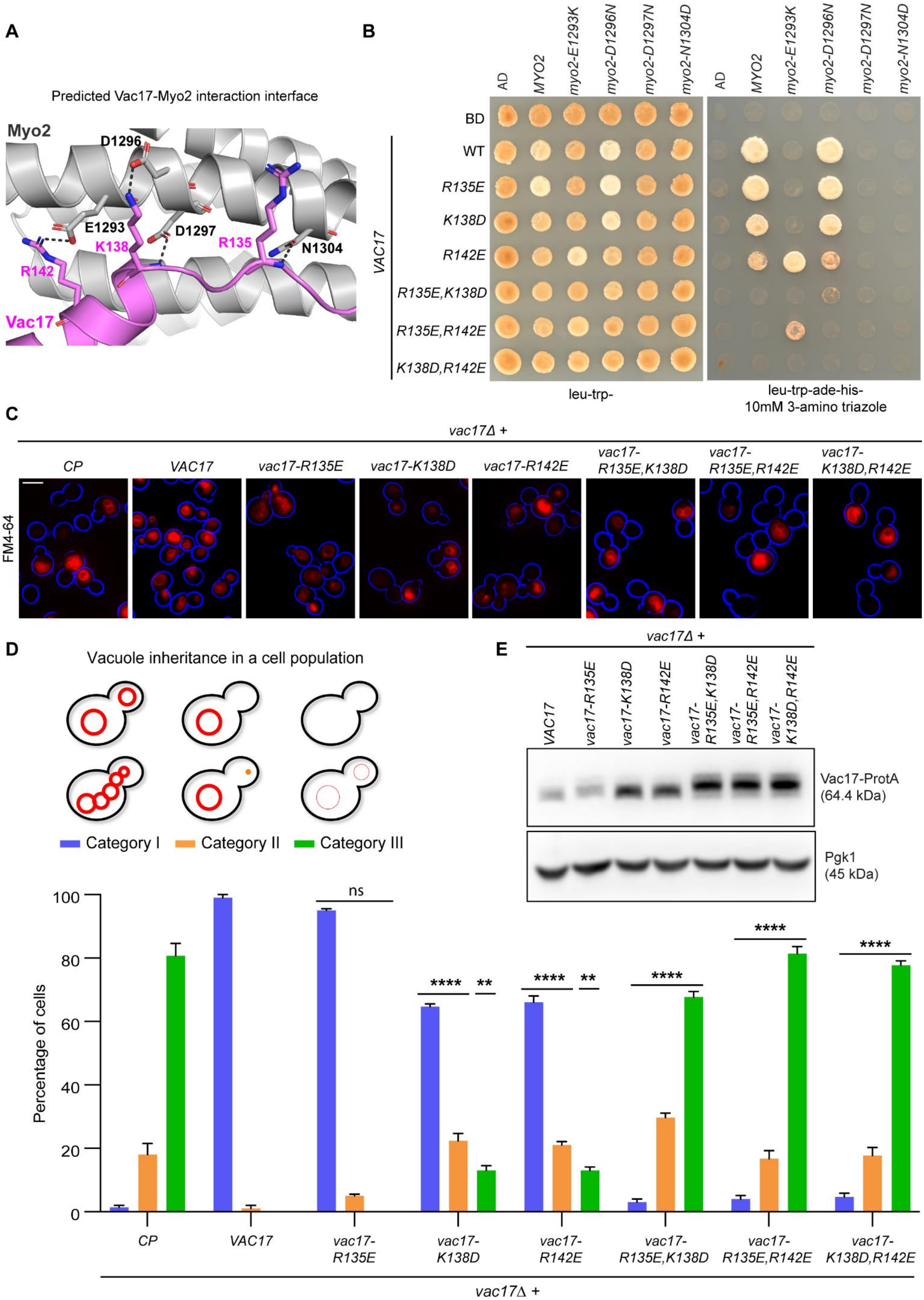

Journal of Cell Science • Supplementary information

Fig. S6. Characterisation and validation of myosin interaction site (MIS) motif in Vac17. (A) Cartoon representation of Vac17-Myo2 interaction interface; adapted from (Liu et al., 2022). The potential interactions between Vac17 (pink) and Myo2 (grey) residues are highlighted by the dotted black lines (B) Mutations in Vac17-MIS affect its interaction with Myo2. Yeast two-hybrid based growth assay was used to study the interaction between Vac17 and Myo2 *in vivo*. Briefly, Mata and Matα cells carrying Vac17 and Myo2 plasmids respectively were mated, and the diploids were selected on yeast minimal media supplemented with 10 mM 3 amino-triazole or without. (C) Vac17-MIS mutants lead to defects in vacuole inheritance. Epifluorescence microscopy analysis for *vac17Δ* cells expressing plasmids encoding C-terminally GFP-tagged either wild-type Vac17 or Vac17 containing point mutations in MIS motif. Exponentially grown cells were pulse chased with FM4-64 (red) and further analysed with epifluorescence microscopy. Cell circumference is highlighted in blue. Scale bar: 5 µm. (D) Quantification for the strains in (C). A minimum of 100 cells from 3 independent experiments were inspected manually to study vacuole inheritance in a cell population. Statistical analysis was performed using a two-way ANOVA (Turkey's multiple comparisons) test. Significance is shown in comparison to *vac17Δ* cells transformed with *VAC17*. **p=0.0010, ****p<0.0001, ns: not significant. Error bars indicate the standard error of the mean (+s.e.m.). Category I, bright FM4-64 staining of vacuoles in mother cells and buds; category II, bright FM4-64 staining in mother cells but not in buds; category III, cells lack bright FM4-64 staining altogether. (E) Steady-state protein levels of Vac17-MIS mutants are comparable to wild-type Vac17. ProtA-tagged Vac17 versions were expressed on plasmids under the control of the *VAC17* promoter in *vac17Δ* cells. Cell extracts were analysed by western blotting.

Journal of Cell Science • Supplementary information

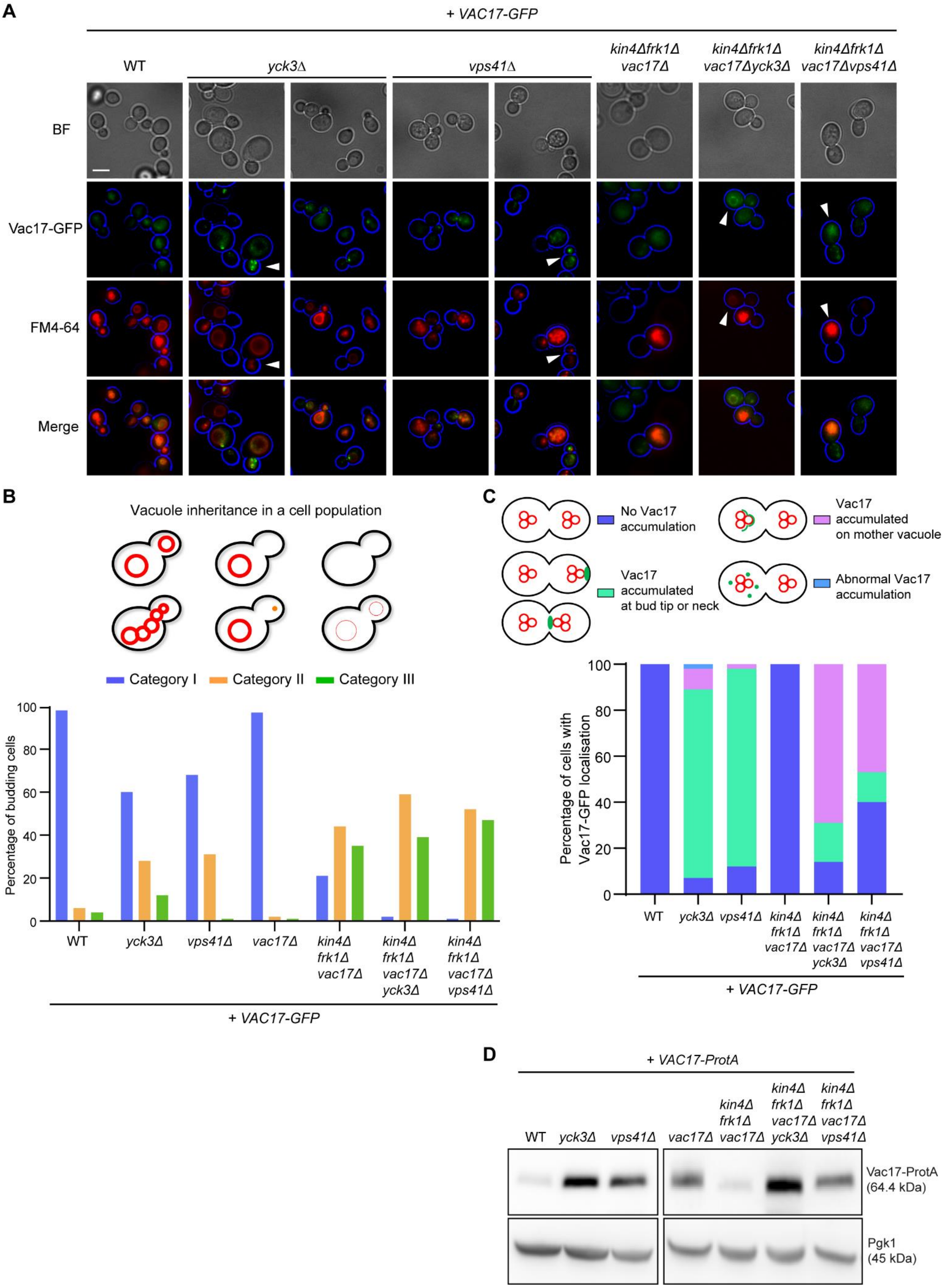

Journal of Cell Science • Supplementary information

Fig. S7. Additional deletion of either *YCK3* or *VPS41* does not rescue the inheritance defect in *kin4Δfrk1Δ* cells. (A) Vac17-GFP accumulation is increased on the vacuolar membrane (indicated by white arrowheads) in cells lacking either *YCK3* or *VPS41*. Cells expressing Vac17-GFP (green) and pulse-chased with FM4-64 (red) were imaged by epifluorescence microscopy. Cell circumference is highlighted in blue. Scale bar: 5 µm. (B) A minimum of 100 cells per strain in A were analysed to quantify the vacuole inheritance in a cell population. Category I, bright FM4-64 staining of vacuoles in mother cells and buds; category II, bright FM4-64 staining in mother cells but not in buds; category III, cells lack bright FM4-64 staining altogether. (C) A minimum of 100 cells were analysed for Vac17-GFP localisation for the strains described in (A). (D) Western blot analysis for Vac17-ProtA steady-state levels in *YCK3* and *VPS41* deficient WT and *kin4Δfrk1Δ* strains. Cells were transformed with a plasmid encoding Vac17-ProtA under the control of the *VAC17* promoter. Cell extracts were analysed by western blotting.

Journal of Cell Science • Supplementary information

Table S1. Yeast strains used in this study.

Strain and genotype	Reference
BY4741 MATa *his3Δ1 leu2Δ0 met15Δ0 ura3Δ0*	EUROSCARF
BY4742 MATα *his3Δ1 leu2Δ0 lys2Δ0 ura3Δ0*	EUROSCARF
BY4742 *vac17Δ::kanMX4*	EUROSCARF
BY4741 *kin4Δ::kanMX4*	EUROSCARF
BY4741 *frk1Δ::kanMX4*	EUROSCARF
BY4742 *frk1Δ::kanMX4 kin4::hphMX4*	(Ekal et al., 2023)
BY4741 *dma1Δ::hphMX4 dma2Δ::kanMX4*	EUROSCARF
BY4741 *bfa1Δ::kanMX4*	EUROSCARF
BY4741 *elm1Δ::kanMX4*	EUROSCARF
BY4741 *rts1Δ::kanMX4*	EUROSCARF
BY4741 *bub2Δ::kanMX4*	EUROSCARF
BY4742 *frk1Δ::kanMX4 kin4::hphMX4 vac17Δ::natMX6*	This study
BY4741 *MYO2::MYO2-GFP-his3MX6*	This study
BY4741 *frk1Δ::kanMX4 kin4Δ::hphMX4 MYO2::MYO2-GFP-his3MX6*	This study
BY4741 *frk1Δ::kanMX4 kin4::hphMX4 cla4Δ::his3MX6*	This study
BY4741 *frk1Δ::kanMX4 kin4::hphMX4 ste20Δ::his3MX6*	This study
BY4741 *frk1Δ::kanMX4 kin4::hphMX4 skm1Δ::his3MX6*	This study
BY4741 *frk1Δ::kanMX4 kin4::hphMX4 dma1Δ::his3MX6*	This study
BY4741 *dma1Δ::kanMX4*	EUROSCARF
BY4742 *myo2Δ::KanMX4, pRS416-MYO2 (URA3)*	(Fagarasanu et al., 2009)
BY4742 *myo2Δ::KanMX4 frk1Δ::natMX6 kin4Δ::hphMX6, pRS416-MYO2 (URA3)*	This study
BY4742 *myo2Δ::KanMX4, pRS413-myo2-D1297N (HIS3)*	This study
BY4742 *myo2Δ::KanMX4 frk1Δ::natMX6 kin4Δ::hphMX6, pRS413-myo2-D1297N (HIS3)*	This study
PJ69-4A MATa, *trp1-901, leu2-3, ura3-52, his3-Δ200, Δgal4, Δgal80, LYS2::GAL1-HIS3, GAL2-ADE2, met::GAL7-lacZ*	(James et al., 1996)
PJ69-4A MATα, *trp1-901, leu2-3, ura3-52, his3-Δ200, Δgal4, Δgal80, LYS2::GAL1-HIS3, GAL2-ADE2, met::GAL7-lacZ*	(James et al., 1996)
BY4741 *yck3Δ::kanMX4*	EUROSCARF
BY4741 *vps41Δ::kanMX4*	EUROSCARF

BY4742 *frk1Δ::kanMX4 kin4::hphMX4 vac17Δ::natMX6 yck3Δ::his3MX6*	This study
BY4742 *frk1Δ::kanMX4 kin4::hphMX4 vac17Δ::natMX6 vps41Δ::his3MX6*	This study
BY4742 *CLA4::natMX6-TEF2-CLA4*	(Yofe et al., 2016)

Journal of Cell Science • Supplementary information

Table S2. A list of plasmids used in this study.

Plasmid Name	Vector backbone	Promoter	Insert	UniProt ID	Source
pEW318	Ycplac33	-	-		Lab stock
pEW319	Ycplac111	-	-		Lab stock
pLE108	Ycplac33	*VAC17*	*VAC17-2xProtA*	P25591	This study
pLE50	Ycplac33	*GAL1*	*KIN4*	Q01919	(Ekal et al., 2023)
pLE52	Ycplac111	*GAL1*	*KIN4*	Q01919	(Ekal et al., 2023)
pLE51	Ycplac33	*GAL1*	*FRK1*	Q03002	(Ekal et al., 2023)
pLE53	Ycplac111	*GAL1*	*FRK1*	Q03002	(Ekal et al., 2023)
pLE54	Ycplac111	*VAC17*	*VAC17-GFP*	P25591	This study
pLE79	Ycplac111	*VAC17*	*VAC17-S222A-GFP*	P25591	This study
pLE80	Ycplac111	*VAC17*	*VAC17-T240A-GFP*	P25591	This study
pLE114	Ycplac33	*VAC17*	*VAC17-TS222A-2xProtA*	P25591	This study
pLE121	Ycplac33	*VAC17*	*VAC17-T240A-2xProtA*	P25591	This study
pLE58	Ycplac111	*KIN4*	*KIN4*	Q01919	(Ekal et al., 2023)
pLE60	Ycplac111	*KIN4*	*KIN4-T209A*	Q01919	(Ekal et al., 2023)
pLE59	Ycplac111	*FRK1*	*FRK1*	Q03002	This study
pLE61	Ycplac111	*FRK1*	*FRK1-T209A*	Q03002	This study
pAA42	Ycplac33	*VAC17*	*VAC17-R135E-2xProtA*	P25591	This study
pAA44	Ycplac33	*VAC17*	*VAC17-K138D-2xProtA*	P25591	This study
pAA74	Ycplac33	*VAC17*	*VAC17-R142E-2xProtA*	P25591	This study
pAA40	Ycplac33	*VAC17*	*VAC17-R135E,K138D-2xProtA*	P25591	This study
pAA75	Ycplac33	*VAC17*	*VAC17-R135E,R142E-2xProtA*	P25591	This study
pAA76	Ycplac33	*VAC17*	*VAC17-K138D,R142E-2xProtA*	P25591	This study
pAA39	Ycplac33	*VAC17*	*VAC17-R135,K138D-GFP*	P25591	This study
pAA50	Ycplac33	*VAC17*	*VAC17-R135,K138D,S222A-2xProtA*	P25591	This study
pAA51	Ycplac33	*VAC17*	*VAC17-R135,K138D,T240A-2xProtA*	P25591	This study
	pVT102u	*ADH1*	*VAC17-GFP*		(Yau et al., 2014)
		CUP1	*Myc-Ubiquitin*		(Yau et al., 2014)
	pGAD-C1	*GAL4*	*MYO2-CBD (1131-1574 aa)*	P19524	(Eves et al., 2012)
	pGAD-C1	*GAL4*	*myo2-CBD E1293K*	P19524	(Eves et al., 2012)
	pGAD-C1	*GAL4*	*myo2-CBD D1296N*	P19524	(Eves et al., 2012)
	pGAD-C1	*GAL4*	*myo2-CBD D1297N*	P19524	(Eves et al., 2012)
	pGAD-C1	*GAL4*	*myo2-CBD N1304S*	P19524	(Eves et al., 2012)
	pGBD-C1	*GAL4*	*VAC17*	P25591	(Eves et al., 2012)
pAA60	pGBD-C1	*GAL4*	*vac17-R135E*	P25591	This study
pAA61	pGBD-C1	*GAL4*	*vac17-K138D*	P25591	This study
pAA62	pGBD-C1	*GAL4*	*vac17-R142E*	P25591	This study
pAA63	pGBD-C1	*GAL4*	*vac17-R135E,K138D*	P25591	This study
pAA64	pGBD-C1	*GAL4*	*vac17-K138D,R142E*	P25591	This study
pAA65	pGBD-C1	*GAL4*	*vac17-R135E,R142E*	P25591	This study

Journal of Cell Science • Supplementary information

pLE107	Ycplac33	*INP2*	*INP2-2xProtA*	Q03824	(Ekal et al., 2023)
pLE49	Ycplac111	*INP2*	*INP2-GFP*	Q03824	(Ekal et al., 2023)
pLE56	Ycplac111	*VPH1*	*VPH1-GFP*	P32563	This study
pAUL28	Ycplac33	*HIS3*	*mKate2-PTS1*		Lab stock
pAUL3	Ycplac33	*HIS3*	*mNG-PTS1*		Lab stock
pLE102	pRS416	*RRP4*	*GFP-TUB1*	P09733	This study

REFERENCES

Ekal, L., A.M.S. Alqahtani, M. Schuldiner, E. Zalckvar, E.H. Hettema, and K.R. Ayscough. 2023. Spindle Position Checkpoint Kinase Kin4 Regulates Organelle Transport in Saccharomyces cerevisiae. *Biomolecules*. 13.

Eves, P.T., Y. Jin, M. Brunner, and L.S. Weisman. 2012. Overlap of cargo binding sites on myosin V coordinates the inheritance of diverse cargoes. *J Cell Biol*. 198:69-85.

Fagarasanu, A., F.D. Mast, B. Knoblach, Y. Jin, M.J. Brunner, M.R. Logan, J.N. Glover, G.A. Eitzen, J.D. Aitchison, L.S. Weisman, and R.A. Rachubinski. 2009. Myosin-driven peroxisome partitioning in S. cerevisiae. *J Cell Biol*. 186:541-554.

James, P., J. Halladay, and E.A. Craig. 1996. Genomic libraries and a host strain designed for highly efficient two-hybrid selection in yeast. *Genetics*. 144:1425-1436.

Liu, Y., L. Li, C. Yu, F. Zeng, F. Niu, and Z. Wei. 2022. Cargo Recognition Mechanisms of Yeast Myo2 Revealed by AlphaFold2-Powered Protein Complex Prediction. *Biomolecules*. 12.

Yau, R.G., Y. Peng, R.R. Valiathan, S.R. Birkeland, T.E. Wilson, and L.S. Weisman. 2014. Release from myosin V via regulated recruitment of an E3 ubiquitin ligase controls organelle localization. *Dev Cell*. 28:520-533.

Yofe, I., U. Weill, M. Meurer, S. Chuartzman, E. Zalckvar, O. Goldman, S. Ben-Dor, C. Schutze, N. Wiedemann, M. Knop, A. Khmelinskii, and M. Schuldiner. 2016. One library to make them all: streamlining the creation of yeast libraries via a SWAp-Tag strategy. *Nat Methods*. 13:371-378.

Journal of Cell Science • Supplementary information

Figure 2A,B

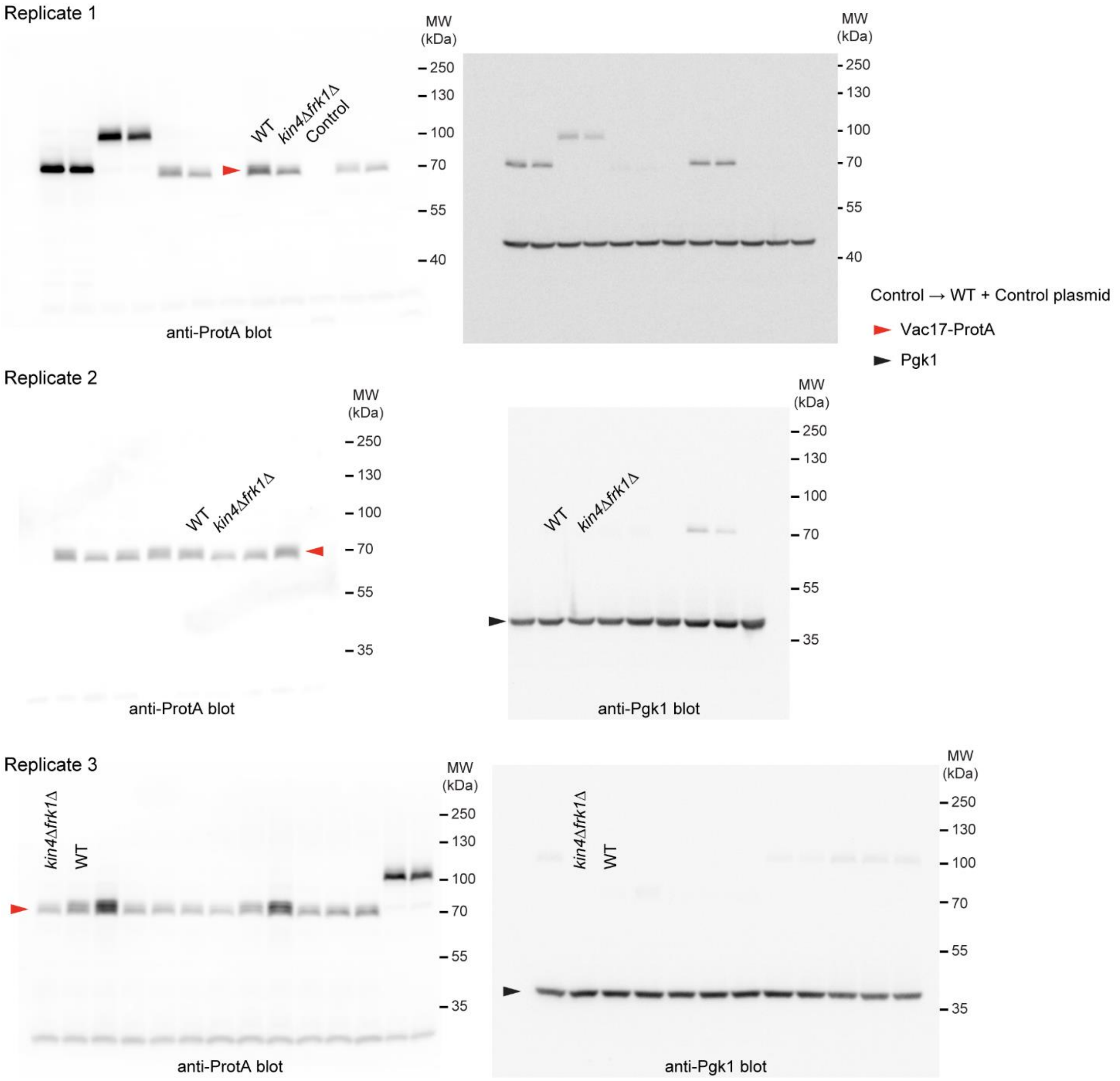

Figure 2C

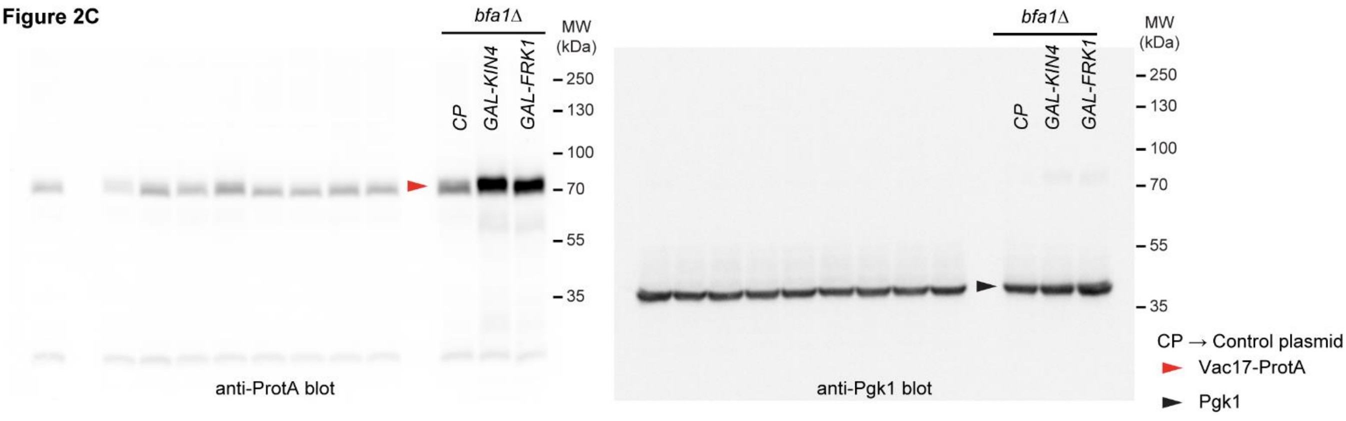

Journal of Cell Science • Supplementary information

Figure2F_G

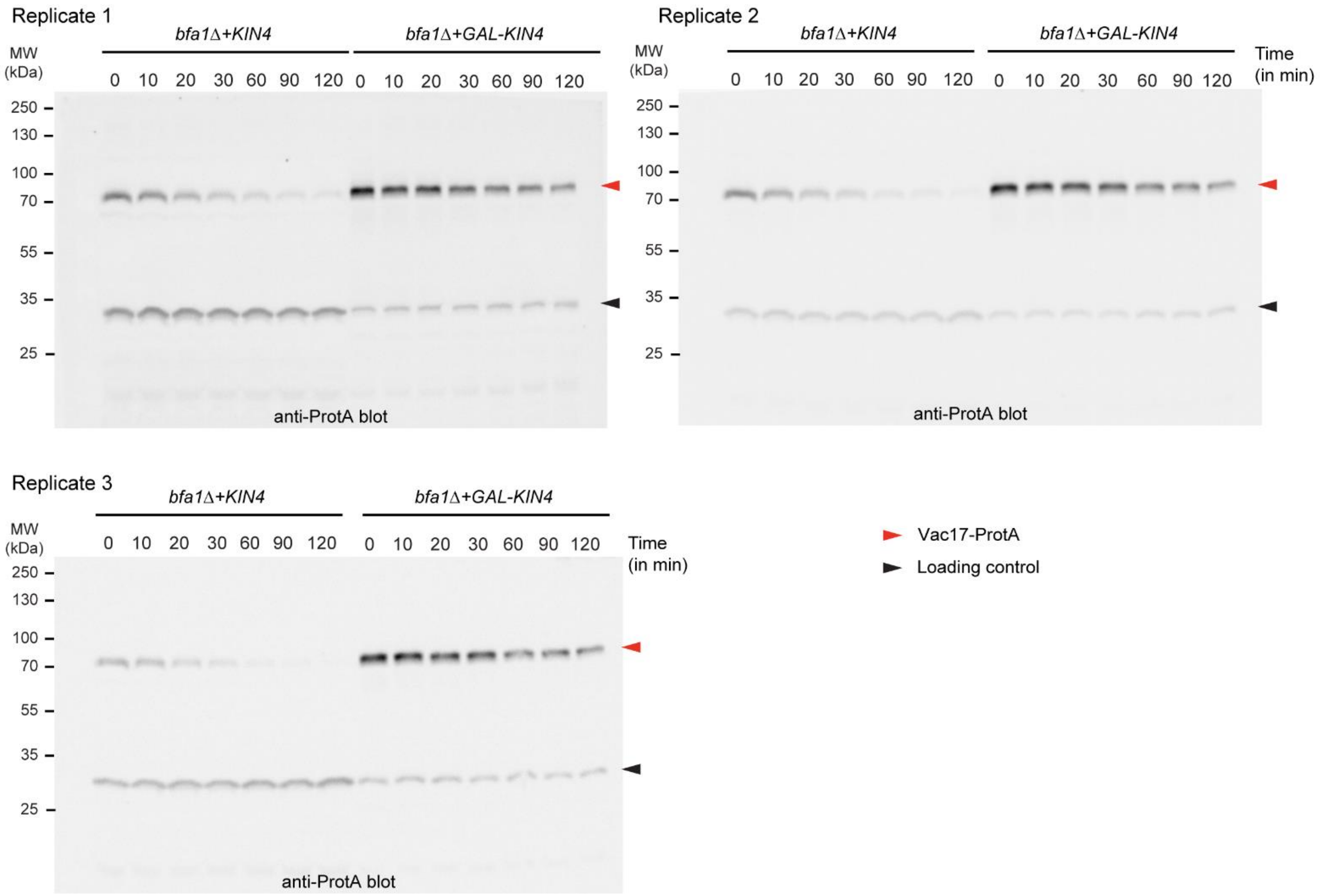

Journal of Cell Science • Supplementary information

Figure2H_I

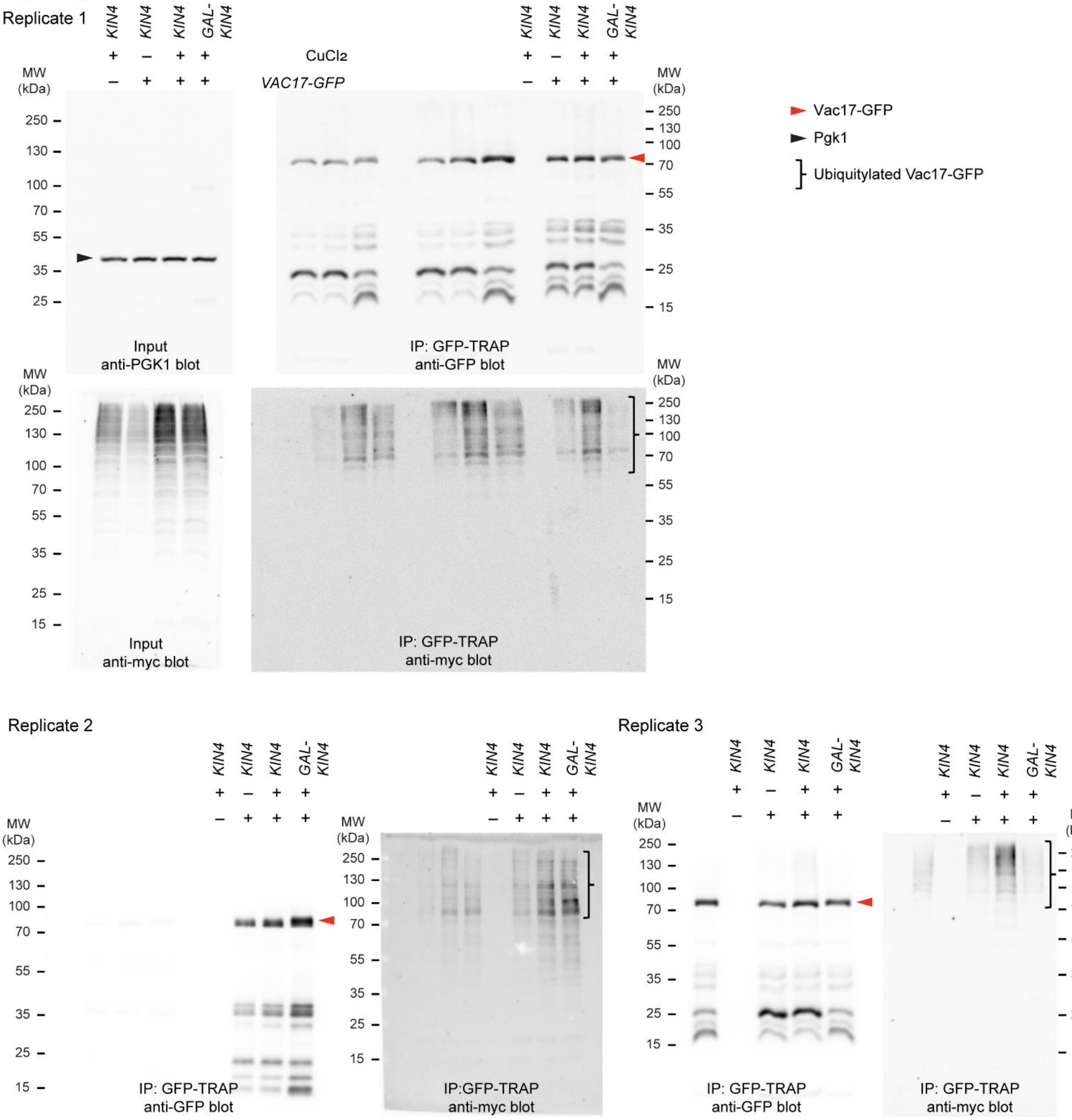

Journal of Cell Science • Supplementary information

Figure 3C_E

Figure 3C

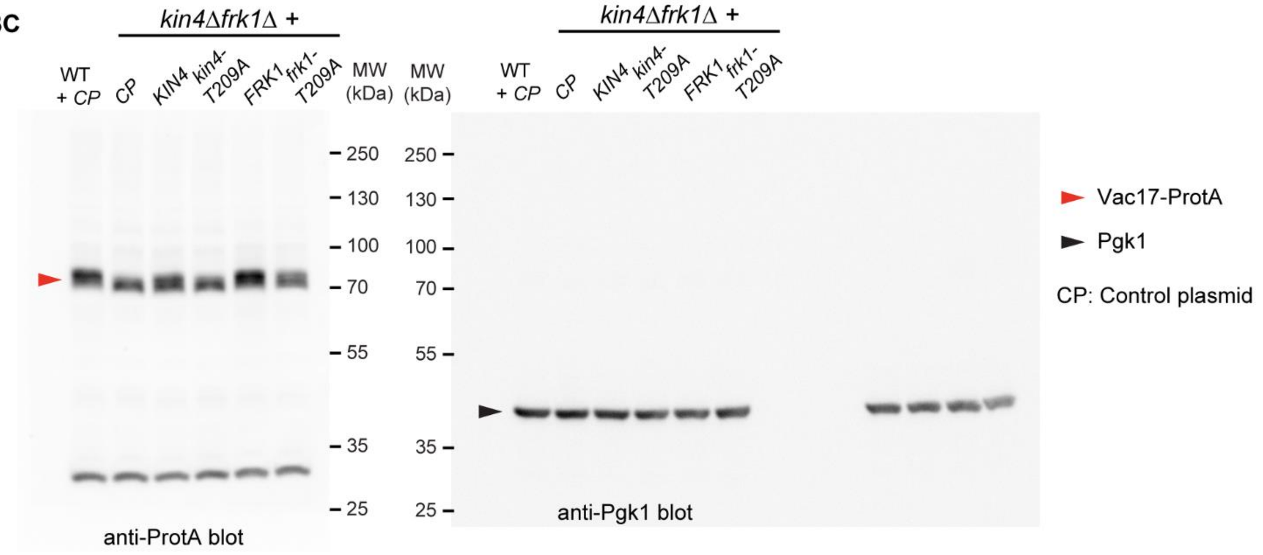

Figure 3E

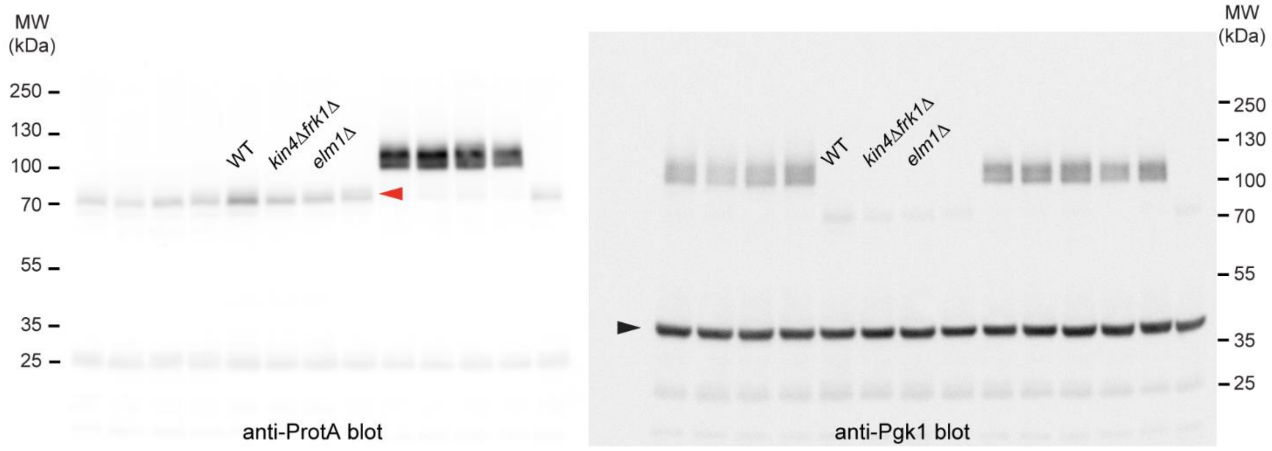

Journal of Cell Science • Supplementary information

Figure 4A

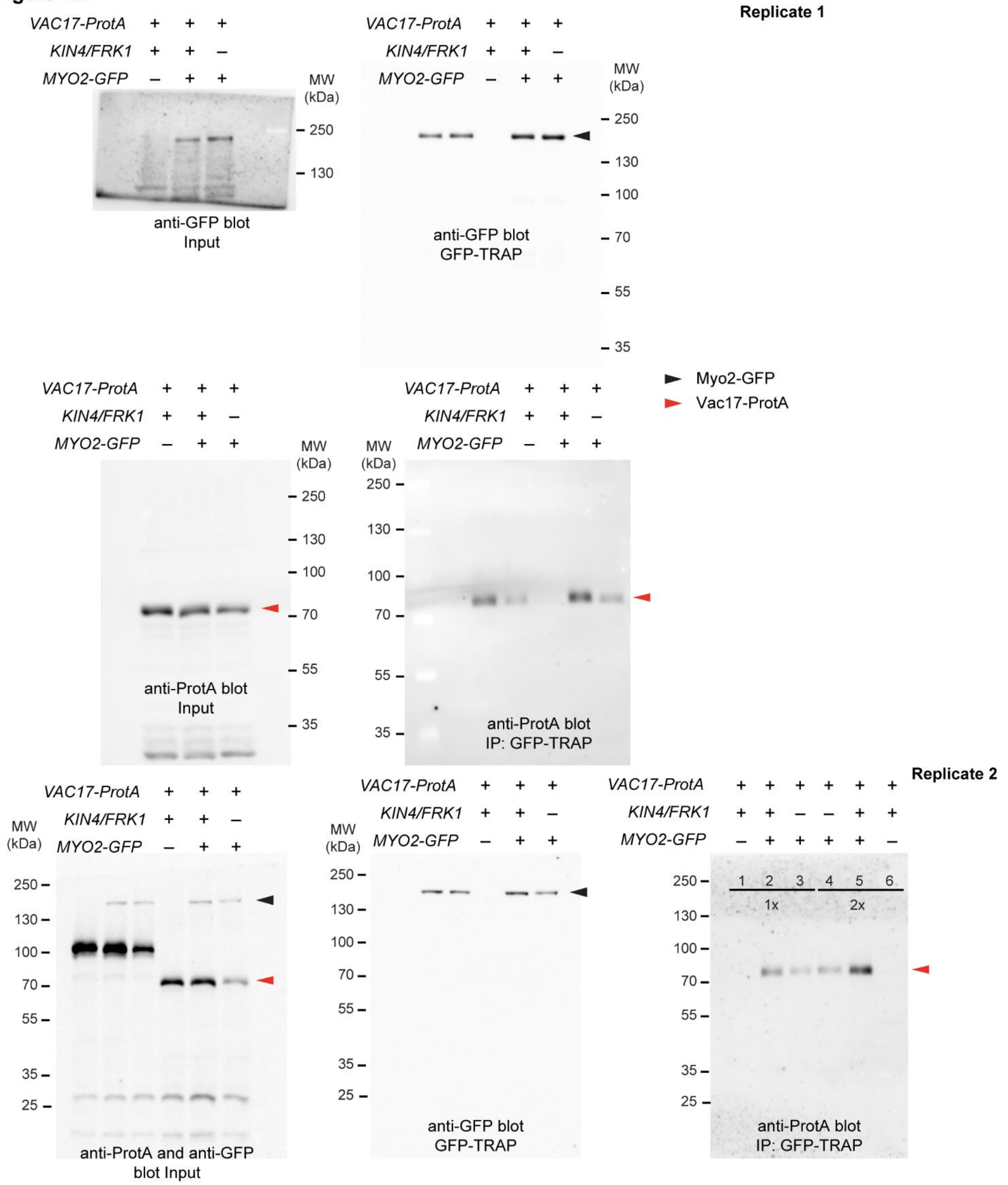

Journal of Cell Science • Supplementary information

Figure 5C

Replicate 1

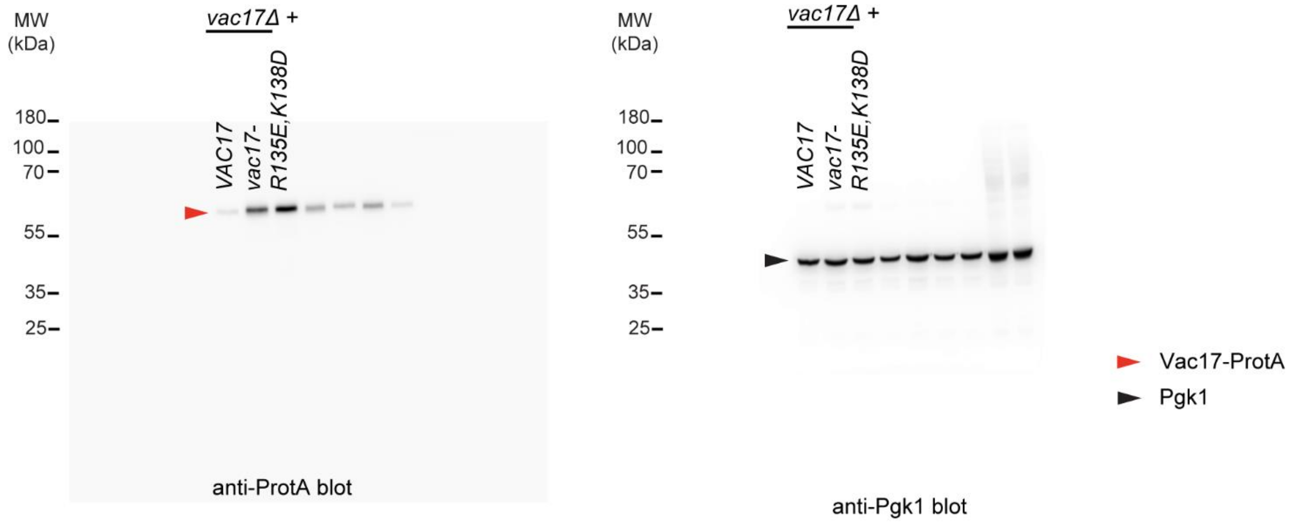

Replicate 2

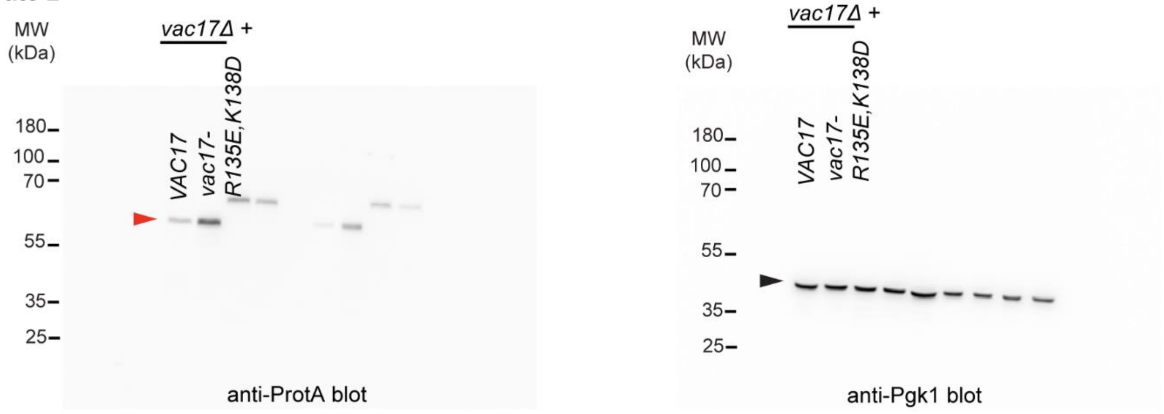

Replicate 3

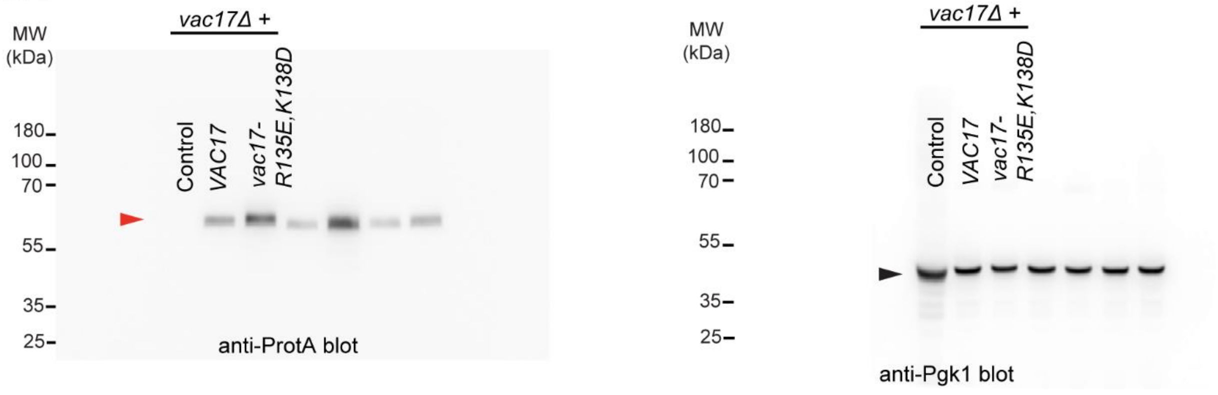

Journal of Cell Science • Supplementary information

Figure 5E

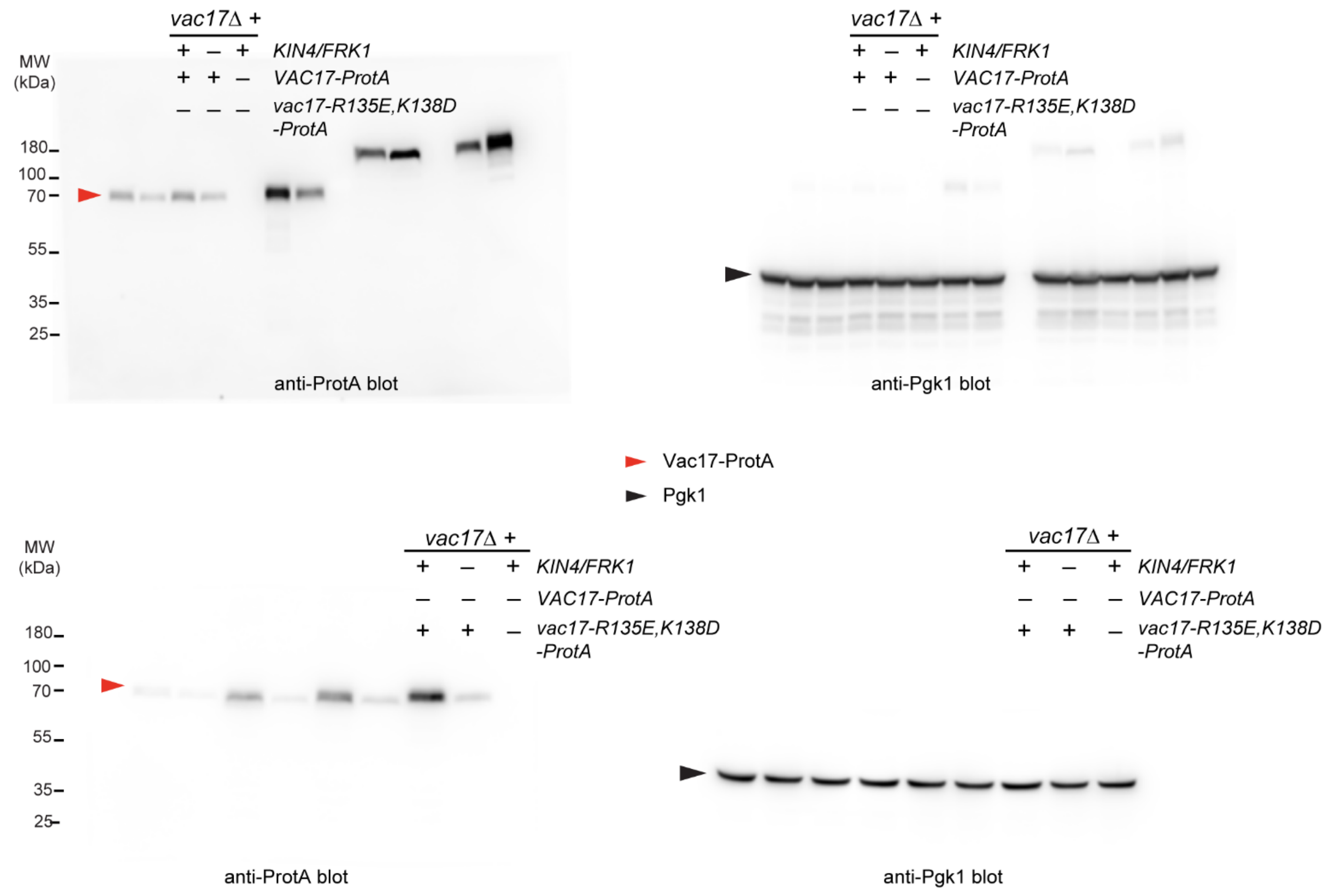

Journal of Cell Science • Supplementary information

Figure 6G

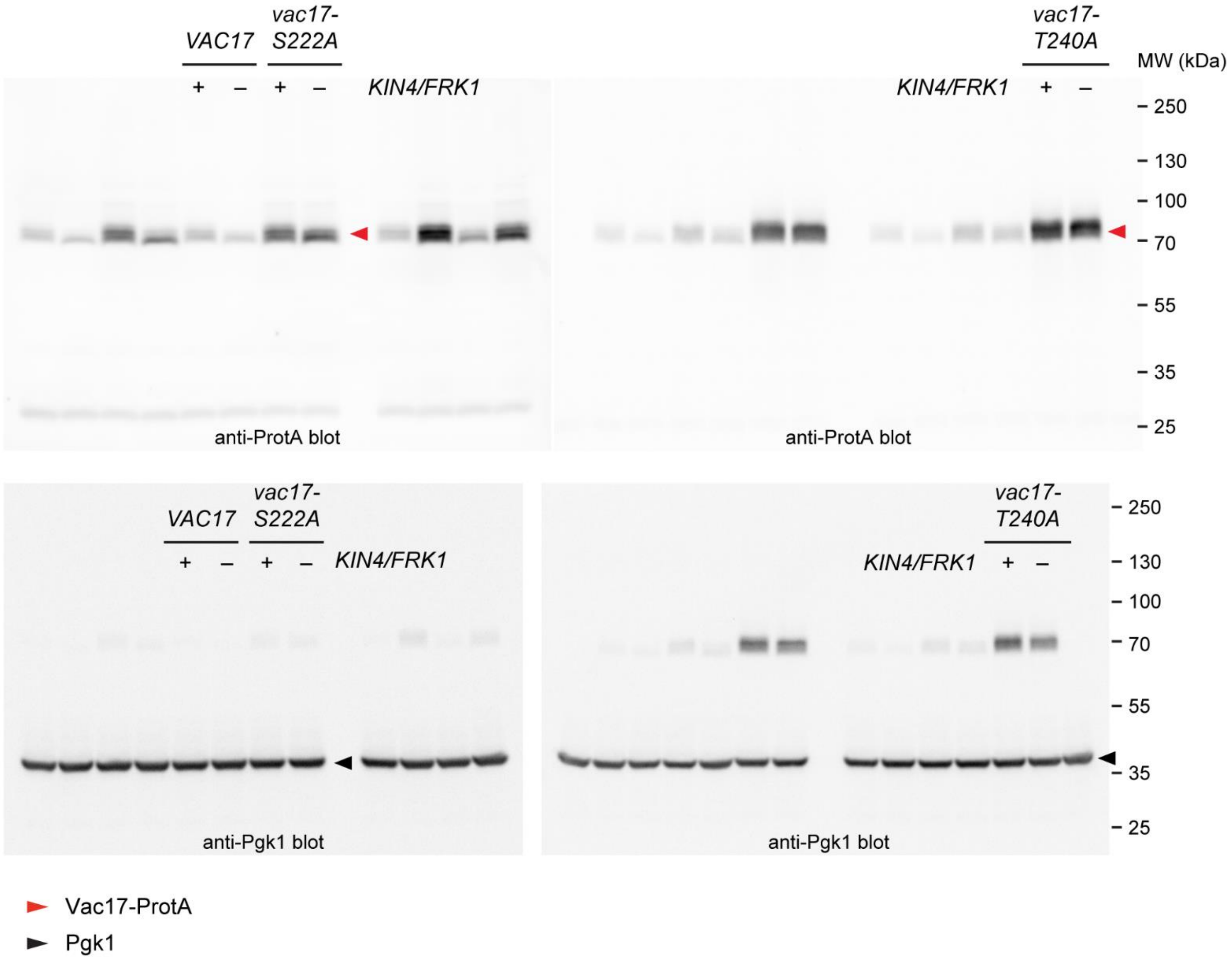

Journal of Cell Science • Supplementary information

Figure 6H

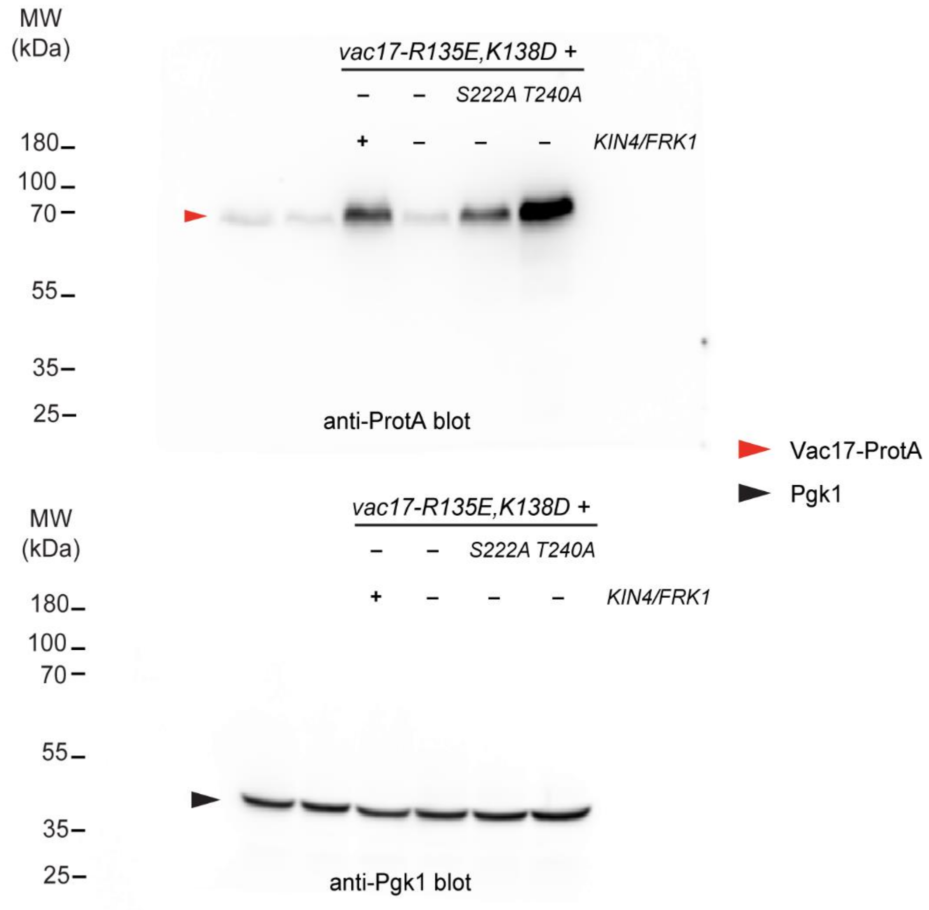

Figure 7A

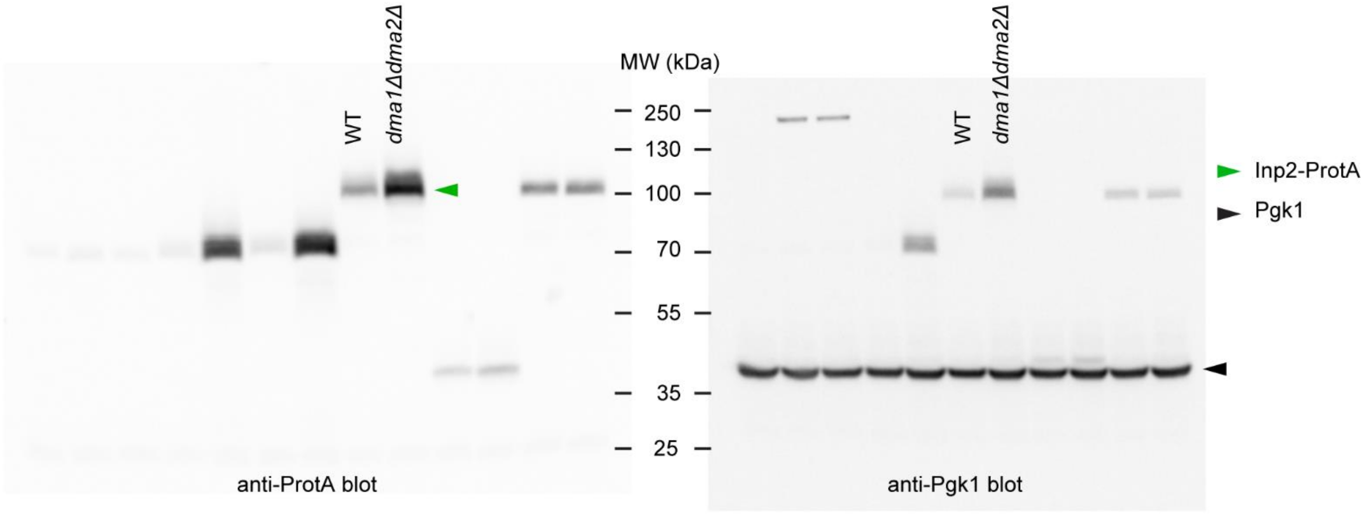

Journal of Cell Science • Supplementary information

Figure 7F

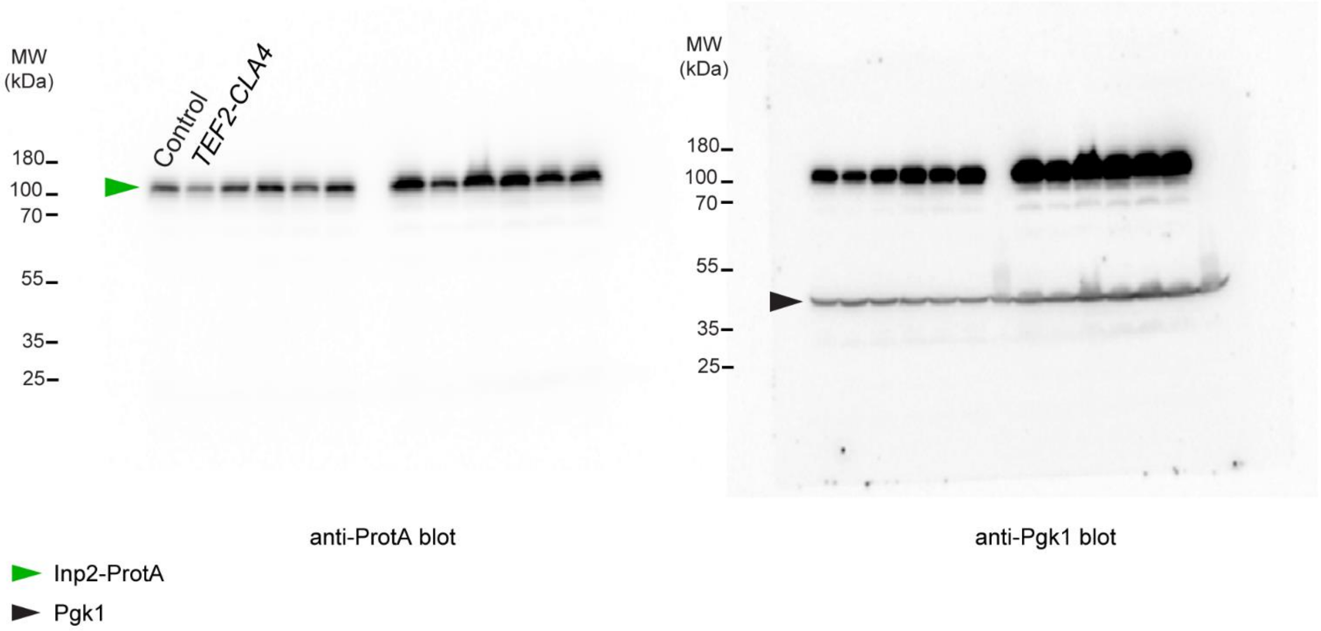

Figure 7G

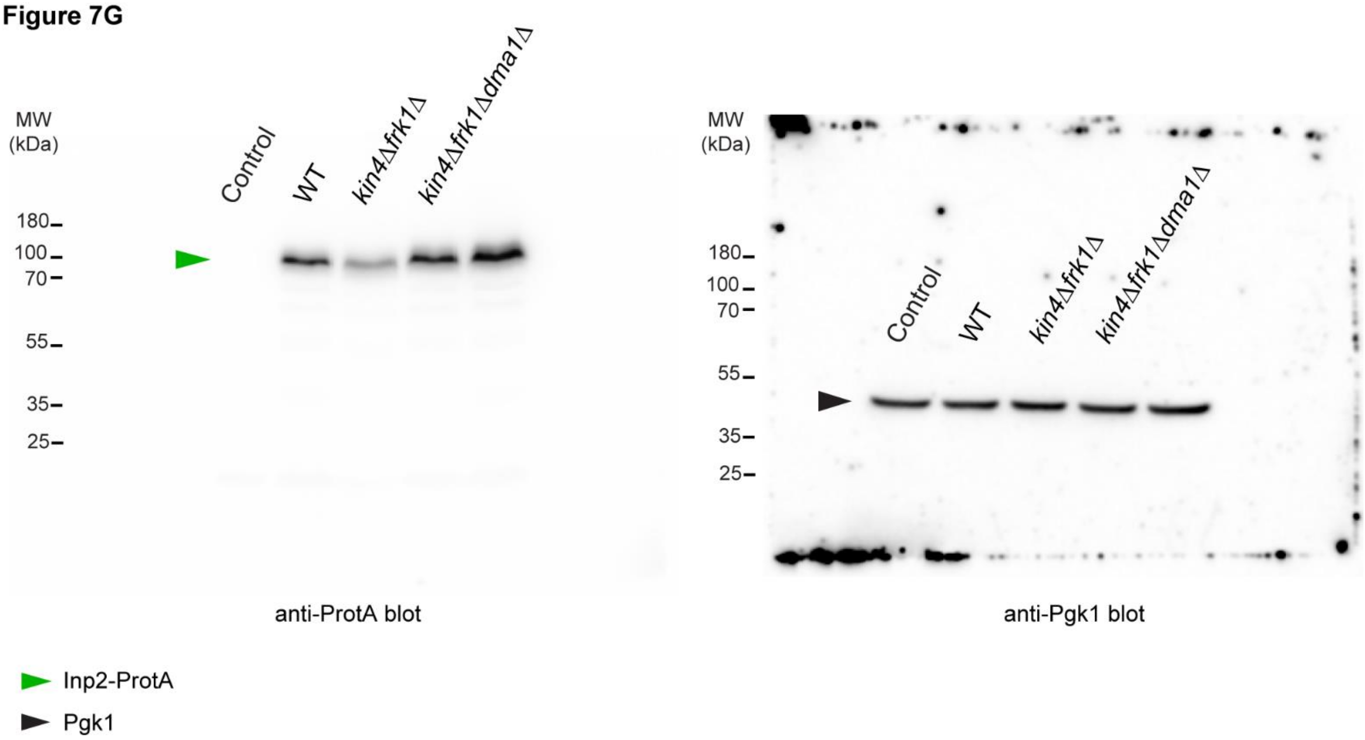

Figure S2B

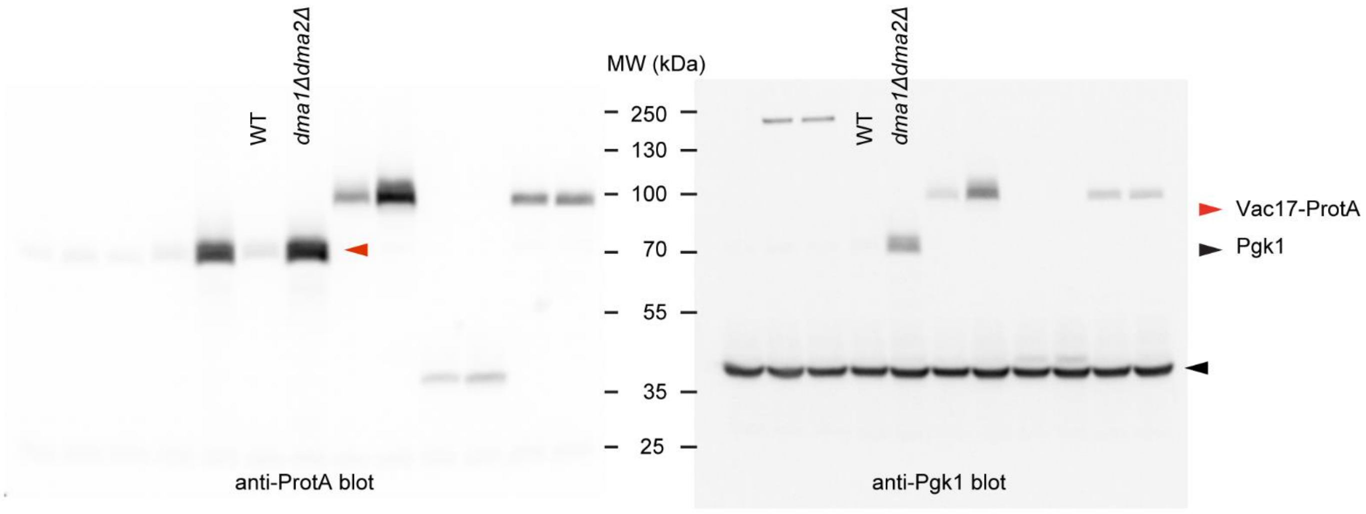

Figure S4D

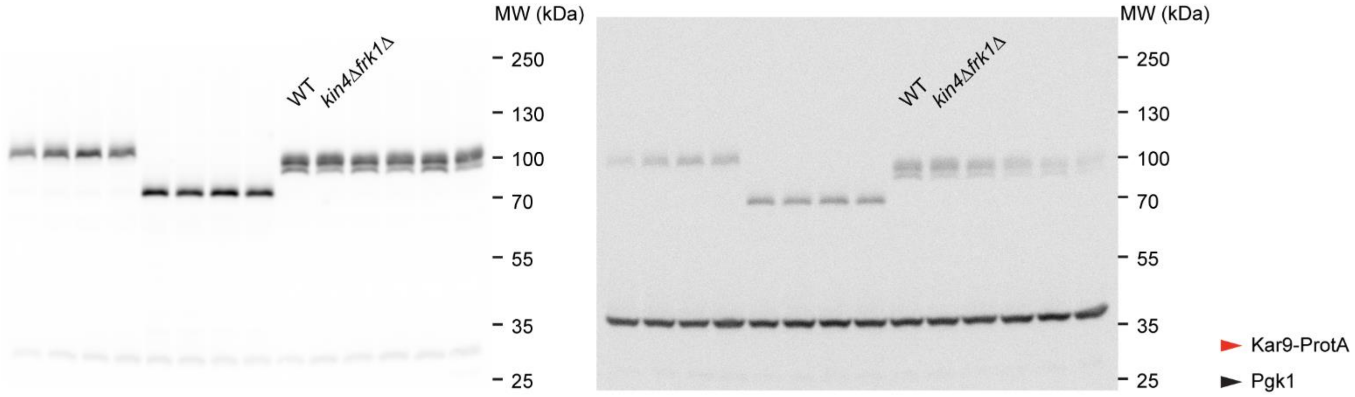

Journal of Cell Science • Supplementary information

FigureS5 A

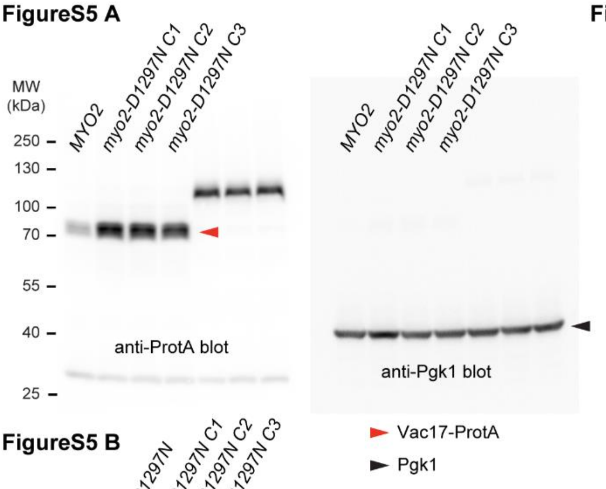

FigureS5 B

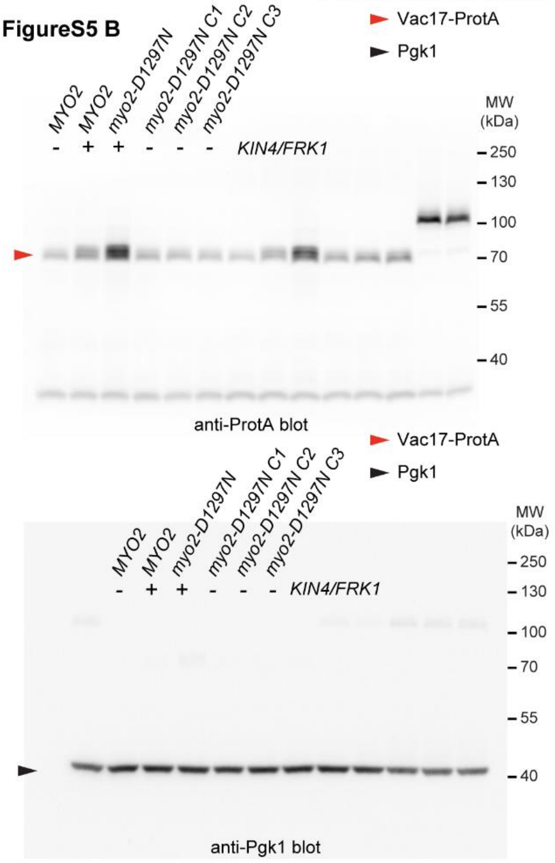

FigureS5 C

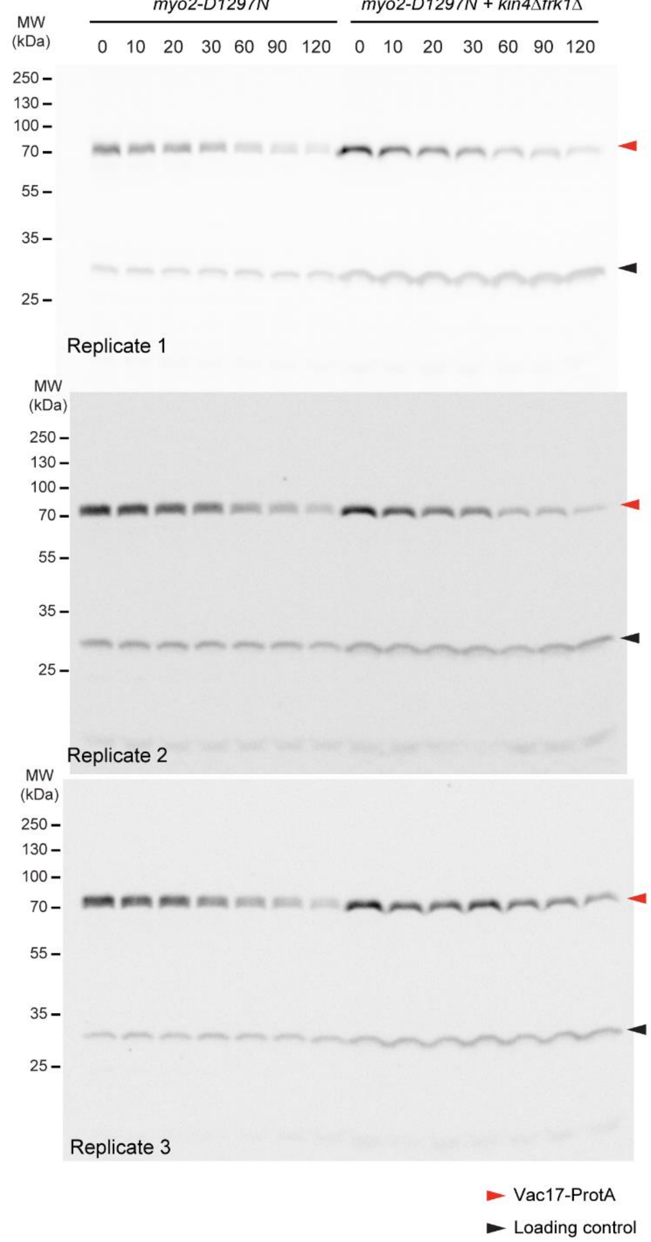

FigureS5 E

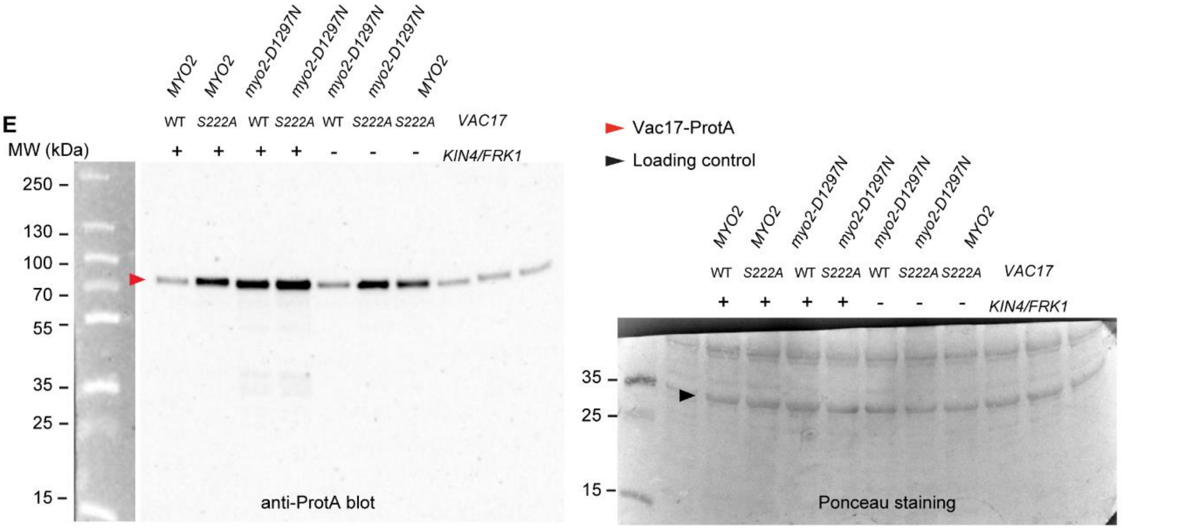

Journal of Cell Science • Supplementary information

Figure S6E

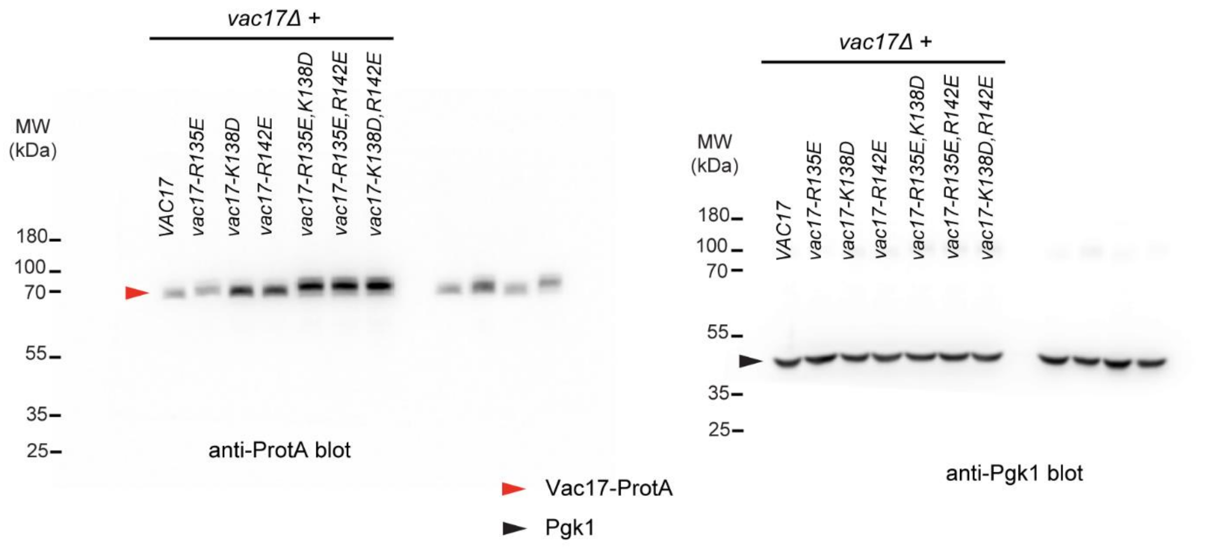

Figure S7C

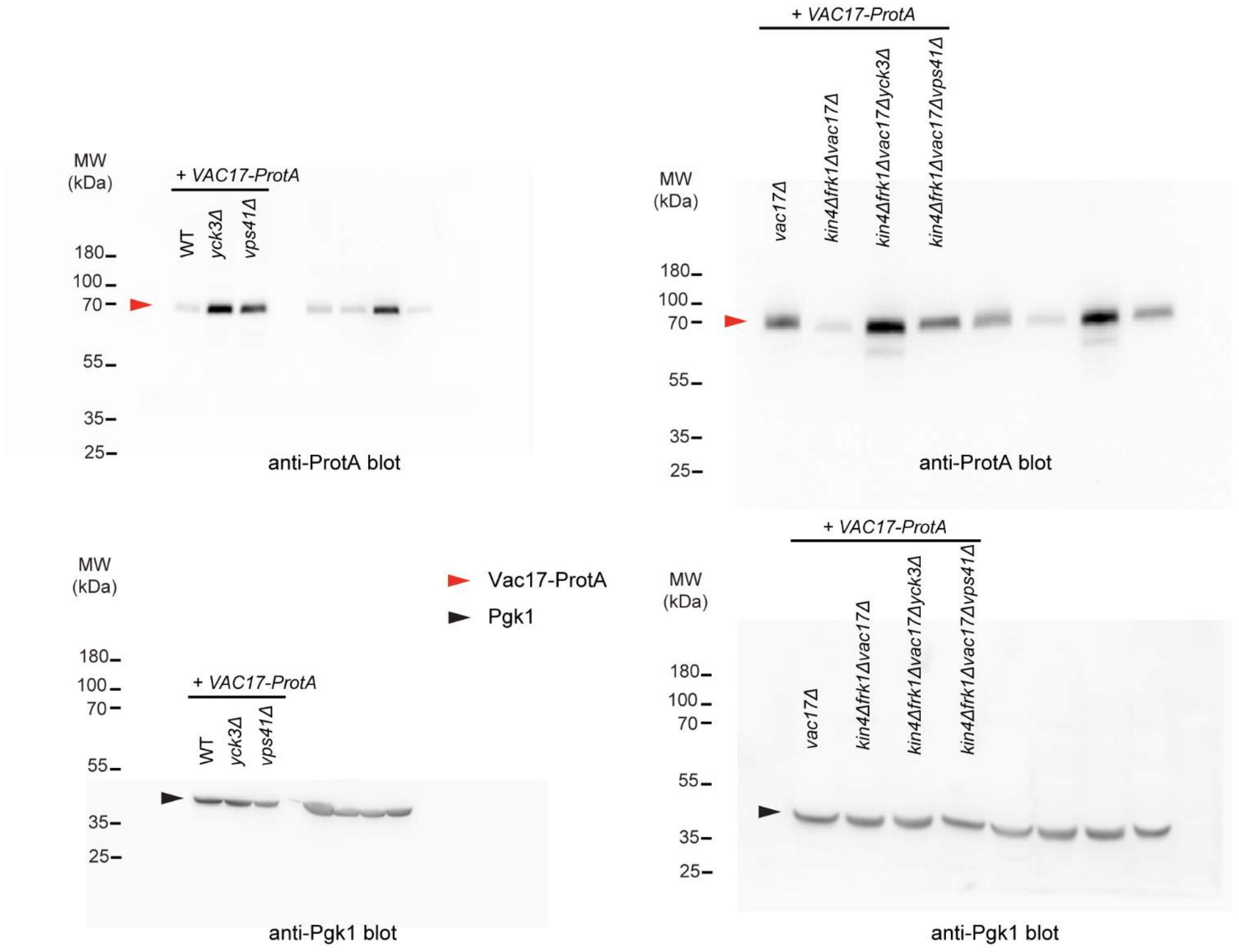

Fig. S8. Blot transparency.

Journal of Cell Science • Supplementary information
