# Supplementary material for: Spatiotemporal regulation of organelle transport by spindle position checkpoint kinase Kin4
Source: J Cell Sci. 2024 Nov 13;137(21):jcs261948. doi: 10.1242/jcs.261948 (PMC11586526; doi:10.1242/jcs.261948)
Supplement: Supplementary file 1 [file joces-137-261948_review_history.pdf]

Journal of Cell Science

Spatiotemporal regulation of organelle transport by spindle position checkpoint kinase Kin4

Lakhan Ekal, Abdulaziz M. S. Alqahtani, Kathryn R. Ayscough and Ewald H. Hettema
DOI: 10.1242/jcs.261948

Editor: Renata Basto

Review timeline
Original submission: 13 January 2024
Editorial decision: 12 March 2024
First revision received: 23 July 2024
Editorial decision: 19 August 2024
Second revision received: 4 September 2024
Accepted: 16 September 2024

Original submission

First decision letter

MS ID#: JOCES/2024/261948

MS TITLE: Spatiotemporal regulation of organelle transport by spindle position checkpoint kinase Kin4

AUTHORS: Lakhan Ekal, Abdulaziz M. S. Alqahtani, Kathryn R. Ayscough, and Ewald H. Hettema

We have now reached a decision on the above manuscript.

To see the reviewers' reports and a copy of this decision letter, please go to: https://submit-jcs.biologists.org and click on the 'Manuscripts with Decisions' queue in the Author Area. (Corresponding author only has access to reviews.)

As you will see, the reviewers gave favourable reports but raised some critical points that will require amendments to your manuscript. I hope that you will be able to carry these out because I would like to be able to accept your paper, depending on further comments from reviewers.

Please ensure that you clearly highlight all changes made in the revised manuscript. Please avoid using 'Tracked changes' in Word files as these are lost in PDF conversion.

I should be grateful if you would also provide a point-by-point response detailing how you have dealt with the points raised by the reviewers in the 'Response to Reviewers' box. Please attend to all of the reviewers' comments. If you do not agree with any of their criticisms or suggestions please explain clearly why this is so.

Reviewer 1

Advance summary and potential significance to field

The study builds upon previous work by the same group, demonstrating that vacuole inheritance in budding yeast is influenced by the kinases Kin4 and Frk1. Here, the authors present evidence

© 2024. Published by The Company of Biologists under the terms of the Creative Commons Attribution License (https://creativecommons.org/licenses/by/4.0/).

indicating that Kin4 and Frk1, in a kinase-dependent manner, regulate vacuole inheritance by protecting Vac17 from degradation, which is mediated by the kinase Cla4 and the E3-ubiquitin ligase Dma1/2. The opposing functions of Kin4 and Frk1 in preventing Cla4-Dam1/2-dependent protein degradation, and consequently organelle transport termination, extend beyond vacuoles. Similar regulatory mechanisms were observed for peroxisomes (Inp2), previously reported by the authors to also be regulated in a Kin4 and Frk1-dependent manner. Notably, Kin4 and Frk1's role in vacuole inheritance is independent of their function in cell cycle regulation as part of the spindle position checkpoint (SPOC), thereby expanding Kin4's role beyond cell cycle regulation.
Based on the localization of Kin4 and Cla4, the authors propose a zone-model similar to the one proposed for SPOC function. In this model, Cla4 in the bud promotes vacuolar transport termination (VTT) in large budded cells. At this point in the cell cycle, Kin4 is preferentially associated with the mother cell cortex, thus unable to inhibit Cla4. However, in small budded cells, Kin4 accumulates in the bud, preventing premature VTT. Together, this study sheds light on the molecular mechanisms governing organelle transport in cells.

Comments for the author

The manuscript presents a solid piece of work supported by well-performed experiments and data-driven conclusions. However, there are minor points requiring clarification before publication.
Specific points:
1. In Fig 4A, the authors investigate whether Kin4 and Frk1 influence the Myo2-Vac17 interaction via immunoprecipitation and conclude that Kin4 and Frk1 do not influence Vac17-Myo2 interaction. However, Vac17 co-immunoprecipitates with Myo2 at weaker levels than in WT cells. The authors argue that lower Vac17 levels in kin4Δfrk1Δ cells could account for reduced Vac17 co-IPed with Myo2. However, it is difficult to exclude from this experiment the possibility that Kin4 and Frk1 may also influence Vac17-Myo2 binding to certain extent. Ideally, the authors should immunoprecipitate Vac17 and quantify the ratio of co-IPed Myo2 in the Vac17-Myo2 complex in WT and kin4Δfrk1Δ cells.
2. In Fig. 4C, the transport of the pre-vacuolar membrane (as indicated in lines 334-336) is not clear in the images. Please provide one representative cell in which the transport is evident, albeit short-lived.
3. Fig S1A provides valuable insight into the components analyzed in this study and helps in understanding the experimental logic. I suggest moving it to the main figure.

Reviewer 2

Advance summary and potential significance to field

Cells have developed molecular mechanisms to ensure that they maintain the metabolic benefits of having membrane-enclosed organelles while equitably sharing these benefits with their daughter cells at the time of cell division.
The budding yeast Saccharomyces cerevisiae is an excellent model system with which to study molecular components underlying organelle inheritance because of its polarized cell division. While much has been learned about the molecular motors and their receptors on the various membrane-enclosed organelles that are inherited by yeast at cell division, we know much less about the molecules and their actions that control the spatiotemporal regulation of organelle transport.
In this exceptional manuscript, Ekal and colleagues present unequivocal evidence that Vac17, the vacuole (the yeast counterpart of the mammalian lysosome) receptor that recognizes the myosin V motor, Myo2, that moves vacuoles along actin to the growing yeast, is broken down in the bud to ensure proper positioning of vacuoles in the bud. Vac17 breakdown in the bud is controlled by the p21-activated kinase Cla4, which is enriched in the bud, together with the E3-ubiquitin ligase Dma1. The spindle position checkpoint kinase, Kin4, and to a lesser extent its paralogue Frk1, contribute to the successful transport of vacuoles to the bud by preventing premature breakdown of Vac17 by Cla4 and Dnm1 in the mother cell or in small buds. Kin4 and Cla4 act similarly in the regulation of transport of peroxisomes to the bud through their actions on the peroxisomal Myo2 receptor, Inp2. Therefore, as the authors correctly conclude Kin4 acts antagonistically to the actions of Cla4/Dnm1to regulate organelle inheritance with respect to both time and space in the yeast cell.

© 2024. Published by The Company of Biologists under the terms of the Creative Commons Attribution License (https://creativecommons.org/licenses/by/4.0/).

Comments for the author

The results presented in this manuscript are exceptional in their quality and abundance, and unequivocally justify the conclusions of the authors. This reviewer sees no need to add additional findings to the manuscript or to modify the figures as they now are. The authors should address the following minor points in their revision:

1) Line 154. Remove ‘the’ before ‘vacuole’.
2) Line 158. Remove ‘a’ before ‘fresh’.
3) Line 258. ‘exponentially’ not ‘exponential’.
4) Line 261. Add ‘the’ before ‘T209’.
5) Line 296. The reference (Beach et al., 2000) is incorrectly designated. Correct here and in the References.
6) Line 725. ‘vacuole positioning’ not ‘peroxisome positioning’.
7) Title to Figure S5. The results presented in this figure do not show, but only imply, that Vac17 breakdown is Cla4-/Dnm1-dependent. The authors should therefore remove ‘Cla4-/Dnm1-dependent’.
8) Legend to Figure S6, line 6. ‘Mata’ not ‘MatA’.

<u>Reviewer 3</u>

Advance summary and potential significance to field

In this manuscript, Ekal et al build upon their previous work (Ekal et al 2023) on the identification of SPOC kinases Kin4/Frk1 as important regulators of Myo2-specific cargo adaptors during organelle inheritance. That study showed that Kin4/Frk1 are involved in promoting peroxisome transit into emerging buds, by preventing premature degradation of the peroxisome adaptor Inp2 in the mother cell. In the current manuscript, the authors find that Kin4/Frk1 also control the levels of the Myo2 vacuole adaptor, Vac17, and that this control takes place in the mother cell. They provide evidence for how this degradation is prevented.
Previous studies from others had shown that when Myo2 delivers the vacuole and Vac17 to the bud cortex, a PAK kinase, Cla4 phosphorylates Vac17, which recruits an activator of the E3 ligase, Dma1. Then the activated Dma1 ubiquitinates Vac17 which subsequently results in Vac17 degradation and the release of Myo2. According to previous studies, this degradation only occurs in the bud via spatial regulation of Cla4 and Dma1. However, here the authors make a convincing case that in the absence of Kin4/Frk1, Cla4 and Dma1 have access to Vac17 in the mother cells. This premature degradation of Vac17 results in a defect in vacuole inheritance. The authors also show that Kin4/Frk1 similarly stabilizes Inp2 by preventing its degradation by Cla4 and Dma1. Overall, this paper provides a significant advance in current knowledge of Myo2-based organelle transport.
General comments:
Figure 2C. The authors should quantify their western blots so that experiments can be compared. For example, does the bfa1 KO mutation alone lower Vac17 levels?
Figure S4C. The authors should include a quantification of the severity of spindle alignment. Based on the images shown, it appears that kin4/frk1 null cells may also have partial defect.
Line 317-319/Line338-340: The authors conclude that Myo2 and Vac17 interactions are not affected. However, their data would not reveal if binding was partially impaired. A simple pull-down is not sufficient because differences in affinity would not be detected in this assay. The authors should explain that the ability of Vac17 to bind Myo2 might also be impaired. For example, it is possible that kin4/frk1 directly or indirectly impact amino acids within the Myo2-Vac17 binding interface. Along similar lines, the defect in the formation of segregation structures may also be due to a partial defect in Myo2-Vac17 interactions.
Figure 4B/Line 327-331: The authors might want to comment more about the transient nature of the segregation structures in the kin4/frk1 mutant. This could be due to less complexes of Myo2-Vac17 due to less Vac17 levels. In addition, a potential partial defect in Myo2 association with Vac17 may make the complexes less stable.
Figure S5E. The authors are missing some important controls. What are the levels of Vac17 in an otherwise wildtype mutant expressing vac17-S222A, also what are the levels of Vac17-S222A in a myo2-D1297N mutant with wildtype Kin4/Frk1.

© 2024. Published by The Company of Biologists under the terms of the Creative Commons Attribution License (https://creativecommons.org/licenses/by/4.0/).

Figure S5E. Authors should quantify the western blots for this figure because it has important information. It looks like there is a slight reduction in vac17-S222A/myo2-D1297N even in the absence of Kin4/Frk1.
Figure S7. It would add significantly to the paper if the authors would quantitate the localization of Vac17-GFP in each of the mutants presented.
Figure 8C: The model figure was a little difficult to follow. The authors might consider spelling out vacuole transport rather than using the acronym VTT. Also rather than stating “Mispositioning of vacuole in bud neck…” perhaps the authors would prefer the term “defect in the termination of vacuole transport”, or other terms that have already appeared in the literature.

Comments for the author

In this manuscript, Ekal et al build upon their previous work (Ekal et al 2023) on the identification of SPOC kinases Kin4/Frk1 as important regulators of Myo2-specific cargo adaptors during organelle inheritance. That study showed that Kin4/ Frk1 are involved in promoting peroxisome transit into emerging buds, by preventing premature degradation of the peroxisome adaptor Inp2 in the mother cell. In the current manuscript, the authors find that Kin4/Frk1 also control the levels of the Myo2 vacuole adaptor, Vac17, and that this control takes place in the mother cell. They provide evidence for how this degradation is prevented. Previous studies from others had shown that when Myo2 delivers the vacuole and Vac17 to the bud cortex, a PAK kinase, Cla4 phosphorylates Vac17, which recruits an activator of the E3 ligase, Dma1.
Then the activated Dma1 ubiquitinates Vac17 which subsequently results in Vac17 degradation and the release of Myo2. According to previous studies, this degradation only occurs in the bud via spatial regulation of Cla4 and Dma1. However, here the authors make a convincing case that in the absence of Kin4/Frk1, Cla4 and Dma1 have access to Vac17 in the mother cells. This premature degradation of Vac17 results in a defect in vacuole inheritance. The authors also show that Kin4/Frk1 similarly stabilizes Inp2 by preventing its degradation by Cla4 and Dma1.
Overall, this paper provides a significant advance in current knowledge of Myo2-based organelle transport.

General comments:

Figure 2C. The authors should quantify their western blots so that experiments can be compared. For example, does the bfa1 KO mutation alone lower Vac17 levels?

Figure S4C. The authors should include a quantification of the severity of spindle alignment. Based on the images shown, it appears that kin4/frk1 null cells may also have partial defect.

Line 317-319/Line338-340: The authors conclude that Myo2 and Vac17 interactions are not affected. However, their data would not reveal if binding was partially impaired. A simple pull-down is not sufficient because differences in affinity would not be detected in this assay. The authors should explain that the ability of Vac17 to bind Myo2 might also be impaired. For example, it is possible that kin4/frk1 directly or indirectly impact amino acids within the Myo2-Vac17 binding interface. Along similar lines, the defect in the formation of segregation structures may also be due to a partial defect in Myo2-Vac17 interactions.

Figure 4B/Line 327-331: The authors might want to comment more about the transient nature of the segregation structures in the kin4/frk1 mutant. This could be due to less complexes of Myo2-Vac17 due to less Vac17 levels. In addition, a potential partial defect in Myo2 association with Vac17 may make the complexes less stable.

Figure S5E. The authors are missing some important controls. What are the levels of Vac17 in an otherwise wildtype mutant expressing *vac17-S222A*, also what are the levels of Vac17-S222A in a myo2-D1297N mutant with wildtype Kin4/Frk1.

Figure S5E. Authors should quantify the western blots for this figure because it has important information. It looks like there is a slight reduction in *vac17-S222A/myo2-D1297N* even in the absence of Kin4/Frk1.

© 2024. Published by The Company of Biologists under the terms of the Creative Commons Attribution License (https://creativecommons.org/licenses/by/4.0/).

Figure S7. It would add significantly to the paper if the authors would quantitate the localization of Vac17-GFP in each of the mutants presented.

Figure 8C: The model figure was a little difficult to follow. The authors might consider spelling out vacuole transport rather than using the acronym VTT. Also, rather than stating “Mispositioning of vacuole in bud neck…” perhaps the authors would prefer the term “defect in the termination of vacuole transport”, or other terms that have already appeared in the literature.

First revision

Author response to reviewers' comments

The authors would like to thank the referees for their efforts and suggestions and we have taken them into serious consideration, with many implemented into the revised manuscript. Note that some text was reduced on the request from the Editorial office to follow the word count policy. The version with the modifications highlighted is uploaded as a supplementary file for your reference. Please find below our comments on each point raised by the referees.

Reviewer 1 Comments for the Author:
The manuscript presents a solid piece of work supported by well-performed experiments and data-driven conclusions. However, there are minor points requiring clarification before publication.

Specific points:
1. In Fig 4A, the authors investigate whether Kin4 and Frk1 influence the Myo2-Vac17 interaction via immunoprecipitation and conclude that Kin4 and Frk1 do not influence Vac17- Myo2 interaction. However, Vac17 co-immunoprecipitates with Myo2 at weaker levels than in WT cells. The authors argue that lower Vac17 levels in *kin4Δfrk1Δ* cells could account for reduced Vac17 co-IPed with Myo2. However, it is difficult to exclude from this experiment the possibility that Kin4 and Frk1 may also influence Vac17-Myo2 binding to certain extent. Ideally, the authors should immunoprecipitate Vac17 and quantify the ratio of co-IPed Myo2 in the Vac17-Myo2 complex in WT and *kin4Δfrk1Δ* cells.
→ ***We have quantified Vac17-ProtA in Co-IP experiments and observed reduced complex formation. The amended text in the result section is,***
…”Indeed, co-immunoprecipitation experiments revealed that Vac17-ProtA binds Myo2 in *kin4Δfrk1Δ* cells (Fig. 4A). However, a lower level of the complexes is present and this may lead to inefficient transport to the bud.”…
We have now discussed in the ‘Discussion’ (lines 469-472) that this experiment does not exclude the possibility of an effect of KIN4* and *FRK1 deletion on the Vac17-Myo2 binding affinity in vivo.

2. In Fig. 4C, the transport of the pre-vacuolar membrane (as indicated in lines 334-336) is not clear in the images. Please provide one representative cell in which the transport is evident, albeit short-lived.
→ ***A new panel has been added to the Figure 4B (lower panel), where a representative kin4Δfrk1Δ cell with a vacuole segregation structure is shown.***
3. Fig S1A provides valuable insight into the components analyzed in this study and helps in understanding the experimental logic. I suggest moving it to the main figure.
→ ***S1A is moved to Figure1A. As a consequence, changes to respective Figure numbering and legends were made.***

Reviewer 2 Comments for the Author:
The results presented in this manuscript are exceptional in their quality and abundance, and unequivocally justify the conclusions of the authors. This reviewer sees no need to add additional findings to the manuscript or to modify the figures as they now are. The authors should address the following minor points in their revision:

1) Line 154. Remove ‘the’ before ‘vacuole’.

© 2024. Published by The Company of Biologists under the terms of the Creative Commons Attribution License (https://creativecommons.org/licenses/by/4.0/).

→ ***Done***
2) Line 158. Remove ‘a’ before ‘fresh’.
→ ***Done***
3) Line 258. ‘exponentially’ not ‘exponential’.
→ ***Done***
4) Line 261. Add ‘the’ before ‘T209’.
→ ***Done***
5) Line 296. The reference (Beach et al., 2000) is incorrectly designated. Correct here and in the References.
→ ***Done***
6) Line 725. ‘vacuole positioning’ not ‘peroxisome positioning’.
→ ***Done***
7) Title to Figure S5. The results presented in this figure do not show, but only imply, that Vac17 breakdown is Cla4-/Dnm1-dependent. The authors should therefore remove ‘Cla4-/Dnm1-dependent’.
→ ***Done***
8) Legend to Figure S6, line 6. ‘Mata’ not ‘MatA’.
→ ***Done***

Reviewer 3 Comments for the Author:
Overall, this paper provides a significant advance in current knowledge of Myo2-based organelle transport.

General comments:
Figure 2C. The authors should quantify their western blots so that experiments can be compared. For example, does the bfa1 KO mutation alone lower Vac17 levels?

→ ***Figure 2C blot quantified. Surprisingly, we observed somewhat higher steady-state levels of Vac17-ProtA in bfa1Δ than in WT cells grown on galactose medium (see below) and currently, we are investigating this effect. However, this increase does not affect the interpretation of the experiment, where we compare the stability of Vac17- ProtA in bfa1Δ cells plus and minus galactose-induced overexpression of Kin4. This experiment shows that KIN4 overexpression leads to increased steady-state levels of Vac17-ProtA.***

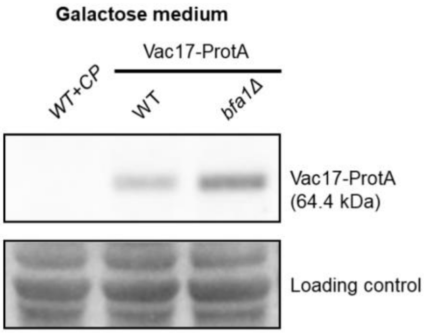

Figure S4C. The authors should include a quantification of the severity of spindle alignment. Based on the images shown, it appears that kin4/frk1 null cells may also have partial defect.
→ ***Quantification for cells with spindle misorientation has been added to the figure. In this experiment, we did not observe a strong defect for kin4Δfrk1Δ cells compared to WT cells.***

Line 317-319/Line 338-340: The authors conclude that Myo2 and Vac17 interactions are not affected. However, their data would not reveal if binding was partially impaired. A simple pull-down is not sufficient because differences in affinity would not be detected in this assay. The authors should explain that the ability of Vac17 to bind Myo2 might also be impaired. For example, it is possible that kin4/frk1 directly or indirectly impact amino acids within the Myo2-Vac17 binding interface. Along similar lines, the defect in the formation of segregation structures may also be due to a partial defect in Myo2-Vac17 interactions.

© 2024. Published by The Company of Biologists under the terms of the Creative Commons Attribution License (https://creativecommons.org/licenses/by/4.0/).

→ ***We have quantified Vac17-ProtA in Co-IP experiments and observed reduced complex formation. The amended text in the result section is,***
…"Indeed, co-immunoprecipitation experiments revealed that Vac17-ProtA binds Myo2 in *kin4Δfrk1Δ* cells (Fig. 4A). However, a lower level of the complexes is present and this may lead to inefficient transport to the bud."…
We have now discussed in the 'Discussion' (lines 469-472) that this experiment does not exclude the possibility of an effect of KIN4* and *FRK1 deletion on the Vac17-Myo2 binding affinity in vivo.

Figure 4B/Line 327-331: The authors might want to comment more about the transient nature of the segregation structures in the kin4/frk1 mutant. This could be due to less complexes of Myo2-Vac17 due to less Vac17 levels. In addition, a potential partial defect in Myo2 association with Vac17 may make the complexes less stable.
→ ***Please see the response to the previous comment.***

Figure S5E. The authors are missing some important controls. What are the levels of Vac17 in an otherwise wildtype mutant expressing *vac17-S222A*, also what are the levels of Vac17-S222A in a myo2-D1297N mutant with wildtype Kin4/Frk1.
Figure S5E. Authors should quantify the western blots for this figure because it has important information. It looks like there is a slight reduction in vac17-S222A/myo2-D1297N even in the absence of Kin4/Frk1.
→ ***The western blot from a new experiment including the controls discussed above along with quantification for the Vac17-ProtA band intensity have been added to the figure. Indeed, there is a slight reduction in vac17-S222A/myo2-D1297N even in the absence of Kin4/Frk1. Kin4 acts by antagonising Cla4-/Dma1-dependent Vac17 degradation. It could be that Kin4 is acting on Dma1 rather than Cla4 but at this stage, we do not have data and tools to convincingly discriminate between these options.***

Figure S7. It would add significantly to the paper if the authors would quantitate the localization of Vac17-GFP in each of the mutants presented.
→ ***Have now included a new panel for the Vac17-GFP localisation for the mutants used in the experiment.***

Figure 8C: The model figure was a little difficult to follow. The authors might consider spelling out vacuole transport rather than using the acronym VTT. Also, rather than stating "Mispositioning of vacuole in bud neck…" perhaps the authors would prefer the term "defect in the termination of vacuole transport", or other terms that have already appeared in the literature.
→ ***The VTT acronym was introduced to reduce the excess text in the figure. However, other suggestions have been included in the modified figure to align more with the terminology in the previous literature.***

<u>Second decision letter</u>

MS ID#: JOCES/2024/261948

MS TITLE: Spatiotemporal regulation of organelle transport by spindle position checkpoint kinase Kin4

AUTHORS: Lakhan Ekal, Abdulaziz M. S. Alqahtani, Kathryn R. Ayscough, and Ewald H. Hettema

We have now reached a decision on the above manuscript.

To see the reviewers' reports and a copy of this decision letter, please go to: https://submit-jcs.biologists.org and click on the 'Manuscripts with Decisions' queue in the Author Area. (Corresponding author only has access to reviews.)

© 2024. Published by The Company of Biologists under the terms of the Creative Commons Attribution License (https://creativecommons.org/licenses/by/4.0/).

As you will see, the reviewers gave favourable reports but raised some critical points that will require amendments to your manuscript. I hope that you will be able to carry these out because I would like to be able to accept your paper, depending on further comments from reviewers.

Please ensure that you clearly highlight all changes made in the revised manuscript. Please avoid using 'Tracked changes' in Word files as these are lost in PDF conversion.

I should be grateful if you would also provide a point-by-point response detailing how you have dealt with the points raised by the reviewers in the 'Response to Reviewers' box. Please attend to all of the reviewers' comments. If you do not agree with any of their criticisms or suggestions please explain clearly why this is so.

Reviewer 1

Advance summary and potential significance to field

The authors addressed all points satisfactorily, and I support the publication of the current version of the manuscript.

Comments for the author

The authors addressed all points satisfactorily, and I support the publication of the current version of the manuscript.

Second revision

Author response to reviewers' comments

The authors would like to thank the referees for their efforts and suggestions and we have taken them into serious consideration, with many implemented into the revised manuscript. The version with the modifications highlighted is uploaded as a supplementary file for your reference. Please find below our comments on each point raised by the referees.

Reviewer 3 Comments for the Author...

The authors did well in addressing most of the prior concerns, however a few issues remain that need to be addressed.

Fig. S4, B and C: In Fig. S4 C, the authors show a gallery of cells where the spindles are normal or misaligned. The images chosen do not have enough examples of large buds to observe whether WT is similar to the double mutant. It looks like the two spindles shown in the double mutant, are much fainter than the WT spindles, and the spindles do not span from the mother to the bud. The authors should add more images of the WT and mutant.

→ ***We have now updated the Fig. S4C panel to have multiple examples with large-budded cells for WT and kin4Δfrk1Δ mutant cells.***

The authors should also clarify and/or provide a detailed schematic of how they exactly scored the misaligned spindles (length, proportion of long spindles vs. puncta relative to bud size, degree of deviation from the mother-bud axis? Since misaligned spindles can only be scored in large buds, the authors could score spindle phenotypes in small vs large buds separately.

→ ***We have now updated the quantification analysis with a schematic diagram for spindle alignment in large-budded cells.***
Long/elongated spindles are scored aligned (purple category) only when spanning from the mother to the bud otherwise they are scored as misaligned (cyan category). Shorter spindles

© 2024. Published by The Company of Biologists under the terms of the Creative Commons Attribution License (https://creativecommons.org/licenses/by/4.0/).

are scored as aligned (blue category)
- If spanning from the mother to the bud
- If not spanning from mother to the bud then scored as aligned only if
1. Angle of the spindle with mother-bud axis < 45º approx and
2. The astral microtubules span through the mother bud neck

We did not quantify spindle phenotypes in small-budded cells as this is not directly relevant to this study.

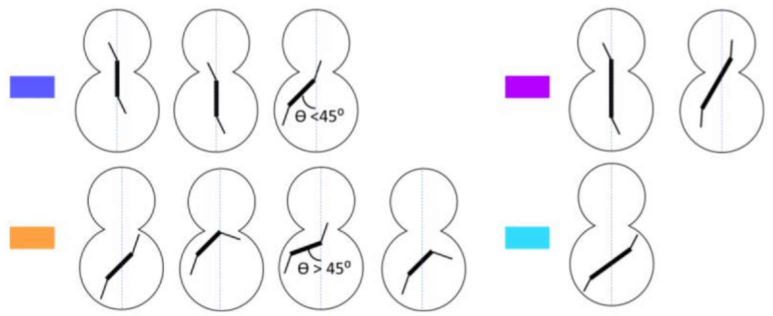

In addition, quantification of the presence of vacuoles in small vs large buds would provide critical support for the conclusion that the role of Kin4 in vacuole inheritance is independent of SPOC regulation.

→ ***We have now added quantification for the presence of vacuoles in small vs large budded cells in WT and kin4Δfrk1Δ cells in Fig S4B panel.***

→ ***We conclude that the role of Kin4 in vacuole inheritance is independent of its role in SPOC based on the following observations,***

First, the SPOC-specific factors Bfa1 and Bub2, that act downstream of Kin4, in MEN regulation are not required for vacuole inheritance (Fig. S4A,B). Second, vacuole inheritance and SPOC-dependent regulation of MEN occur at different times in the cell division cycle as vacuole inheritance starts in small-budded cells (Li et al., 2021) whereas, SPOC can only be triggered to inhibit MEN in large-budded cells (anaphase). In kin4Δfrk1Δ cells, vacuole inheritance defects are already observed in small-budded cells (Fig.1B,D), before SPOC.

We have now updated the Discussion section (lines 473-480).

Fig. 4A/ line 943-945. The authors should have a loading control normalize the input samples in the pull-down experiments, not just Myo2-GFP. The loading control would ensure that the differences in the Co-IP signal are not due to differences in the amount of protein available for IP. Also, the western blots shown in the final figure, are different exposures than the intact blots shown in the supplement. In addition, the amount of Myo2 in the input in Replicate 1 suggest that Myo2 is more stable in the double mutant, but in replicate 2, Myo2 appears to be more stable in the wild-type. This highlights the need for an external control. The annotations for the lanes of the 3rd most panel in replicate 2 are confusing. Only 3 lanes are annotated, but there are at least four lanes where samples were loaded.

→ ***The main objective of the experiments was to test if there is Myo2-Vac17 complex formation in kin4Δfrk1Δ mutant cells. We observed that indeed complexes are present but***

© 2024. Published by The Company of Biologists under the terms of the Creative Commons Attribution License (https://creativecommons.org/licenses/by/4.0/).

that a lower level of Vac17 was coprecipitated with Myo2-GFP from kin4Δfrk1Δ cell lysate compared to wild-type lysate. We corrected for the variations in the amounts Myo2-GFP precipitated. Please find the Co-IP Vac17-ProtA band intensity levels normalised to those in wild-type Co-IP.

**WT**	***kin4Δfrk1Δ***	
**1**	**0.418**	**Replicate 1**
**1**	**0.472**	**Replicate 2**

We think that our analysis provides the evidence for the reduction in the level of Vac17 binding to Myo2 and should be considered in combination with the other data presented.

→ ***We have now updated the blots in Fig 4A and Fig 4A supplement info to match the exposures.***

→ ***In the Replicate 2 blot, there are 3 more lanes with different loading volumes of the same samples. We have now updated the annotations for this blot in the supplementary data.***

Fig. S7 C: The strains all express +Vac17-GFP but this is not clear from the Figure.

→ ***We have now added '+Vac17-GFP' to the Graph's X-axis to indicate that the strains are expressing Vac17-GFP.***

Third decision letter

MS ID#: JOCES/2024/261948

MS TITLE: Spatiotemporal regulation of organelle transport by spindle position checkpoint kinase Kin4

AUTHORS: Lakhan Ekal, Abdulaziz M. S. Alqahtani, Kathryn R. Ayscough, and Ewald H. Hettema

I am happy to tell you that your manuscript has been accepted for publication in Journal of Cell Science, pending standard publication integrity checks.

© 2024. Published by The Company of Biologists under the terms of the Creative Commons Attribution License (https://creativecommons.org/licenses/by/4.0/).
